# Learning Optimal Dynamic Treatment Regimens Subject to Stagewise Risk Controls

**Published:** 2024

**Authors:** Mochuan Liu, Yuanjia Wang, Haoda Fu, Donglin Zeng

**Affiliations:** Department of Biostatistics, University of North Carolina at Chapel Hill, Chapel Hill, NC 27599, USA; Department of Biostatistics, Columbia University, New York, NY 10032, USA; Eli Lilly and Company, Indianapolis, IN 46285, USA; Department of Biostatistics, University of Michigan, Ann Arbor, MI 48109, USA

**Keywords:** Dynamic treatment regimens, Precision medicine, Benefit-risk tradeoff, Acute adverse events, Weighted support vector machine

## Abstract

Dynamic treatment regimens (DTRs) aim at tailoring individualized sequential treatment rules that maximize cumulative beneficial outcomes by accommodating patients’ heterogeneity in decision-making. For many chronic diseases including type 2 diabetes mellitus (T2D), treatments are usually multifaceted in the sense that aggressive treatments with a higher expected reward are also likely to elevate the risk of acute adverse events. In this paper, we propose a new weighted learning framework, namely benefit-risk dynamic treatment regimens (BR-DTRs), to address the benefit-risk trade-off. The new framework relies on a backward learning procedure by restricting the induced risk of the treatment rule to be no larger than a pre-specified risk constraint at each treatment stage. Computationally, the estimated treatment rule solves a weighted support vector machine problem with a modified smooth constraint. Theoretically, we show that the proposed DTRs are Fisher consistent, and we further obtain the convergence rates for both the value and risk functions. Finally, the performance of the proposed method is demonstrated via extensive simulation studies and application to a real study for T2D patients.

## 1. Introduction

Precision medicine aims at tailoring treatments to individual patients by taking their clinical heterogeneity into consideration (Hodson, 2016; Ginsburg and Phillips, 2018). One important treatment strategy in precision medicine is called dynamic treatment regimens (DTRs), which sequentially assign treatments to individual patients based on their evolving health status and intermediate responses (Chakraborty and Murphy, 2014), with the goal of maximizing their long-term rewarding outcome. Over the past years, there has been an explosive development of statistical methods and machine learning algorithms for learning DTRs using either randomized trials (Murphy, 2005; Dawson and Lavori, 2012; Lei et al., 2012), or observational data (Rosthøj et al., 2006; Moodie et al., 2012). Among them, regression-based methods, such as A-learning (Murphy, 2003; Blatt et al., 2004), G-estimation (Robins, 2004), regret regression (Henderson et al., 2010), Q-learning (Qian and Murphy, 2011; Ma et al., 2022), and doubly robust regression (Zhang et al., 2012; Barrett et al., 2014), fit regression models to estimate expected future outcome at each stage, or its varied forms such as blip functions or regrets, and obtain the optimal DTRs by comparing the model-predicted outcomes among treatments in a backward fashion. To lessen the concern of model misspecification, machine learning-based approaches have also been advocated to learn the optimal DTRs by directly optimizing the so-called value function. Examples of machine learning-based methods include outcome weighted learning (OWL) (Zhao et al., 2012, 2015) and its doubly robust extension (Liu et al., 2018), which connects the value optimization problem to a weighted classification problem that can be solved efficiently through support vector machines.

For many chronic diseases, treatments are multifaceted: the aggressive treatment with a better reward is often accompanied by higher toxicity, leading to the elevated risk of severe and acute side effects or even fatality. For example, the Standards of Medical Care in Diabetes published by the American Diabetes Association (ADA) suggests metformin as first-line initial therapy for all general T2D patients. Intensified insulin therapy should be applied to patients when the patients’ A1C level is above the target (American Diabetes Association, 2022). However, evidence has indicated that many patients who may eventually rely on insulin therapy to achieve ideal A1C level will be likely to experience more hypoglycemic episodes (UKPDS Group, 1998), and the latter can cause neurological impairments, coma, or death (Cryer et al., 2003). Thus, the benefit-risk challenge presented in chronic diseases such as T2D entails that the ideal treatment rules should also consider reducing any short-term risks during each decision stage while maximizing the long-term rewarding outcome.

Only a limited number of existing works in DTRs have ever considered the benefit-risk balance, and most of them are restricted to the single-stage decision-making problem. Among them, most of the methods prespecified a utility function to unify the benefit and risk into one composite outcome and proposed to learn optimal decision through maximizing the utility function (Lee et al., 2015; Butler et al., 2018). A major disadvantage of these utility-based approaches is that the choice of the utility function is often subjective and cannot yield decision rules that strictly control the risks. More recently, Wang et al. (2018) reformulated the problem into a constrained optimization problem that maximizes the reward outcome subject to a risk constraint and developed a weighted learning framework for solving the optimal rule. However, no theoretical justification was provided for the proposed method, and extending the framework to study DTRs with multiple-stage risks is nontrivial. Computationally intensive methods have also been proposed to estimate the optimal rules for either competing risks or under a single safety constraint (Laber et al., 2014, 2018), but these methods cannot be easily extended to multiple constraints.

Learning optimal DTRs under the constraints is closely related to constrained and safe reinforcement learning (RL) (Garcıa and Fernández, 2015; Zhao et al., 2023) and multi-objective RL (Hayes et al., 2022), which has attracted much interest in the RL field recently. Most of the safe RL algorithms consider online RL problems and the multi-objective RL aims to learn a policy to achieve the so-called Pareto optimality to balance different outcomes. Furthermore, all these methods are either designed for the problems with a finite state space (Van Moffaert and Nowé, 2014; Chow et al., 2018; Fei et al., 2020; Kalagarla et al., 2021; Wei et al., 2021; Bura et al., 2022; Deng et al., 2023), rely on strong parametric assumptions over policy, transition model and outcome (Mahdavi et al., 2012; Achiam et al., 2017; Yang et al., 2019; Yu et al., 2019; Bohez et al., 2019; Ding et al., 2021; Amani et al., 2021), or study more restrictive bandit problems (Amani et al., 2019; Moradipari et al., 2020; Khezeli and Bitar, 2020). Other methods consider deep neural networks (Ray et al., 2019; Ma et al., 2021) but require a large sample size. Some safe RL for the offline data, including Geibel and Wysotzki (2005); Le et al. (2019); Paternain et al. (2023), are either designed for infinite horizon problems (Geibel and Wysotzki, 2005; Le et al., 2019) or rely on strong model assumptions (Paternain et al., 2023) under CMDP assumption. Compared to all existing work, the DTRs problem to be studied in this work is an offline RL problem with finite horizons, and the applications usually contain only a few hundred subjects.

To address the real-world challenge of treating chronic diseases, in this work, we consider the problem of learning the optimal DTRs in a multistage study, subject to different acute risk constraints at each stage. We develop a general framework, namely benefit-risk DTRs (BR-DTRs), using the finding that under additional acute risk assumption, the stagewise benefit-risk DTRs can be decomposed into a series of single-stage benefit-risk problem only involving the risk restriction of the current stage. Numerically, we propose a backward procedure to estimate the optimal treatment rules: at each stage, we maximize the expected value function under the risk constraint imposed at the current stage, where the solution can be obtained by solving a constrained support vector machine problem. Theoretically, we show that the resulting DTRs are Fisher consistent when some proper surrogate functions are used to replace the objective function and risk constraints. We further derive the non-asymptotic error bounds for the cumulative reward and stagewise risks associated with the estimated DTRs.

Our contributions are two-fold: first, we propose a general framework to estimate the optimal DTRs under the stagewise risk constraints. We note that the proposed framework reduces to the outcome weighted learning for DTRs in Zhao et al. (2015) when there is no risk constraint and reduces to the method in Wang et al. (2018) when there is only one stage. When stagewise risk restrictions are imposed, we show that the backward induction technique adopted in Zhao et al. (2015) along with the single-stage framework proposed in Wang et al. (2018) can be jointly used to solve the optimal DTRs under the stagewise risk constraints. We note that such extension is nontrivial since the treatment of each stage is entangled with unknown treatments of the previous stage through risk constraints when the backward induction technique is used. Hence, additional theoretical justification is needed to rigorously prove that the problem can be decomposed into a series of constrained optimal treatment regimen problems of the current stage under acute risk assumption. Second, our work establishes the non-asymptotic results for the estimated DTRs for both value and risk functions, and such results have never been given before. In particular, we show that support vector machines still yield Fisher consistent treatment rules under a range of risk constraints. Our theory also shows that the convergence rate of the predicted value function is in the order of the cubic root of the sample size, and the convergence rate for the risk control has the order of the square root of the sample size.

The remaining paper is organized as follows. In Section 2, we discuss the statistical framework of BR-DTRs and give the complete BR-DTRs algorithm. In Section 3, we provide further theoretical justification for BR-DRTs. We demonstrate the performance of BR-DTRs via simulation studies in Section 4 and apply the method to a real study of T2D patients in Section 5.

## 2. Method

### 2.1 DTRs under stagewise risk constraints

Consider a T-stage DTRs problem and we use ($Y_{1},\ldots ,Y_{T}$) to denote the beneficial reward and ($R_{1},\ldots ,R_{T}$) to denote the risk outcomes at each stage. We assume that ${\left\{(Y_{t},R_{t})\right\}}_{t=1}^{T}$ are bounded random variables and a series of dichotomous treatment options are available at each stage, denoted by $A_{t}\in \{-1,+1\}$. Let $H_{1}\subset \cdots \subset H_{T}$ be the feature variables at stage t, which includes the baseline prognostic variables, intermediate outcomes, and any time-dependent covariates information prior to stage t. In this work, we further assume that the data is collected from a sequential multiple assignment randomized trial (SMART) (Murphy, 2005) so the treatment assignment probability ${\left\{p(A_{t}|H_{t})\right\}}_{t=1}$ is known for t=1,…,T. Extension to observational studies with unknown treatment assignment probability is discussed in Section 3 (e.g., Remark 7). DTRs are defined as a sequence of functions

$$𝒟={𝒟}_{1}\times \cdots \times {𝒟}_{T}\;:{ℋ}_{1}\times \cdots \times {ℋ}_{T}\to {\left\{-1,+1\right\}}^{T}\;\text{where}\;{𝒟}_{t}\;:{ℋ}_{t}\mapsto \{-1,+1\}.$$

The goal of BR-DTRs is to find the optimal rule ${𝒟}^{*}$ that maximizes the cumulative reward at the final stage T, while the risk at each stage t is controlled by a pre-specified risk constraint, denoted by $\tau_{t}$. Mathematically, we aim to solve the following optimization problem

$$\;\max_{𝒟}\;\;\;E^{𝒟}[\sum_{t=1}^{T}Y_{t}]\;\text{subject to}\;\;E^{𝒟}[R_{1}]\leq \tau_{1},\ldots ,E^{𝒟}[R_{T}]\leq \tau_{T},$$

where E[⋅] denotes the expectation taken w.r.t. the joint distribution of ${\left\{(A_{t},H_{t},Y_{t},R_{t})\right\}}_{t=1}^{T}$ and $E^{𝒟}[\cdot ]$ denotes the expectation given $A_{t}={𝒟}_{t}(H_{t})$ for t=1,..,T.

Additional assumptions are necessary to ensure that the above problem can be solved using the observed data. To this end, we let ${\bar{A}}_{t}=(A_{1},..,A_{t})$ denote the observed treatment history and ${\bar{a}}_{t}={\left(a_{1},\ldots ,a_{t})\in \{-1,+1\right\}}^{t}$ denote any fixed treatment history up to time t, and use $X({\bar{a}}_{t})$ to denote the potential outcome of variable X under treatment ${\bar{a}}_{t}$.

**Assumption 1** (Stable Unit Treatment Value (SUTV)) At each stage, subjects’ outcomes are not influenced by other subjects’ treatment allocation, i.e.,

$$(Y_{t},R_{t})=(Y_{t}({\bar{a}}_{t}),R_{t}({\bar{a}}_{t}))\;\;given\;\;{\bar{A}}_{t}={\bar{a}}_{t}.$$

**Assumption 2** (No Unmeasured Confounders (NUC)) For any t=1,…,T,

$$A_{t}⫫(H_{t+1}({\bar{a}}_{t}),\ldots ,H_{T}({\bar{a}}_{T-1}),Y_{T}({\bar{a}}_{T}),R_{T}({\bar{a}}_{T}))|H_{t}.$$

**Assumption 3** (Positivity) For any t=1,…,T, there exists universal constants $0<c_{1}\leq c_{2}<1$ such that

$$c_{1}\leq p(A_{t}=1|H_{t})\leq c_{2}\;\;for\;H_{t}\;a.s.$$

**Assumption 4** (Acute Risk) For any t=1,…,T and ${\bar{a}}_{t}\in {\left\{-1,1\right\}}^{t}$, $R_{t}({\bar{a}}_{t})$ only depends on $a_{t}$. In other words, for potential outcome $R_{t}({\bar{a}}_{t})$ we have $R_{t}({\bar{a}}_{t})=R_{t}(a_{t})$.

Assumptions 1 to 3 are standard causal assumptions for DTRs literature and one could refer to Rubin (1978); Robins (1997); Chakraborty and Moodie (2013) for more discussions. In particular, Assumptions 2 and 3 hold if the data are obtained from a SMART. Assumption 4 captures the acute risk property of chronic diseases. That is, for the same individual, the adverse risk in each stage is caused by his/her most recent treatment. We note that Assumption 4 does not imply $R_{t}$ is Markovian and independent of $H_{t}$. In general, $R_{t}$ will be a function of $H_{t}$ for t=1,..,T. As an additional note, we can further assume that $R_{t}$ is positive and bounded away from zero after shifting both $R_{t}$ and $\tau_{t}$ by one same constant without changing the problem of interest.

Under all four additional assumptions and suppose ${𝒟}_{t}(H_{t})\;=\;\text{sign}(f_{t}(H_{t}))$ for some measurable decision function $f_{t}$, we note that

(1) $$E^{𝒟}[R_{t}]=E\left[\frac{R_{t}\prod_{t=1}^{T}I(A_{t}f_{t}(H_{t})\;>\;0)}{\prod_{t=1}^{T}p(A_{t}|H_{t})}\right]=E\left[R_{t}(\text{sign}(f_{1}),\ldots .,\text{sign}(f_{t}))\right]\;\;=E\left[R_{t}(\text{sign}(f_{t}))\right]=E\left[\frac{R_{t}I(A_{t}f_{t}(H_{t})\;>\;0)}{p(A_{t}|H_{t})}\right].$$

Then according to Zhao et al. (2015), the original problem can be reformulated as

(2) $$\max_{(f_{1},\ldots ,f_{T})\in {ℱ}_{1}\times \cdots {ℱ}_{T}}E\left[\frac{\left(\sum_{t=1}^{T}Y_{t})\prod_{t=1}^{T}I(A_{t}f_{t}(H_{t})\;>\;0\right)}{\prod_{t=1}^{T}p(A_{t}|H_{t})}\right]\;\;\text{subject to}\;\;E\left[\frac{R_{t}I(A_{t}f_{t}(H_{t})\;>\;0)}{p(A_{t}|H_{t})}\right]\leq \tau_{t},\;\;\;\;\;t=1,\ldots ,T,$$

where ${ℱ}_{t}$ denotes the set of all real value measurable functions from ${ℋ}_{t}\to R$ and we note that ${\left\{f_{t}\right\}}_{t=1}^{T}$ here is identifiable up to a positive scale. To solve the problem (2), we borrow the idea of Zhao et al. (2015) and introduce the backward induction technique to further decompose the BR-DTRs problem into a series of single-stage single-constraint problems. Let ${\left\{{𝒪}_{t}\right\}}_{t=1}^{T}$ denote the feasible region of the original problem under risk constraints ($\tau_{1},\ldots ,\tau_{T}$) at stage t, i.e.,

𝒪t={f∈ℱt∣E[RtI(Atf(Ht)>0)p(At∣Ht)]≤τt∣},t=1,…,T,

and define the U-function as

Ut(ht;gt,gt+1,…,gT)≔E[∣(∑s=tTYs)∏s=tTI(Asgs(Hs)>0)∏s=tTp(As∣Hs)∣Ht=ht],

where we set $U_{T+1}=0$, then we consider the following iterative optimization problems and their associated optimal solution, denoted by ($g_{1}^{*},\ldots ,g_{T}^{*}$), defined via

(3) $$g_{t}^{*}=\underset{f_{t}\in {𝒪}_{t}}{\text{arg}\;\max}\;E\left[\frac{\left(Y_{t}+U_{t+1}(H_{t+1};\;g_{t+1}^{*},\ldots ,g_{T}^{*}))I(A_{t}f_{t}(H_{t})\;>\;0\right)}{p(A_{t}|H_{t})}\right].$$

When there is no risk constraint, (3) will reduce to the standard OWL framework which is guaranteed to yield optimal solutions for the unconstrained problem following the similar idea as the Bellman equation and Q-learning (Bellman, 1966; Qian and Murphy, 2011). However, extending the backward induction technique to risk-constrained DTRs problems is nontrivial, and the backward induction usually does not yield the optimal solutions for the general problem since the estimation of the treatment of each stage is entangled with unknown treatments from previous stages via the risk constraints. As one of our major contributions, our later proof for Theorem 2 shows that the backward algorithm (3) leads to the optimal solutions of the BR-DTRs problem. To the best of our knowledge, our work is the first to provide the necessary conditions for the optimality of the implementation of the backward induction for stagewise risk-constrained DTRs problems.

**Remark 1** We note that the choice of decision functions ${\left\{f_{t}\right\}}_{t=1}^{T}$ has no restriction and can be chosen from any function class during the estimation such as tree-based models, neural networks, or functions from a reproducing kernel Hilbert space (the last is studied in our work). Also, the definition of ${𝒜}_{t}=\{-1,+1\}$ is only a generic notation, which can depend on $H_{t-1}$ and refer to different treatments in different stages. As an extreme case, ${𝒜}_{t}$ can degenerate to a single treatment in certain stages. In this case, our method can still be applied by restricting the estimation to remaining patients who can receive alternative treatments. This allows us to extend our method to more complicated applications as shown in Section 5.

### 2.2 Surrogate loss and Fisher consistency

One main difficulty of implementing framework (3) is the existence of the indicator functions in both the objective function and risk constraints, which makes solving the original problem NP-hard. Following the idea in Wang et al. (2018), we propose the following surrogate functions to replace both indicator functions: let ϕ(⋅) denote the hinge loss function defined as $\phi (x)={\left(1-x\right)}_{+}$ and ψ(⋅,η) denote the shifted ramp loss function given by

ψ(x,η)={1,ifx≥0x+ηη,ifx∈(−η,0)0,ifx≤−η,}

where η∈(0,1] is a prespecified shifting parameter that can vary with stage. We then consider the following surrogate problem, namely the BR-DTRs problem,

(4) $$f_{t}^{*}=\underset{f_{t}\in {𝒜}_{t}}{\text{arg min}}\;E\left[\frac{\left(Y_{t}+U_{t+1}(H_{t+1};\;f_{t+1}^{*},\;\ldots ,\;f_{T}^{*}))\phi (A_{t}f_{t}(H_{t})\right)}{p(A_{t}|H_{t})}\right],$$

where

𝒜t={f∈ℱt∣E[Rtψ(Atf(Ht),ηt)p(At∣Ht)]∣≤τt}t=1,…,T.

Equivalently, we replace the 0-1 loss function in the objective function with the hinge loss and replace the indicator function in the risk constraint with the shifted ramp loss function. The hinge loss function is a typical choice of the surrogate for 0-1 loss in classification problems such as SVM. The shifted ramp loss can be viewed as a smooth and conservative approximation of the indicator function in the risk constraint function when $\eta_{t}$ is small, which will converge to the true constraint as $\eta_{t}$ goes to 0. As a note, applying BR-DTRs does not require the reward and risk variables to be on the same scale since the solution is the same after rescaling $Y_{t}$ and $R_{t}$ (so $\tau_{t}$ is rescaled too).

For a constrained optimization problem under the 0-1 loss, we say that a surrogate problem is Fisher consistent if the solution to the surrogate problem also solves the original problem under the 0-1 loss. This definition is consistent with the traditional Fisher consistency definition of the unconstrained problem. Our next result shows that the new surrogate problem leads to the DTRs that are Fisher consistent. Before stating the theorem, we define a t-stage pseudo-outcome $Q_{t}$ as

$$Q_{t}=Y_{t}+U_{t+1}(H_{t+1};g_{t+1}^{*},\ldots ,g_{T}^{*}),$$

which is the cumulative reward from stage t to T assuming that all treatments have been optimized from stage t+1 to T. Given ($Q_{t}$, $R_{t}$, $A_{t}$, $H_{t}$) and a=±1, we introduce following notations:

$$m_{Q_{t}}(h,a)=E[Q_{t}|H_{t}=h,A_{t}=a],\;\;\;\delta_{Q_{t}}(h)=m_{Q_{t}}(h,1)-m_{Q_{t}}(h,-1),\;m_{R_{t}}(h,a)=E[R_{t}|H_{t}=h,A_{t}=a],\;\;\;\delta_{R_{t}}(h)=m_{R_{t}}(h,1)-m_{R_{t}}(h,-1).$$

Let

$$\tau_{t,\text{min}}=E\left[R_{t}\frac{I(A_{t}\delta_{R_{t}}(H_{t})\;<\;0)}{p(A_{t}|H_{t})}\right],\;\tau_{t,\max}=E\left[R_{t}\frac{I(A_{t}\delta_{Q_{t}}(H_{t})\;>\;0)}{p(A_{t}|H_{t})}\right].$$

In other words, $\tau_{t,\text{min}}$ is the risk under the decision function given by $-\delta_{R_{t}}(H_{t})$, which is the one minimizing the risk regardless of the reward outcome. Thus, $\tau_{t,\text{min}}$ is the minimum risk that one can possibly achieve at stage t. While $\tau_{t,\max}$ is the risk for the decision function given by $\delta_{Q_{t}}(H_{t})$, which is the one maximizing the reward regardless of the risk. Thus, $\tau_{t,\max}$ is the maximal risk.

**Theorem 2** For t=1,..,T and any fixed $\tau_{t,\text{min}}<\tau_{t}<\tau_{t,\max}$, suppose that $P(\delta_{Q_{t}}(H_{t})\delta_{R_{t}}(H_{t})=0)=0$ and random variable $\delta_{Q_{t}}(H_{t})∕\delta_{R_{t}}(H_{t})$ has a distribution function with a continuous density function in the support of $H_{t}$. Then for any $\eta_{t}\in (0,1]$ and t=1,…,T, we have $sign(f_{t}^{*})=sign(g_{t}^{*})$ almost surely, and ($f_{1}^{*},\ldots ,f_{T}^{*}$) solves the optimization problem in (2).

**Remark 3** Assumption 4 is a key condition for obtaining Theorem 2, which ensures that the original multistage problem can be decomposed into a finite number of single-stage single-constraint subproblems each w.r.t. to the decision function of the current stage. Without this assumption, the solution from each stagewise problem may not necessarily control the risk and the induced risk can be either higher or lower than the risk constraint depending on the relationship between $R_{t}({\bar{a}}_{t})$ and $R_{t}(a_{t})$.

When $\tau_{t}\geq \tau_{t,\max}$, the BR-DTRs problem is reduced to a standard DTRs problem and Zhao et al. (2015) shows that the Fisher consistency holds without additional conditions. For T=1, the conditions are similar to Wang et al. (2018), but they assume $H_{t}$ to have a continuous distribution. Theorem 2 basically indicates that when the risk constraints are feasible and assume that the reward difference between two treatments varies continuously with respect to the risk difference, using the surrogate loss leads to the true optimal DTRs for any shifting parameter $\eta_{t}\;\in \;(0,1]$. The proof of Theorem 2 can be completed by first showing that the surrogate problem (4) yields Fisher consistent rule for T=1 and then proving that the backward induction algorithm (3) yields the optimal solution under Assumption 4. Our proof follows the same sketch where the consistency for T=1 is established in Section A.1.2 and the optimality of the backward induction is established in Section A.1.3. We note that both results are nontrivial and have never been established in the existing literature. The complete proof is presented in Section A.1 in Appendix A.

**Remark 4** The key step to proving Theorem 2 is to derive a closed-form solution to the surrogate problem for T=1. There are three main challenges. First, we consider the Lagrange function of the surrogate problem and obtain its closed-form solution. Second, we show that the optimal solution of the surrogate problem attains some decision boundary, and this is proved using contradiction and careful construction. Third, we show that there exists a Lagrange multiplier yielding the optimal solution to the surrogate problem. The last step entails the continuous density assumption of $\delta_{Q_{t}}(H_{t})∕\delta_{R_{t}}(H_{t})$, which can be implied by the continuity of $Q_{t}$ and $R_{t}$ functions.

### 2.3 Estimating BR-DTRs using empirical data

Given data ${\left\{(H_{i1},A_{i1},Y_{i1},R_{i1},\ldots ,H_{iT},A_{iT},Y_{iT},R_{iT})\right\}}_{i=1}^{n}$ from n i.i.d. patients, we propose to solve the empirical version of the surrogate problem to estimate the optimal DTRs: let

𝒜t,n={f∈𝒢t∣1n∑i=1nRitψ(Aitf(Hit),ηt)p(Ait∣Hit)≤τt∣},

then we solve

(5) $${\hat{f}}_{t}=\underset{f\in {𝒜}_{t,n}}{\text{arg}\;\text{min}}\frac{1}{n}\sum_{i=1}^{n}\frac{\left(\sum_{s=t}^{T}Y_{is})\prod_{s=t+1}^{T}I(A_{is}{\hat{f}}_{s}(H_{is})\;>\;0\right)}{\prod_{s=t}^{T}p(A_{is}|H_{is})}\phi (A_{it}f(H_{it}))+\lambda_{n,t}{\left\|f\right\|}_{{𝒢}_{t}}^{2}$$

for t=T,…,1 in turn. Here, ${\left\|\cdot \right\|}_{{𝒢}_{t}}$ denotes the functional norm associated with functional space ${𝒢}_{t}$. The last term $\lambda_{n,t}{\left\|f\right\|}_{{𝒢}_{t}}^{2}$ is a typical choice of penalty term which regularizes the complexity of the estimated optimal decision function to avoid overfitting. Common choices of ${𝒢}_{t}$ include Reproducing Kernel Hilbert Space (RKHS) under a linear kernel where $k(h_{i},h_{j})=h_{i}^{T}h_{j}$, or a Gaussian radial basis kernel with $k(h_{i},h_{j})=\text{exp}(-\sigma^{2}{\left\|h_{i}-h_{j}\right\|}^{2})$, where σ denotes the inverse of the bandwidth.

A major disadvantage of implementing (5) directly is that subjects whose future stages’ observed treatment do not follow the estimated optimal treatments will be assigned with zero weights, which eliminates their contributions to the estimation of early stages and leads to a considerable loss of sample size as the estimation continues. To overcome this limitation, in this work, we adopt the augmentation technique to further improve the efficiency and stability of the estimation procedure. The augmentation technique was first proposed by Liu et al. (2018) to improve the performance of OWL where the basic idea is to predict the expected reward for subjects’ whose future observed treatments are not optimal. Specifically, we replace the weights in the objective function and treatment variable by

(6) $${\hat{Y}}_{it}=|Y_{it}+{\hat{Q}}_{i,t+1}-{\hat{\mu }}_{t}(H_{it})|,\;\;{\hat{A}}_{it}=A_{it}*\text{sign}(Y_{it}+{\hat{Q}}_{i,t+1}-{\hat{\mu }}_{t}(H_{it})).$$

Here, ${\hat{Q}}_{i,t+1}$ is the augmented Q-function defined as

(7) $${\hat{Q}}_{i,t+1}=\frac{\left(\sum_{s=t+1}^{T}Y_{is})\prod_{s=t+1}^{T}I(A_{is}{\hat{f}}_{s}(H_{is})\;>\;0\right)}{\prod_{s=t+1}^{T}p(A_{is}|H_{is})}\;\;-\sum_{j=t+1}^{T}\left\{\frac{\prod_{s=t+1}^{j-1}I(A_{is}{\hat{f}}_{s}(H_{is})\;>\;0)}{\prod_{s=t+1}^{j-1}p(A_{is}|H_{is})}\left[\frac{I(A_{ij}{\hat{f}}_{j}(H_{ij})\;>\;0)}{p(A_{ij}|H_{ij})}-1\right]{\hat{\mu }}_{t+1,j}(H_{ij})\right\},$$

and let ${\hat{Q}}_{i,T+1}=0$. In expressions (6) and (7), $\left\{\mu_{t}\right\}$ and $\left\{\mu_{t,j}\right\}$ are fixed nuisance functions that need to be provided in advance. Intuitively, $\left\{\mu_{t,j}\right\}$ in the augmented Q-functions are contributions to the loss function for patients whose received treatments are not optimal, and $\left\{\mu_{t}\right\}$ in (6) are introduced to remove the main effect which could further reduce the weight variability without affecting the treatment rule estimation. When constructing the final weight, we flip the sign for both the weight and observed treatment for patients who have negative weights to ensure that all weights are nonnegative, which will lead to the same objective function up to a constant and thus will not affect the estimation. Due to the doubly robust design in the construction of the augmentation terms as shown in Liu et al. (2018), both $\left\{\mu_{t}\right\}$ and $\left\{\mu_{t,j}\right\}$ are allowed to be misspecified and the estimated DTRs will remain to be optimal asymptotically, but a more accurate prediction can potentially lead to more reliable estimation. In practice, $\left\{\mu_{t,j}\right\}$ and $\left\{\mu_{t}\right\}$ usually need to be estimated from observed data. For simplicity, we propose to use the simple least square estimator and minimizing $\sum_{i=1}^{n}{\left(Y_{it}+{\hat{Q}}_{i,t+1}-\mu_{t}(H_{it})\right)}^{2}$ to estimate $\left\{\mu_{t}\right\}$, and estimate $\left\{\mu_{t,j}\right\}$ via solving the weighted least square

(8) $$\frac{1}{n}\sum_{i=1}^{n}\frac{\prod_{s=t}^{T}I(A_{is}{\hat{f}}_{s}(H_{is})\;>\;0)}{\prod_{s=t}^{T}p(A_{is}|H_{is})}\frac{1-p(A_{ij}|H_{ij})}{\prod_{s=t}^{j}p(A_{is}|H_{is})}{\left(\sum_{s=t}^{T}Y_{is}-\mu_{t,j}(H_{ij})\right)}^{2}$$

following Liu et al. (2018). By constructing ${\hat{Q}}_{i,t}$ and replacing the original weight by ${\hat{Y}}_{it}$, the refined procedure can utilize the information from all subjects to estimate the optimal rules across all stages, which will lead to more efficient estimation for DTRs compared with (5).

Hence, we propose a backward procedure to estimate the optimal DTRs based on the refined problem. First, we solve a single-stage problem using data at stage t=T, and then in turn, for t=T−1,…,1, we solve the constrained optimization problem (5) after plugging in (${\hat{f}}_{t+1},\ldots ,{\hat{f}}_{T}$) into (6) and (7) and replacing the weight with ${\hat{Y}}_{it}$. The pseudocode of our final proposed algorithm is presented in Algorithm 1. Finally, since the objective function and the risk constraint in (5) can be both written as the difference between two convex functions, for the optimization at each stage we can apply the difference of convex functions (DC) algorithm (Tao and An, 1997) to iteratively solve the subproblem. In each iteration, the subproblem can be further reduced to a standard quadratic programming problem. Details of the derivation and the implementation of the DC algorithm are presented in Appendix B.

**Table:** 

Algorithm 1 BR-DTRs via Backward Induction
$$\begin{array}{cc} \; & \;\mathrm{Input}:\text{Given training data}\;(Y_{it},R_{it},A_{it},H_{it})\;\text{and}\;(\lambda_{t},{𝒢}_{t},\tau_{t},\eta_{t})\;\text{for}\;i=1,\ldots ,n\;\text{and}\;t=1,\ldots ,T\\ \; & \;\mathrm{for}\;t=T\;\text{to}\;1\;\mathrm{do}\\ \; & \;\;\;\;\mathrm{for}\;j=t+1\;\text{to}\;T\;\mathrm{do}\\ \; & \;\;\;\;\;\;\;\text{obtain estimator}\;{\hat{\mu }}_{t,j}\;\text{via minimizing}\;(8)\\ \; & \;\;\;\;\mathrm{end}\;\mathrm{for}\\ \; & \;\;\;\;\mathrm{if}\;t=T\;\mathrm{then}\;\text{define}\;{\hat{Q}}_{i,T+1}=0\\ \; & \;\;\;\;\mathrm{else}\;\text{compute}\;{\hat{Q}}_{i,t+1}\;\text{from}\;(7)\\ \; & \;\;\;\;\mathrm{end}\;\mathrm{if}\\ \; & \;\;\;\;\text{compute}\;{\hat{\mu }}_{t}\;\text{via least square estimator and obtain}{\left\{({\hat{Y}}_{it},{\hat{A}}_{it})\right\}}_{i=1}^{n}\;\text{via}\;(6)\\ \; & \;\;\;\;\text{obtain}\;{\hat{f}}_{t}\;\text{by solving}\\ \; & \;\;\;\;\;\;\;\;\;\;\;\;\;\;\;\;\;\;\;\;\;\;\;\;\;\;\;\;\underset{f\in {𝒢}_{t}}{\text{min}}\;\frac{1}{n}\sum_{i=1}^{n}\frac{{\hat{Y}}_{it}}{p(A_{it}|H_{it})}\phi ({\hat{A}}_{it}f(H_{it}))+\lambda_{n,t}{\left\|f\right\|}_{{𝒢}_{t}}^{2}\\ \; & \;\;\;\;\;\;\;\;\;\;\;\;\;\;\;\;\;\;\;\;\;\;\text{subject to}\;\frac{1}{n}\sum_{i=1}^{n}\frac{R_{it}}{p(A_{it}|H_{it})}\psi (A_{it}f(H_{it}),\eta_{t})\leq \tau_{t}\\ \; & \;\;\;\;\text{using DC algorithm}\\ \; & \;\mathrm{end}\;\mathrm{for}\\ \; & \;\mathrm{Output}:({\hat{f}}_{1},\ldots ,{\hat{f}}_{T}) \end{array}$$

## 3. Theoretical Properties

In this section, we establish the non-asymptotic error rate of the value function and stagewise risks under the estimated decision functions (${\hat{f}}_{1},\ldots ,{\hat{f}}_{T}$). More specifically, for any arbitrary decision functions ($g_{1},\ldots ,g_{T}$), the value function of ($g_{1},\ldots ,g_{T}$) is defined as

$$𝒱(g_{1},\ldots ,g_{T})=E\left[\frac{\left(\sum_{t=1}^{T}Y_{t})\prod_{t=1}^{T}I(A_{t}g_{t}(H_{t})\;>\;0\right)}{\prod_{t=1}^{T}p(A_{t}|H_{t})}\right].$$

We aim at obtaining the non-asymptotic bound for the regret function given by

$$𝒱(g_{1}^{*},\ldots ,g_{T}^{*})-𝒱({\hat{f}}_{1},\ldots ,{\hat{f}}_{T})$$

and the stagewise risk difference is given by

$$E\left[\frac{R_{t}I(A_{t}{\hat{f}}_{t}(H_{t})\;>\;0)}{p(A_{t}|H_{t})}\right]-\tau_{t},$$

for t=1,…,T.

We assume that ${\left\{{𝒢}_{t}\right\}}_{t=1}^{T}$ are the RKHS generated by the Gaussian radial basis kernel, i.e. ${𝒢}_{t}≔𝒢(\sigma_{n,t})$, where 𝒢(σ) denotes the Gaussian RKHS associated with bandwidth $\sigma^{-1}$. Furthermore, for random variable $Q_{t}$, $R_{t}$, $A_{t}$ and $H_{t}$, we define for a, b∈{−1,1},

$$H_{a,b,t,\tau }=\left\{h\in {ℋ}_{t}\;:a\delta_{Q_{t}}(h)>0,bf_{t,\tau }^{*}(h)>0\right\}$$

and $\Delta_{t,\tau }(h)=\sum_{a,b\in \{-1,1\}}\text{dist}(h,{ℋ}_{t}∕H_{a,b,t,\tau })I(h\in H_{a,b,t,\tau })$, where ${ℋ}_{t}∕H_{a,b,t,\tau }$ denotes the set difference between ${ℋ}_{t}$ and $H_{a,b,t,\tau }$, dist(h,⋅) denotes the Euclidean distance from point h to a set, and $f_{t,\tau }^{*}$ denotes optimal solution of (4) at stage t but replace the risk constraint in ${𝒜}_{t}$ by τ. Note that Theorem 2 implies

$$Q_{t}=Y_{t}+U_{t+1}(H_{t+1};g_{t+1}^{*},\ldots ,g_{T}^{*})=Y_{t}+U_{t+1}(H_{t+1};f_{t+1}^{*},\ldots ,f_{T}^{*}).$$

We assume

**Assumption 5** Let $P_{t}$ denote the distribution of $H_{t}$. For given ($\tau_{1},\ldots ,\tau_{T}$) and any t=1,..,T, there exist universal positive constants $\delta_{0,t}>0$, $K_{t}>0$ and $\alpha_{t}>0$ such that for any $\tau^{\prime }\in [\tau_{t}-2\delta_{0,t},\tau_{t}+2\delta_{0,t}]\subset (\tau_{t,\text{min}},\tau_{t,\max})$ we have

$$\int_{{ℋ}_{t}}\text{exp}\left(-\frac{\Delta_{t,\tau^{\prime }}{\left(h\right)}^{2}}{s}\right)P_{t}(dh)\leq K_{t}s^{\alpha_{t}d_{t}∕2}$$

holds for any s>0.

Assumption 5 is an extension of the Geometric Noise Exponent (GNE) assumption proposed by Steinwart and Scovel (2007) to establish a fast convergence risk bound for standard SVM, and later adopted by Zhao et al. (2012) to derive the risk bound for the DTRs without risk constraints. The GNE assumption can be viewed as a regularization condition of the behavior of samples near the true optimal decision boundary. We note that GNE assumption is implied by Tsybakov’s noisy assumption (Audibert and Tsybakov, 2007), thus weaker than Tsybakov’s noisy assumption (see Theorem 2.6 of Steinwart and Scovel, 2007).

For a fixed $\tau_{t}$, $\alpha_{t}$ can be taken to 1 when $\Delta_{t,\tau }(h)$ has order less or equal to O(h). When the optimal decision boundary is strictly separated, i.e. $\text{dist}(H_{a,b,t,\tau },H_{a^{\prime },b^{\prime },t,\tau })>0$ for any $a\neq a^{\prime }$ and $b\neq b^{\prime }$, by using the fact that $\text{exp}(-t)\leq C_{s}t^{-s}$ one can check that Assumption 5 holds for $\alpha_{t}=\infty$. When the optimal decision boundary is not strictly separated, it can be shown that Assumption 5 can still hold for arbitrary $\alpha_{t}\;\in \;(0,\infty )$ when the marginal distribution of $H_{t}$ has light density near the optimal decision boundary (see Example 2.4 in Steinwart and Scovel (2007)).

The following theorem gives the non-asymptotic error bound for the regret and risk difference for the estimated DTRs, assuming that $\left\{\mu_{t}\right\}$ and $\left\{\mu_{t,j}\right\}$ in the augmentation are known. The theorem allows stage-wise shifting parameters to vary with sample size, denoted by ($\eta_{n,1},\ldots ,\eta_{n,T}$).

**Theorem 5** Suppose that Assumptions 1 to 5 and conditions in Theorem 2 hold, $H_{t}$ is defined on a compact set ${ℋ}_{t}\subset R^{d_{t}}$ for t=1,…,T, and assume that $\left\{\mu_{t}\right\}$ and $\left\{\mu_{t,j}\right\}$ are known functions. Let ${\left\{\nu_{t}\right\}}_{t=1}^{T}$ and ${\left\{\theta_{t}\right\}}_{t=1}^{T}$ be two series of positive constants such that $0<\nu_{t}<2$ and $\theta_{t}>0$ for all t=1,…,T. Then for any n≥1, $\delta_{t}>0$, $\lambda_{n,t}>0$, $\sigma_{n,t}>0$ and $0<\eta_{n,t}\leq 1$, such that $\lambda_{n,t}\to 0$, $\sigma_{n,t}\to \infty$ and that there exist constants $C_{1}$, $C_{2}$, $C_{3}$ satisfying

$$C_{1}\sigma_{n,t}^{-\alpha_{t}d_{t}}\eta_{n,t}^{-1}\leq \delta_{0,t},\;\;C_{2}n^{-1}\sigma_{n,t}^{(1-\nu_{t}∕2)(1+\theta_{t})d_{t}}\leq 1,$$

and $\delta_{t}+C_{1}\sigma_{n,t}^{-\alpha_{t},d_{t}}\eta_{n,t}^{-1}+C_{3}n^{-1∕2}\sigma_{n,t}^{(1-\nu_{t}∕2)(1+\theta_{t})d_{t}∕2}{\left(\frac{M}{c_{1}\lambda_{n,t}}+\sigma_{n,t}^{d_{t}}\right)}^{\nu_{t}∕4}\eta_{n,t}^{-\nu_{t}∕2}\leq 2\delta_{0,t}$, it holds

∣𝒱(f^1,…,f^T)−𝒱(g1∗,…,gT∗)∣≤∑t=1T(c1∕5)1−tCt(n−1∕2λn,t−1∕2σn,t(1−νt∕2)(1+θ1)dt∕2)+(λn,tσn,tdt+σn,t−αtdtηn,t−1+ηn,t+δt)

with probability of at least $1-\sum_{t=1}^{T}h_{t}(n,\sigma_{n,t})$, where

$$h_{t}(n,\sigma_{n,t})=2\;\text{exp}\left(-\frac{2n\delta_{0,t}^{2}c_{1}^{2}}{M^{2}}\right)+2\;\text{exp}\left(-\frac{n\delta_{t}^{2}c_{1}^{2}}{2M^{2}}\right)+\text{exp}\left(-\sigma_{n,t}^{(1-\nu_{t}∕2)(1+\theta_{t})d_{t}}\right).$$

Moreover, with probability at least $1-h_{t}(n,\sigma_{n,t})$, the risk induced by ${\hat{f}}_{t}$ satisfies

$$E\left[\frac{R_{t}I(A_{t}{\hat{f}}_{t}(H_{t})\;>\;0)}{p(A_{t}|H_{t})}\right]\leq \tau_{t}+\delta_{t}+C_{t}n^{-1∕2}\sigma_{n,t}^{(1-\nu_{t}∕2)(1+\theta_{t})d_{t}∕2}\lambda_{n,t}^{-\nu_{t}∕4}\eta_{n,t}^{-\nu ∕2}.$$

Here, $C_{t}$ denotes some constant only depending on $\alpha_{t}$, $K_{t}$, $d_{t}$, $\nu_{t}$, $\theta_{t}$, $c_{1}$ and M.

Theorem 5 can be established by first verifying the result for T=1 and then extending the result to T≥2 using an analogous argument of Theorem 3.4 of Zhao et al. (2015). The risk bound of the value function proved in Theorem 5 indicates that the error consists of four parts. The first two terms correspond to the stochastic error and approximation error resulting from using the empirical estimator to approximate the true objective function and restricting the estimated decision functions within the Gaussian RKHS in the empirical problem. The third error term $O(\sigma_{n,t}^{-\alpha_{t}d_{t}}\eta_{n,t}^{-1})$ is induced by using the empirical estimator as risk constraints in (5). The remaining error has order $O(\eta_{n,t})$ and results from the property that the regret under 0-1 loss function is upper bounded by the regret under hinge loss plus an error term of order O(η) when we use the shifted ramp loss to approximate the indicator function in constraints. Due to the existence of the last two error terms, the choice of shifting parameter must be small but bounded away from 0 in order to minimize the regret. The proof of Theorem 5 and required preliminary lemmas are provided in Section A.2 in Appendix A.

According to Theorem 5, the risk bound of the regret is minimized by setting $\eta_{n,t}=\sigma_{n,t}^{-\alpha_{t}d_{t}}\eta_{n,t}^{-1},\lambda_{n,t}\sigma_{n,t}^{d_{t}}=\sigma_{n,t}^{-\alpha_{t}d_{t}}\eta_{n,t}^{-1}$ and $\eta_{n,t}=n^{-1∕2}\lambda_{n,t}^{-1∕2}\sigma_{n,t}^{(1-\nu_{t}∕2)(1+\theta_{t})d_{t}∕2}$, which gives

$$\lambda_{n,t}=O(\sigma_{n,t}^{-(\alpha_{t}+2)d_{t}∕2}),\;\;\eta_{t}=O(\sigma_{n,t}^{-\alpha_{t}d_{t}∕2})$$

and

$$\sigma_{n,t}=O(n^{\frac{1}{\alpha_{t}d_{t}+(\alpha_{t}+2)d_{t}∕2+(1-\nu_{t}∕2)(1+\theta_{t})d_{t}}}).$$

Consequently, there exists constants $k_{1}$, $k_{2}>0$ independent of sample size n such that

$$|𝒱({\hat{f}}_{1},\ldots ,{\hat{f}}_{T})-𝒱(g_{1}^{*},\ldots ,g_{T}^{*})|\leq k_{1}\sum_{t=1}^{T}{\left(c_{1}∕5\right)}^{1-t}n^{-\frac{\alpha_{t}d_{t}}{2\alpha_{t}d_{t}+(\alpha_{t}+2)d_{t}+2(1-\nu_{t}∕2)(1+\theta_{t})d_{t}}}$$

holds with probability $1-\sum_{t=1}^{T}\text{exp}\;(-k_{2}n^{\frac{(1-\nu_{t}∕2)(1+\theta_{t})d_{t}}{\alpha_{t}d_{t}+(\alpha_{t}+2)d_{t}∕2+(1-\nu_{t}∕2)(1+\theta_{t})d_{t}}})$. When $\alpha_{t}$ can be selected arbitrarily large in which case the data are approximately separated near the optimal decision boundary, the convergence rate of the value function is at most of order $O(n^{-1∕3})$. In terms of risks, when $\alpha_{t}$ can be arbitrarily large and let $\nu_{t}$ go to 0, the risk constraint inequality indicates that the stagewise risk under the estimated rule can always be bounded by $\tau_{t}$ plus an error term of order up to $O(n^{-1∕2})$. In terms of stage T, we note that the error bound is increasing exponentially with respect to the total number of stages. This result is similar to the risk bound of value function obtained in Q-learning (Murphy, 2005) and OWL (Zhao et al., 2015). In practice, the optimal choice of tuning parameters ${\left\{\lambda_{n,t}\right\}}_{t=1}^{T}$, ${\left\{\sigma_{n,t}\right\}}_{t=1}^{T}$ and ${\left\{\eta_{n,t}\right\}}_{t=1}^{T}$ can be obtained via cross-validation.

**Remark 6** Note that the result in Theorem 5 is obtained under the assumption that $\left\{\mu_{t}\right\}$ and $\left\{\mu_{t,j}\right\}$ are known and fixed functions. As discussed in Section 2.3, in practice functions $\left\{\mu_{t}\right\}$ and $\left\{\mu_{t,j}\right\}$ usually need to be estimated from observed data. Since the value function is Lipschitz continuous in terms of the model parameters $\left\{\mu_{t}\right\}$ and $\left\{\mu_{t,j}\right\}$, when $\left\{\mu_{t}\right\}$ and $\left\{\mu_{t,j}\right\}$ are estimated by prespecified parametric models as adopted in our proposed algorithm, the estimation will only induce an additional variability of order $O(n^{-\frac{1}{2}})$, which will be dominated by the error bounds in Theorem 5 and, hence, will not affect the conclusion.

**Remark 7** The result obtained in Theorem 5 can also be generalized to observational study when the treatment assignment probabilities are unknown and need to be estimated from the observed data. Similar to the previous remark, when $p(A_{t}|H_{t})$ are estimated by parametric models such as logistic regression, such estimation will only induce an additional variability of order $O(n^{-\frac{1}{2}})$, which will not affect the non-asymptotic error obtained in Theorem 5. When the treatment assignment probability is estimated at a slower rate, the additional variability can be accounted for through additional expansion of the objective function on these parameters.

## 4. Simulation Studies

We demonstrate the performance of BR-DTRs via simulation studies in this section. We consider two settings both of which simulate the situation when adopting preferable treatment in the early stage would immensely affect the performance of possible treatments in later stages. Specifically, in both settings, we first generate an 8-dimensional baseline prognostic variable matrix X from independent uniform distribution U[0,1]. In the first setting, we consider a two-stage randomized trial where treatments $A_{1}$ and $A_{2}$ are randomly assigned with an equal probability of 0.5. The stage-specific rewards and risks are defined by

$$\;Y_{1}=1-X_{1}+A_{1}(-X_{1}-X_{2}+1)+\epsilon_{Y_{1}},\;\;R_{1}=2+X_{1}+A_{1}(-X_{1}∕2+X_{2}+1)+\epsilon_{R_{1}},\;Y_{2}=1-X_{1}+A_{2}(Y_{1}-3X_{1}+A_{1}+1)+\epsilon_{Y_{2}},\;\;R_{2}=1+X_{1}+A_{2}(Y_{2}∕2-X_{1}+A_{2}∕2+1)+\epsilon_{R_{2}},$$

where $\epsilon_{Y_{1}}$, $\epsilon_{Y_{2}}$ are noises of reward outcomes generated from the independent standard normal distribution N(0,1), and $\epsilon_{R_{1}}$, $\epsilon_{R_{2}}$ are noises of adverse risks generated from the independent uniform distribution U[−0.5,0.5]. In this setting, both $Y_{1}$, $Y_{2}$, $R_{1}$ and $R_{2}$ are the linear functions of $H_{1}=X$ and $H_{2}=(H_{1},A_{1},Y_{1},R_{1})$. In the second setting, $Y_{2}$ is a nonlinear function of $H_{2}$ and is generated according to

$$\;Y_{1}=1+A_{1}(-X_{1}-X_{2}∕3+1.2)+\epsilon_{Y_{1}},\;\;R_{1}=1.5+A_{1}(-X_{1}∕3+1.5)+\epsilon_{R_{1}},\;Y_{2}=1+A_{2}(-X_{1}^{2}∕2-X_{2}^{2}∕2+3A_{1}∕2+1.5)+\epsilon_{Y_{2}},\;\;R_{2}=1+A_{2}(2A_{1}+2)+\epsilon_{R_{2}},$$

and ($A_{1}$, $A_{2}$, $\epsilon_{Y_{1}}$, $\epsilon_{Y_{2}}$, $\epsilon_{R_{1}}$, $\epsilon_{R_{2}}$) are generated the same way as setting I. Note that for setting II, the optimal decision boundary in stage II is a circle w.r.t. ($X_{1}$, $X_{2}$).

For each simulation setting, we implement our proposed method with training data sample size n equal to 200 and 400. We let $\eta =\eta_{1}=\eta_{2}$ varying from 0.02 to 0.1 with an increment of 0.02. For the first simulation setting, we repeat the simulation for $\tau_{1}=\tau_{2}=1.4$ and 1.5; for the second simulation setting, we repeat the simulation for $\tau_{1}=\tau_{2}=1.3$ and 1.4. Both the linear kernel and the Gaussian kernel are employed to compare their performance. We conduct the estimation following exactly the same description in Section 2.3 and the tuning parameter $C_{n,t}={\left(2n\lambda_{n,t}\right)}^{-1}$ will be selected by a 2-fold cross-validation procedure that maximizes the Lagrange dual function from a pre-specified grid of 2^−10^ to 2^10^. To alleviate the computational burden, when using the Gaussian kernel we follow the idea of Wu et al. (2010) and fix $\sigma_{n,t}^{-1}$ to be $2*\text{median}\{\|H_{it}-H_{jt}\|\;:A_{it}\neq A_{jt}\}$ instead of picking $\sigma_{n,t}$ adaptively according to n and other tuning parameters. In our simulations, all feature variables will be re-centered to mean 0 and rescaled into interval [−1, 1]. When solving the optimization problem, we choose the initial values for parameters either uniformly in a bounded interval or using the estimated parameters from the unconstrained problem. We recommend the latter approach as the performance is overall better than picking the initial point randomly. All quadratic programming programs in the DC procedure will be solved by R function *solve.QP()* from *quadprog* package (https://cran.r-project.org/web/packages/quadprog/index.html). As a comparison, we also implement the AOWL method proposed by Liu et al. (2018) as implemented in package *DTRlearn2* (https://cran.r-project.org/web/packages/DTRlearn2/index.html), which ignores the risk constraints. In addition, we also compare our method with the naive approach where in stage I, we simply use $Y_{1}+Y_{2}$ as the outcome for estimation without adjusting for any delayed treatment effects even though the risk constraints are considered. To assess the performance of each method, we calculate the stage optimal estimated reward and risk on an independent testing dataset of size $N=2\times 10^{4}$. We repeat the analysis with 600 replicates.

**Figure 1:**
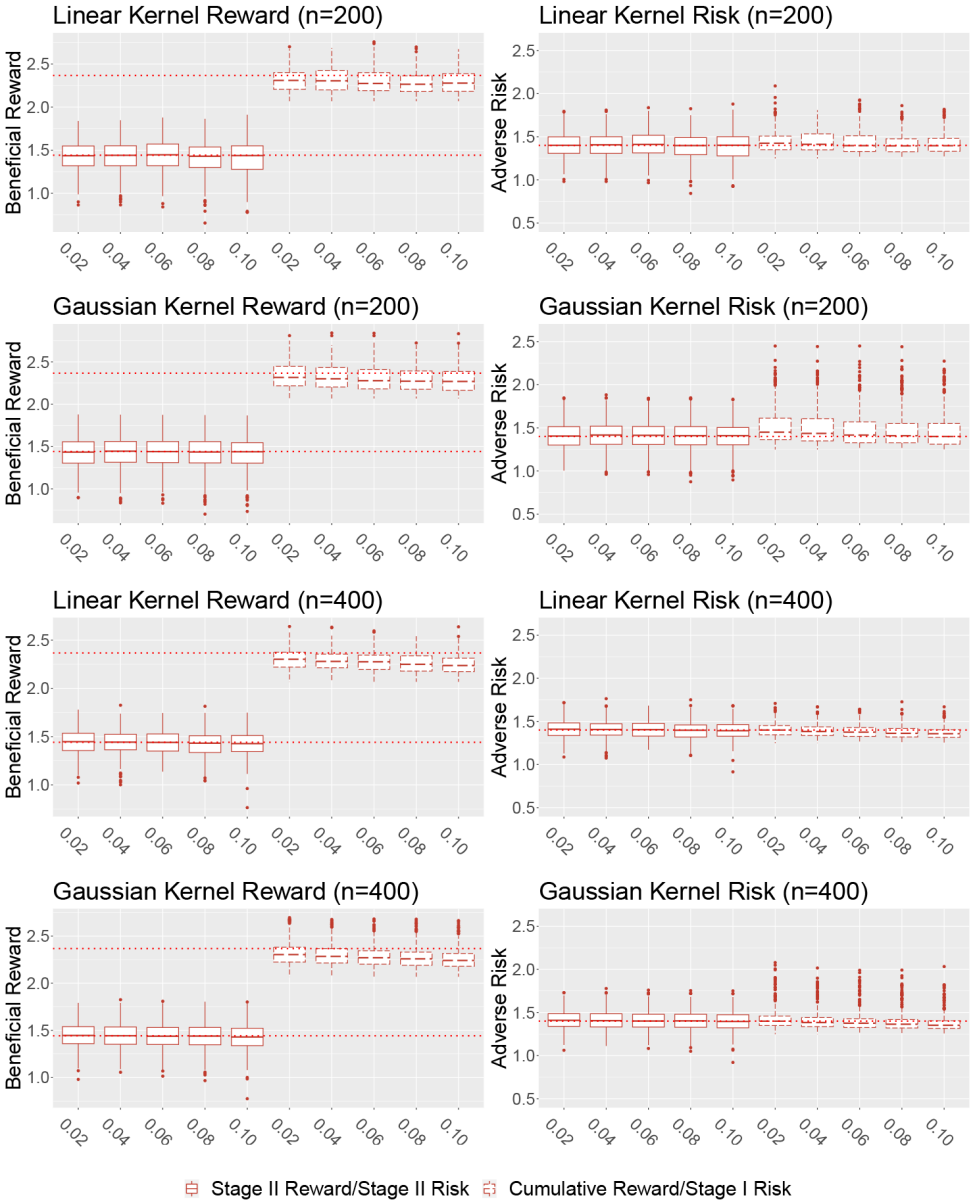
Estimated reward/risk on independent testing data set for simulation setting I, training sample size n={200,400} and η={0.02,0.04,…,0.1} (x-axis) under linear kernel or Gaussian kernel. The dashed line in reward plots refers to the theoretical optimal reward under given constraints. The dashed line in risk plots represents the risk constraint τ=1.4.

**Figure 2:**
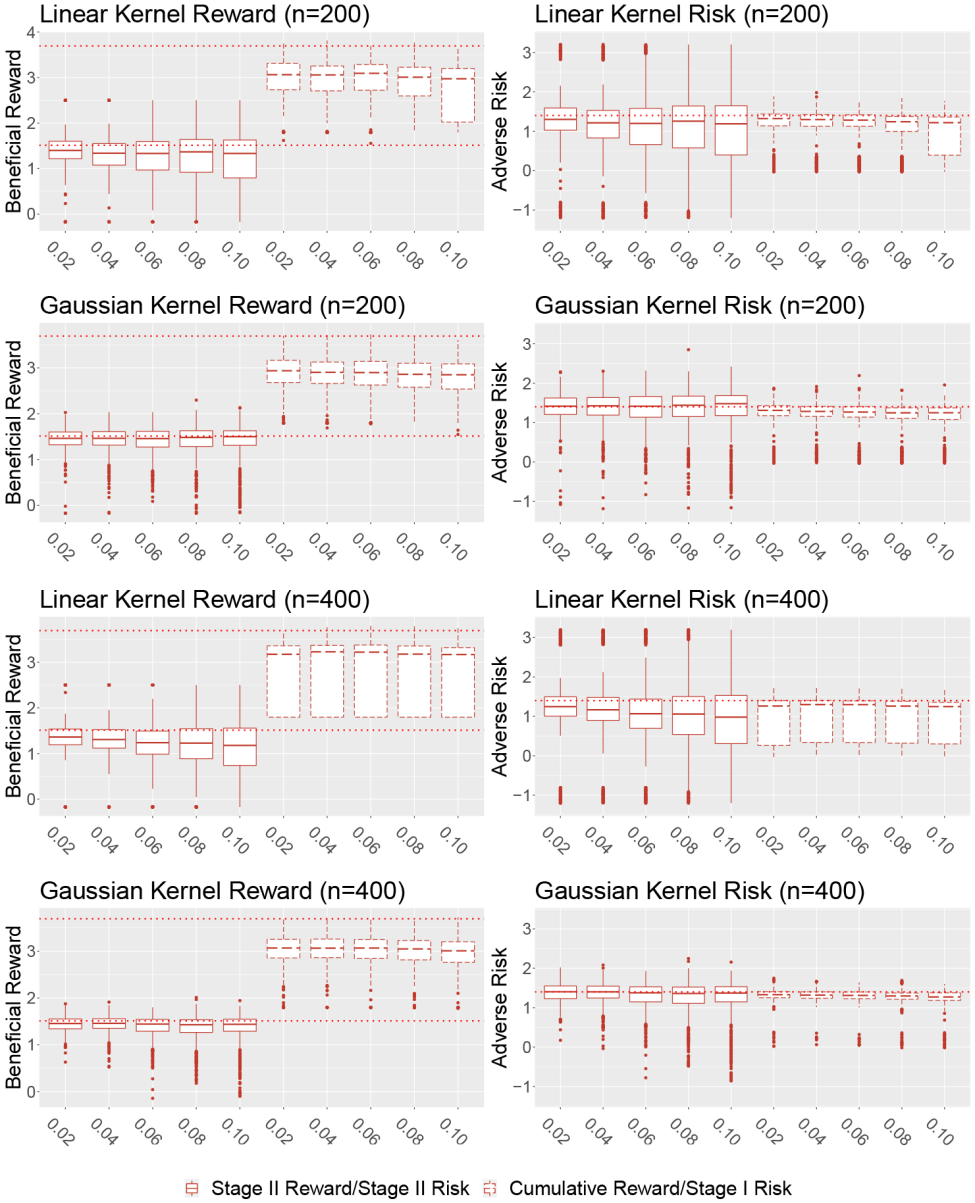
Estimated reward/risk on independent testing data set for simulation setting II, training sample size n={200,400} and η={0.02,0.04,…,0.1} (x-axis) under linear kernel or Gaussian kernel. The dashed line in reward plots refers to the theoretical optimal reward under given constraints. The dashed line in risk plots represents the risk constraint τ=1.4.

Figure 1 displays the estimated reward and risk on the independent testing data for the first simulation setting under the different choices of training sample size, kernel basis, and shifting parameter η for $\tau_{1}=\tau_{2}=1.4$. From the plot, we notice that for the simple linear setting, under both linear and Gaussian kernel the median values of estimated reward/risk will be close to the theoretical reward/pre-specified risk constraints. This indicates that the proposed method can successfully maximize the reward while controlling the risks across both stages. In this setting, compared with the linear kernel, using the Gaussian kernel will significantly underestimate the risk on training data, leading to somewhat exceeding risk on the testing data. Also as expected, in this setting increasing sample size would improve the performance under both kernel choices. In terms of the shifting parameter η, in setting I there is no obvious preference for choosing a small value to a large value. The result from the second nonlinear simulation setting under $\tau_{1}=\tau_{2}=1.4$ is presented in Figure 2. Under this more complicated setting and when both two stages’ optimal decision boundaries are nonlinear, we notice that our method still yields a value close to the truth and the risks are reasonably controlled in both stages. The Gaussian kernel outperformed the linear kernel in both stages since using the linear kernel will misspecify the true model. When the sample size increased, the performance for the Gaussian kernel improved but it was not necessary for the linear kernel, likely due to the misspecification. We also observe that under the second simulation setting and when the Gaussian kernel is used, choosing a small shifting parameter η will achieve better performance on the testing data with much smaller variability. The results for τ=1.5 for setting I and τ=1.3 for setting II are similar to τ=1.4 already discussed. An additional simulation study is conducted to investigate the performance of the proposed method in an observational study by assuming that the treatment assignment probability is unknown and estimated from data. Similar conclusions can be made in this setting. All additional results are presented in Appendix C.

Finally, the results in Table 1 compare the performance of BR-DTRs to AOWL, which ignores the risk constraints, and the naive method, which considers the risk constraints but uses the immediate outcomes as the reward. Clearly, even though AOWL always gives a higher reward than BR-DTRs, the corresponding risks of applying the estimated treatment rules are much larger than the ones from BR-DTRs. In contrast, BR-DTRs can always give valid decision rules with risks close to pre-specified threshold values. When compared with the naive method, due to the nature of DTRs, the reward of the BR-DTRs method is always higher than the naive method. In terms of the algorithm complexity, as a benchmark, the median running times for completing one estimation with fixed tuning parameters $C_{n,1}=C_{n,2}=1$ and fixed shifting parameter η=0.02 under setting I (n=200 and $\tau_{1}=\tau_{2}=1.4$) are 4.79 and 3.13 minutes for linear or Gaussian kernel, respectively. For setting II, the running times for n=200 and $\tau_{1}=\tau_{2}=1.4$ are 3.37 and 3.54 minutes, respectively. When the sample size is increased to n=400, the median running time will increase to 18.71 and 16.41 minutes for setting I, and 8.46 and 10.27 minutes for setting II. When the sample size is large, the running time can be reduced by using stochastic gradient-based methods to speed up the quadratic optimization in each DC iteration or implementing gradient-based methods to solve the constrained non-convex optimization directly.

## 5. Real Data Application

We apply BR-DTRs to analyze the data from the DURABLE study (Fahrbach et al., 2008). The DURABLE study is a two-phase trial designed to compare the safety and efficacy of insulin glargine versus insulin lispro mix in addition to oral antihyperglycemic agents in T2D patients. During the first phase trial, patients were randomly assigned to the daily insulin glargine group or twice daily insulin lispro mix 75/25 (LMx2) group for 24 weeks. By the end of 24 weeks, patients who failed to reach an HbA1c level lower than 7.0% would enter the second phase intensification study and be randomly reassigned with either basal-bolus therapy (BBT) or LMx2 for insulin glargine group or basal-bolus therapy (BBT) or three times daily insulin lispro mix 50/50 (MMx3) therapy for LMx2 group. Any other patients who reached HbA1c 7.0% or lower would enter the maintenance study and keep the initial therapy for another 2 years. A flowchart of the study design of the DURABLE trial is provided in Appendix D for reference.

In the DURABLE study, the major objective is lowering patients’ endpoint blood glucose level measured in HbA1c level, and in this analysis, we use the reduction of HbA1c level at 24 weeks since baseline as the reward outcome for the first stage and use the reduction of HbA1c level at 48 weeks since 24 weeks as the reward outcome for the second stage. The risk outcome is set to be hypoglycemia frequency encountered by patients, which reflects the potential risk induced by adopting assigned treatment. Patients who achieved an HbA1c level lower than 7% and entered the maintenance study would not be re-randomized with new treatments during the second stage. To accommodate these patients in our proposed framework, we make the additional assumption that for patients in the maintenance study, their first-stage treatment is already optimal and should not be adjusted. This assumption is consistent with the general guidance of treating T2D patients suggested by ADA where the patient’s treatment should be unchanged if the patient’s HbA1c level can be maintained lower than 7% (American Diabetes Association, 2022). Under this assumption, in the second stage, patients in the maintenance study are already receiving optimal treatment so it is not necessary to estimate their optimal decision rules. Consequently, the second stage analysis will only involve patients who entered the intensification study, and only in the first stage will all patients be included in the analysis. In the first stage estimation, for patients in the maintenance study, their future reward outcome (reduction of HbA1c) is assumed to be maintained. That is, in Stage I, the reward outcome becomes

Y′={Y,if subject is from the maintenance studyYI(A2f^2(H2)>0)0.5,if subject is from the intensification study.}

Finally, the second stage risk outcome is the total frequency of hyperglycemia events during the intensification study (from 24 weeks to 48 weeks) and the first stage risk outcome is defined to be the total hypoglycemia events from week 0-24 for patients who entered intensification study, and the total hypoglycemia events from week 0-48 rescaled to 24 weeks for the remaining patients who entered maintenance study. In the analysis, we eventually apply the logarithm transformation to these counts to handle some extremely large counts in the data.

We consider 20 relevant covariates as the baseline predictors $H_{1}$, including HbA1c testing result, heart rate, systolic/diastolic blood pressures, body weight, body height, BMI, and 7 points self-monitored blood glucose measured at baseline (week 0) along with patient’s age, gender, duration of T2D and 3 indicator variables indicating whether patients were taking metformin, thiazolidinedione, or sulfonylureas. The second stage predictors $H_{2}$ include all predictors in $H_{1}$, patient’s treatment assignment, the cumulative number of hyperglycemia events during the first stage, along with heart rate, systolic/diastolic blood pressures, HbA1c and same 7 points self-monitored blood glucose measured at the initial time of the second stage (24 weeks). All covariates are centered at mean 0 and rescaled to be within [−1, 1].

The final study cohort includes 579 patients from the intensification study and another 781 from the maintenance study. To compare the performance, we randomly sample 50% patients from the intensification study as the training sample for stage II and an additional 50% patients from the maintenance study as the training sample for stage I. The remaining patients will be treated as the testing data to assess the performance of the estimated rules. We consider different risk constraints $\tau_{2}=(0.334,\infty )$ and $\tau_{1}=(0.893,0.948,1.005)$ where we rescale the risk to hypoglycemia events per 4 weeks. We note that 0.334 and 0.948 are the mean risks of stage II and stage I, respectively, and 1.005 is close to the median estimated risk on testing data under the unconstrained case. We repeat the analysis 100 times for random splitting of the training and testing data. For our method, we also conduct the estimation following the description in Section 2.3 and use the Gaussian kernel and choose η=0.02, while tuning parameter ${\left\{C_{n,t}\right\}}_{t=1}^{2}$ for each stage will be selected by two-fold cross-validation similar to the simulation studies. The bandwidth of the Gaussian kernel is also selected similar to the simulation studies.

All real data analysis results are displayed in Table 2. From Table 2 we first notice that in each stage, the median estimated risk on testing data is tightly controlled by the prespecified risk constraints. This demonstrates that BR-DTRs can also successfully control adverse risks in real applications. Under each risk constraint, the cumulative reward estimated by BR-DTRs is only slightly better or closed against the estimated reward using the naive method. One reason is that the majority of the patients in stage I would not enter the intensification study and, hence, have no delayed treatment effect at all.

Among all 7 constraint settings, the uncontrolled setting, as expected, produces the estimated rules with both the highest reward and risks, and the estimated reward decreases as the risk constraint of either stage decreases. Under the unconstrained estimated optimal rules, all patients are recommended to receive LMx2 in the first stage and later switch to MMx3 after 24 weeks if patients’ HbA1c level is greater than 7.0% by the end of the first phase. As a comparison, when the risk constraint is imposed in stage II, the optimal rules will instead recommend all patients to receive BBT when patients fail to reach HbA1c lower than 7.0% in the second stage at a price of significantly lower reduction in HbA1c by the end of 48 months. Similar treatment preference change happens in stage I as the optimal estimated rule becomes less favorable to LMx2 against insulin glargine when $\tau_{1}$ decreases.

Comparing the reward and risks under different choices of risk constraint, $\tau_{1}=1.005$ and $\tau_{2}=\infty$ produce the second highest reward with moderate risk in the second stage and 10% lower risk in the first stage compared to the unconstrained setting. Under this suboptimal setting, the estimated rules recommend only 50.7% of patients start with LMx2 therapy and later switch to MMx3 therapy if patients fail to reach an HbA1c level of less than 7.0% by the end of the first phase of treatment. By checking the baseline covariates between the patients who received different treatment recommendations, under this estimated rule for the patients whose baseline HbA1c falls in the range [7, 8), [8, 9) and [9, 10), the proportion of the patients who are recommended with LMx2 therapy drops from 62.7% to 56.3% and 46.3%; similarly, for the patients whose baseline BMI falls in the range [28, 32), [32, 34) to [34, 36), the proportion of patients recommended with LMx2 also drops from 59.3% to 53.8% and 51.3%. The negative correlation between the increment of baseline HbA1c/BMI against the proportion of patients recommended with LMx2 as the first phase treatment indicates that the patients with a worse initial health condition are less likely to be recommended with LMx2 therapy as the initial treatment when the risk impact is considered. This is consistent with the fact that LMx2 is a more intense therapy compared with insulin glargine therapy and would cause more hypoglycemia events among unhealthier T2D patients. In particular, the suboptimal rules obtained from BR-DTRs meet the ADA guidance which suggests that intensive insulin therapy should be prescribed to patients according to patients’ health condition to reduce potential hypoglycemia events. In conclusion, the real data application demonstrates that, by evaluating the impact of adverse risks along with beneficial reward, BR-DTRs can produce better personalized, more practically implementable treatment recommendations compared with standard OWL which only takes beneficial reward into consideration.

## 6. Discussion

In this work, we introduce a new statistical framework BR-DTRs to estimate the optimal dynamic treatment rules under the stagewise risk constraints. Sufficient conditions are provided to guarantee the Fisher consistency of using backward induction to learn the optimal decision rules of DTRs problems under stagewise risk constraints. The backward induction technique provides an algorithm to solve BR-DTRs efficiently through iteratively solving a series of single-stage, single-constraint sub-problems. In addition, we establish the non-asymptotic risk bound for the value and stagewise risks under the estimated decision functions. Our theoretical contributions include providing sufficient conditions for implementing backward induction for the constrained decision-making problem and non-asymptotic performance guarantee under the estimated rules.

To tackle the numerical challenge due to the 0-1 loss, we introduced the hinge loss and shifted ramp loss as the surrogate losses in this work. We note that although the shifted ramp loss could also be used as the surrogate function for the objective function, it does not reduce to a standard SVM problem when $\tau_{t}$ is infinity and involves an additional tuning parameter. More numerical comparisons with the alternative choices of the surrogate functions are necessary.

It is worth noting that even though we focus on handling DTRs problems, the proposed method is applicable and can be generalized to other sequential decision-making problems beyond biomedical research. One example is the promotion recommendation in E-commerce, where the goal is to learn a personalized strategy that maximizes customers’ buying willingness at a tolerable loss of revenue (Goldenberg et al., 2021; Wang et al., 2023). In this application, multiple waves of promotions are scheduled to be delivered to customers in a cycle (Chen et al., 2022) and BR-DTRs can be applied to learn the optimal strategies at each stage. In Appendix C.3, an additional simulation study mimicking such promotion recommendation problem has been conducted for T=4 and the results indicate that the BR-DTRs method still performs well. Moreover, even though we assumed treatments to be dichotomous and only one risk constraint is imposed at each stage in BR-DTRs, our method can also be extended to problems with more treatment options and risk constraints at each stage. One can achieve this by imposing multiple smooth risk constraints to multicategory learning algorithms, such as angle-based learning methods Qi et al. (2020); Ma et al. (2023). However, verifying the Fisher consistency of generalized problems is not trivial and is beyond the scope of this work. In addition, for many real world applications, finding the most influential feature variables that drive the optimal decisions is of equal importance as obtaining the explicit rules that maximize the beneficial reward under the constraints. Thus, BR-DTRs can also be extended to incorporate feature selection during the estimation. For example, when the RKHS is generated by the linear kernel, the optimal decision boundary is linear, and one can introduce an additional penalty term with a group structure to impose sparsity over feature variables.

There are several limitations of the proposed method. One limitation is that the proposed method may not perform well for a very large number of horizons. For example, the uncertainty for the objective maximization is accumulated over stages in the backward algorithm, so it will increase for large T. In contrast, as shown in Theorem 5, the uncertainty for the risk control at each stage will remain independent of T. Consequently, the risk constraint will mainly drive the decision rules for large T, which may not be the ideal solution in practice. Possible extensions can be to impose appropriate parametric assumptions on the DTRs, or less strict control on the risk function. Another limitation is the acute risk assumption, which requires the stagewise risk to be solely determined by the most recent action. However, this assumption may be violated in some applications when risks are expected to be affected by earlier actions. For example, the stagewise risks can be defined as the total number of the most toxic treatments received since the beginning of the treatments. Therefore, further extensions are necessary when the delayed risks exist.

## Figures and Tables

**Table 1:** Estimated reward/risk on independent testing data for $\tau_{1}=\tau_{2}=1.4$ and n=400 under 3 different methods using linear/Gaussian kernel.

Setting	η	Method	Linear Kernel				Gaussian Kernel			
			Reward - II	Risk - II	Cumulative Reward	Risk - I	Reward - II	Risk - II	Cumulative Reward	Risk - I
Setting I	0.02	BR-DTRs	1.449(0.086)^1^	1.410(0.072)	2.306(0.077)	1.400(0.053)	1.438(0.093)	1.402(0.076)	2.301(0.078)	1.399(0.054)
	0.02	Naive	—	—	2.224(0.072)	1.377(0.062)	—	—	2.201(0.093)	1.363(0.077)
	0.04	BR-DTRs	1.441(0.083)	1.404(0.067)	2.279(0.071)	1.384(0.051)	1.437(0.094)	1.402(0.077)	2.281(0.077)	1.383(0.053)
	0.04	Naive	—	—	2.207(0.086)	1.359(0.064)	—	—	2.196(0.085)	1.355(0.071)
	0.06	BR-DTRs	1.442(0.089)	1.405(0.074)	2.276(0.071)	1.377(0.050)	1.435(0.092)	1.400(0.075)	2.268(0.070)	1.376(0.050)
	0.06	Naive	—	—	2.185(0.086)	1.348(0.063)	—	—	2.181(0.082)	1.347(0.064)
	0.08	BR-DTRs	1.431(0.086)	1.393(0.070)	2.249(0.078)	1.358(0.048)	1.437(0.093)	1.401(0.076)	2.257(0.068)	1.363(0.045)
	0.08	Naive	—	—	2.164(0.088)	1.322(0.066)	—	—	2.168(0.082)	1.335(0.062)
	0.1	BR-DTRs	1.428(0.081)	1.394(0.066)	2.237(0.066)	1.357(0.045)	1.430(0.094)	1.396(0.077)	2.239(0.065)	1.350(0.044)
	0.1	Naive	—	—	2.168(0.074)	1.321(0.051)	—	—	2.161(0.073)	1.327(0.057)
		AOWL	1.983(0.010)	2.149(0.044)	3.257(0.018)	2.678(0.096)	1.914(0.030)	2.099(0.083)	3.212(0.036)	2.584(0.218)
Setting II	0.02	BR-DTRs	1.362(0.173)	1.246(0.247)	3.174(0.283)	1.262(0.198)	1.456(0.106)	1.403(0.157)	3.069(0.192)	1.329(0.076)
	0.02	Naive	—	—	1.797(0.000)	0.166(0.000)	—	—	1.816(0.019)	0.184(0.018)
	0.04	BR-DTRs	1.306(0.202)	1.166(0.288)	3.228(0.188)	1.299(0.130)	1.459(0.102)	1.402(0.153)	3.066(0.196)	1.319(0.080)
	0.04	Naive	—	—	1.797(0.000)	0.166(0.000)	—	—	1.797(0.000)	0.166(0.000)
	0.06	BR-DTRs	1.238(0.252)	1.067(0.371)	3.221(0.197)	1.297(0.126)	1.444(0.123)	1.377(0.189)	3.068(0.208)	1.311(0.086)
	0.06	Naive	—	—	1.797(0.000)	0.166(0.000)	—	—	1.797(0.000)	0.166(0.000)
	0.08	BR-DTRs	1.228(0.329)	1.059(0.479)	3.178(0.229)	1.260(0.149)	1.430(0.129)	1.360(0.195)	3.049(0.204)	1.297(0.078)
	0.08	Naive	—	—	1.797(0.000)	0.166(0.000)	—	—	1.797(0.000)	0.166(0.000)
	0.1	BR-DTRs	1.177(0.404)	0.980(0.576)	3.169(0.239)	1.247(0.152)	1.438(0.123)	1.371(0.179)	3.009(0.206)	1.271(0.094)
	0.1	Naive	—	—	1.797(0.000)	0.166(0.000)	—	—	1.797(0.001)	0.167(0.001)
		AOWL	2.440(0.064)	3.017(0.002)	5.188(0.000)	2.839(0.000)	2.424(0.080)	3.018(0.002)	5.188(0.000)	2.839(0.000)

1. The estimated results are reported in *median(dev)* format. *median* denotes the median of expected risk/reward estimated via normalized estimator of 600 repeated analyses. *dev* denotes the median value of the absolute difference between estimated risk/reward and *median*

**Table 2:** Estimated reward/risk under different risk constraints for DURABLE study analysis . Results are reported in the same format as Table 1.

Risk Constraint		BR-DTRs			Naive		
$\tau_{2}$	$\tau_{1}$	Reward	Stage II Risk	Stage I Risk	Reward	Stage II Risk	Stage I Risk
0.334	0.893	1.471(0.072)	0.311(0.033)	0.844(0.044)	1.460(0.087)	0.311(0.033)	0.842(0.049)
	0.948	1.520(0.078)	0.311(0.033)	0.874(0.067)	1.499(0.091)	0.311(0.033)	0.868(0.066)
	1.005	1.547(0.089)	0.311(0.033)	0.929(0.102)	1.527(0.098)	0.311(0.033)	0.923(0.111)
∞	0.893	1.598(0.043)	0.347(0.028)	0.832(0.039)	1.604(0.048)	0.347(0.028)	0.840(0.040)
	0.948	1.605(0.053)	0.347(0.028)	0.832(0.040)	1.607(0.056)	0.347(0.028)	0.850(0.056)
	1.005	1.620(0.068)	0.347(0.028)	0.922(0.107)	1.625(0.062)	0.347(0.028)	0.888(0.103)
	∞	1.713(0.052)	0.347(0.025)	1.040(0.047)	-	-	-

**Figure S.1:**
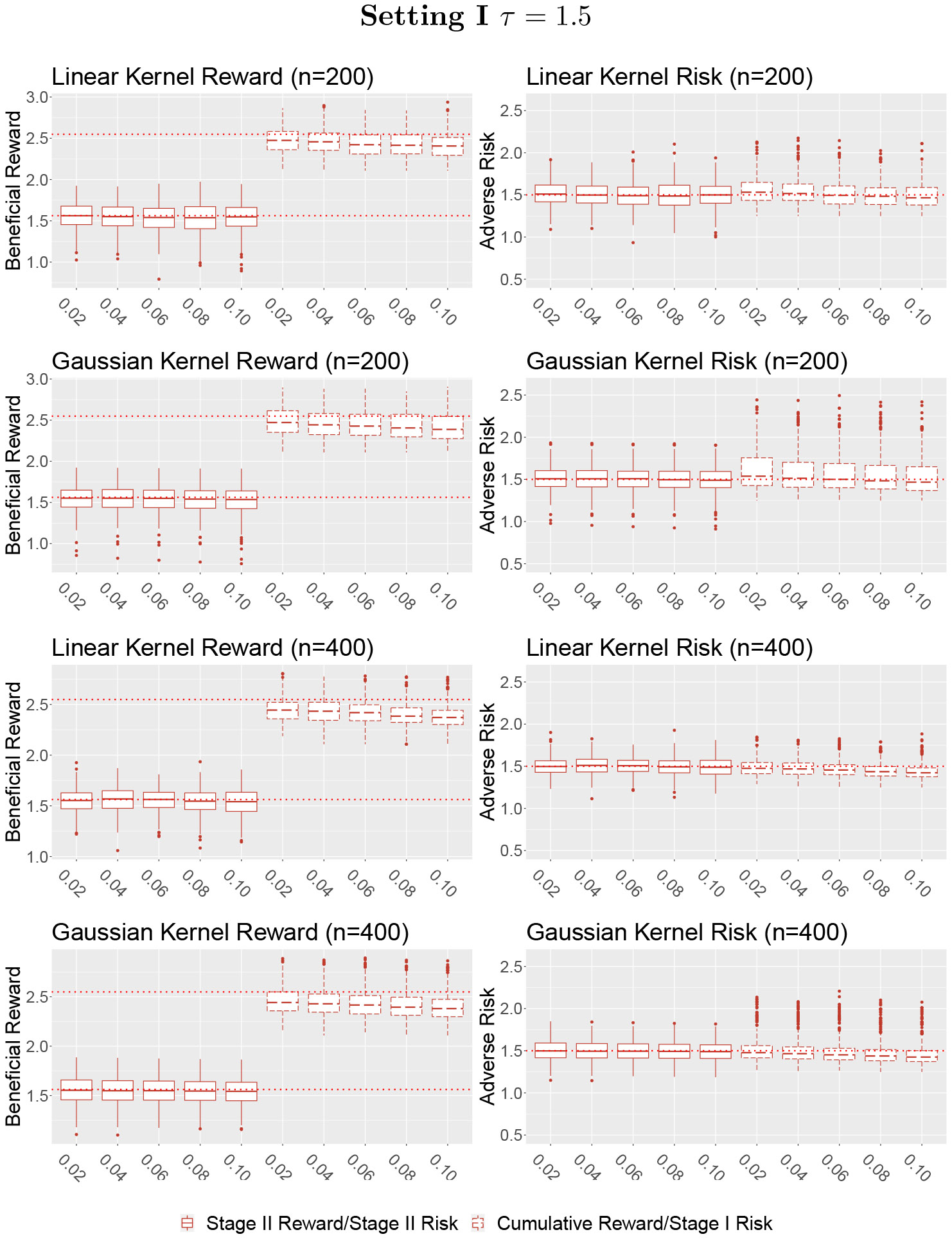
Estimated reward/risk on independent testing data set for simulation setting I, training sample size n={200,400}, η={0.02,0.04,…,0.1} (x-axis) under linear kernel or Gaussian kernel. The dashed line in reward plots refers to the theoretical optimal reward under given constraints. The dashed line in risk plots represents the risk constraint τ=1.5.

**Figure S.2:**
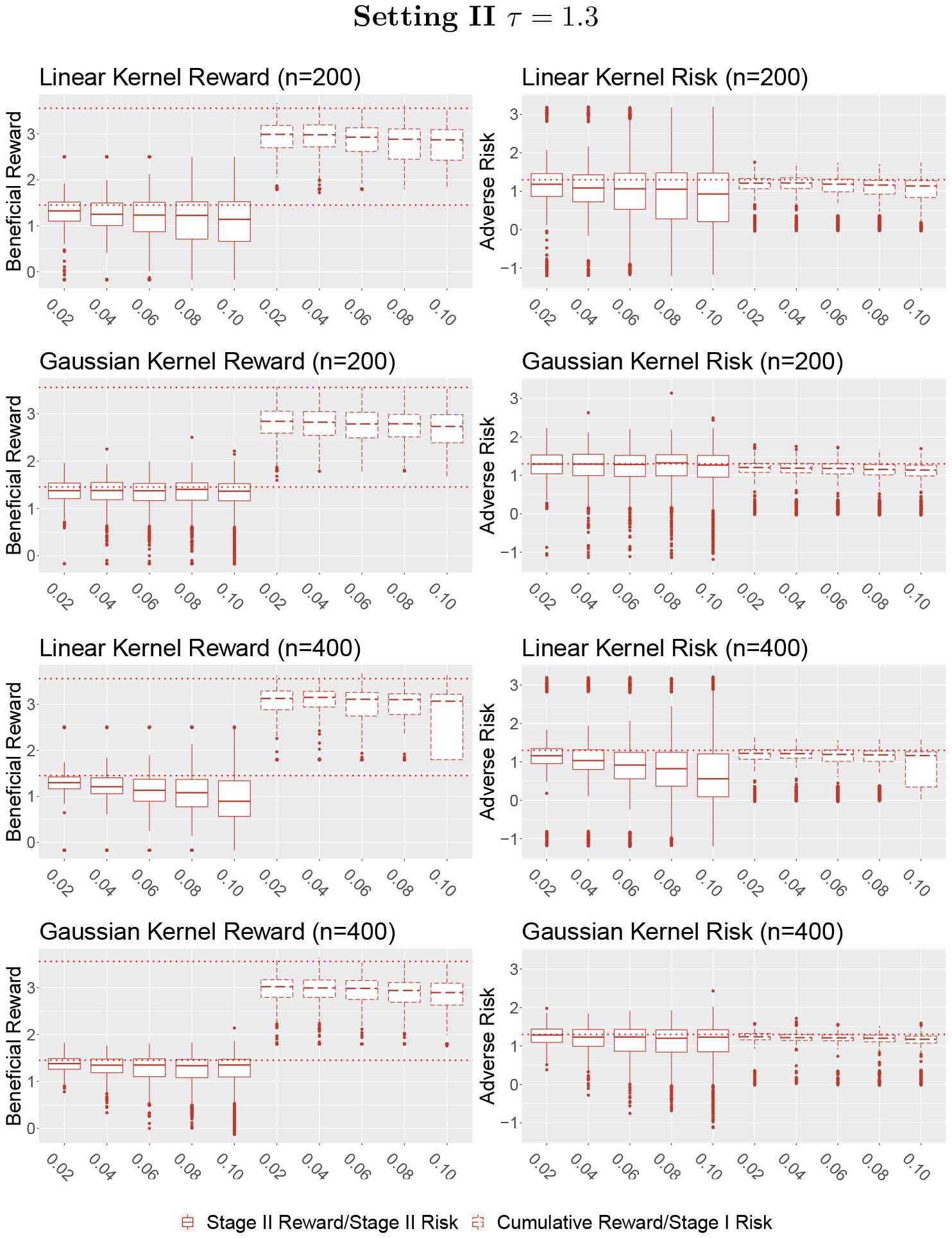
Estimated reward/risk on independent testing data set for simulation setting II, training sample size n={200,400}, η={0.02,0.04,…,0.1} (x-axis) under linear kernel or Gaussian kernel. The dashed line in reward plots refers to the theoretical optimal reward under given constraints. The dashed line in risk plots represents the risk constraint τ=1.3.

**Figure S.3:**
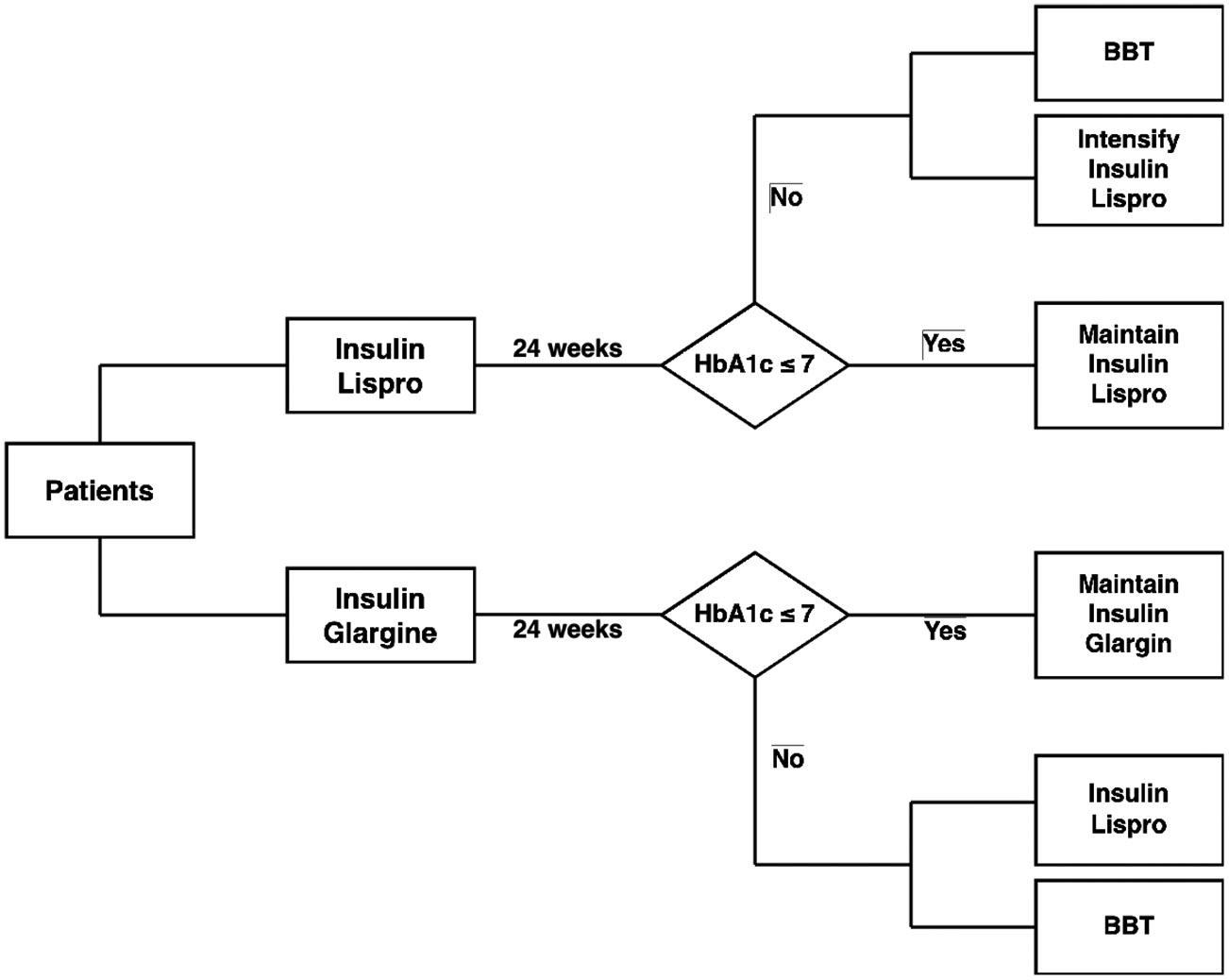
Study design of the DURABLE trial.

**Figure S.4:**
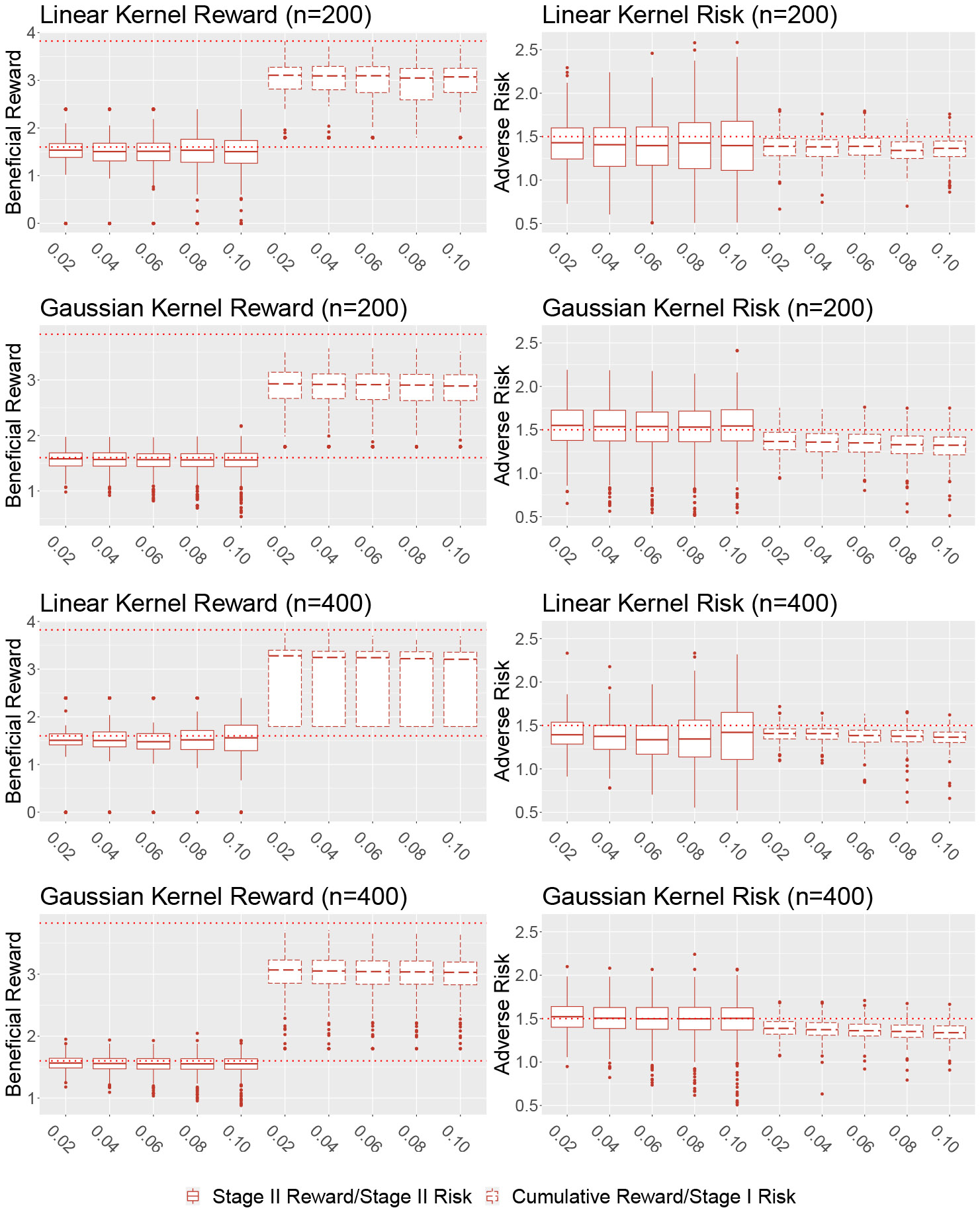
Estimated reward/risk on independent testing data set for the additional simulation study with an unknown and unbalanced design. Results are reported in the way as Figures 1 and 2.

## Appendix A: Proof of Theorem 2 and Theorem 5

We summarize the additional notations used in the proofs below:

$$f_{t}^{*}\;\;\;\;\;\;\;\text{the true optimal decision function solving the BR-DTRs}\;(4),\;\;\text{and we use}\;f_{t,\tau }^{*}\;\text{for}\;f_{t}^{*}\;\text{whenever}\;\tau \;\text{is necessary in the context};\;\begin{array}{cc} {𝒱}_{t,\phi }(s,h) & -\{\phi (s)E[Q_{t}|H_{t}=h,A_{t}=1]+\phi (-s)E[Q_{t}|H_{t}=h,A_{t}=-1]\};\\ {ℛ}_{t,\psi }(s,\eta ,h) & \psi (s,\eta )E[R_{t}|H_{t}=h,A_{t}=1]+\psi (-s,\eta )E[R_{t}|H_{t}=h,A_{t}=-1]. \end{array}$$

When T=1, we omit subscript t from all these notations.

### Appendix A.1. Proof of Theorem 2

#### A.1.1 Verification of equation (1)

In this section, we provide detailed intermediate steps for establishing (1). In other word, we show that equations (i)−(iv) hold

$$E^{𝒟}[R_{t}]\overset{\left(i\right)}{=}E\left[\frac{R_{t}\prod_{t=1}^{T}I(A_{t}f_{t}(H_{t})\;>\;0)}{\prod_{t=1}^{T}p(A_{t}|H_{t})}\right]\overset{\left(ii\right)}{=}E\left[R_{t}(\text{sign}(f_{1}),\ldots .,\text{sign}(f_{t}))\right]\;\overset{\left(iii\right)}{=}E\left[R_{t}(\text{sign}(f_{t}))\right]\overset{\left(iv\right)}{=}E\left[\frac{R_{t}I(A_{t}f_{t}(H_{t})\;>\;0)}{p(A_{t}|H_{t})}\right].$$

The proof uses the fact from Qian and Murphy (2011) and Zhao et al. (2015), which shows that under Assumptions 1 to 3, we have

(9)
$$\frac{dP_{𝒟}}{dP}=\frac{\prod_{s=1}^{T}I(A_{s}f_{s}(H_{s})\;>\;0)}{\prod_{s=1}^{T}p(A_{s}|H_{s})},$$

and

(10)
$$\frac{dP_{\left({𝒟}_{1},\ldots ,{𝒟}_{t}\right)}}{dP}=\frac{\prod_{s=1}^{t}I(A_{s}f_{s}(H_{s})\;>\;0)}{\prod_{s=1}^{t}p(A_{s}|H_{s})},\;\;\frac{dP_{{𝒟}_{t}}}{dP}=\frac{I(A_{t}f_{t}(H_{t})\;>\;0)}{p(A_{t}|H_{t})}.$$

Equality (i) immediately follows by (9). To show (ii), we first note that by conditioning on ($H_{T}$, $A_{T}$), we have that for any t<T

E[Rt∏t=1TI(Atft(Ht)>0)∏t=1Tp(At∣Ht)]=E[E[∣Rt∏t=1TI(Atft(Ht)>0)∏t=1Tp(At∣Ht)∣HT,AT]]=E[Rt∏t=1T−1I(Atft(Ht)>0)∏t=1T−1p(At∣Ht)[E[I(ATfT(HT)>0)p(AT∣HT)∣HT,AT]]=E[Rt∏t=1T−1I(Atft(Ht)>0)∏t=1T−1p(At∣Ht)].

Repeating the same argument from stage T−1 to t, we have

$$E^{𝒟}[R_{t}]=E\left[\frac{R_{t}\prod_{s=1}^{t}I(A_{s}f_{s}(H_{s})\;>\;0)}{\prod_{s=1}^{t}p(A_{s}|H_{s})}\right].$$

Then, equations (ii) and (iv) can be obtained by applying (10) and recalling that by definition $E^{\left({𝒟}_{1},\ldots ,{𝒟}_{t}\right)}[R_{t}]=E[R_{t}(\text{sign}(f_{1}),\ldots ,\text{sign}(f_{t}))]$ and $E^{{𝒟}_{t}}[R_{t}]=E[R_{t}(\text{sign}(f_{t}))]$. Lastly, equation (iii) is followed by Assumption 4 which implies that $R_{t}({\overset{‒}{a}}_{t})\;=\;R_{t}(a_{t})$ for any ${\overset{‒}{a}}_{t}\in {\left\{-1,+1\right\}}^{t}$ and, consequently, $R_{t}(\text{sign}(f_{1}),\ldots ,\text{sign}(f_{t}))=R_{t}(\text{sign}(f_{t}))$.

#### A.1.2 Proof of Theorem 2 for T=1

We consider T=1. After dropping the stage subscript, both (2) and (3) are equivalent to solving

(11)
$$\underset{f\in ℱ}{\text{min}}\;E\left[\frac{YI(Af(H)\;<\;0)}{p(A|H)}\right],\;\;\text{subject to}\;E\left[\frac{RI(Af(H)\;>\;0)}{p(A|H)}\right]\leq \tau ,$$

and its resulting decision is given by $\text{sign}(g^{*})$. Without loss of generality, we assume that Y is nonnegative; otherwise, we can change Y to ∣Y∣ and A to A∗sign(Y), which will not change the optimal solution since the objective functions are equivalent up to a constant due to

$$E\left[\frac{YI(Af(H)\;<\;0)}{p(A|H)}\right]=E\left[\frac{Y^{+}I(Af(H)\;<\;0)}{p(A|H)}\right]-E\left[\frac{Y^{-}I(Af(H)\;<\;0)}{p(A|H)}\right]\;\;=E\left[\frac{Y^{+}I(Af(H)\;<\;0)}{p(A|H)}\right]+E\left[\frac{Y^{-}I(Af(H)\;>\;0)}{p(A|H)}\right]-E\left[\frac{Y^{-}}{p(A|H)}\right]\;\;=E\left[\frac{|Y|I(A*\text{sign}(Y)f(H)\;<\;0)}{p(A|H)}\right]-E\left[\frac{Y^{-}}{p(A|H)}\right],$$

and note that $E[Y^{-}∕p(A|H)]$ is a term that is independent of f. In addition, following the notation in Section 2.2, given random variables (Y, R, A, H) and for a=±1, we define

$$m_{Y}(h,a)=E[Y|H=h,A=a],\;\;\;\delta_{Y}(h)=m_{Y}(h,1)-m_{Y}(h,-1),\;m_{R}(h,a)=E[R|H=h,A=a],\;\;\;\delta_{R}(h)=m_{R}(h,1)-m_{R}(h,-1),$$

and let

$$\tau_{\text{min}}=E\left[R\frac{I(A\delta_{R}(H)<0)}{p(A|H)}\right],\;\tau_{\max}=E\left[R\frac{I(A\delta_{Y}(H)>0)}{p(A|H)}\right].$$

We define $ℳ\;=\;\{h\;:\delta_{Y}(h)\delta_{R}(h)<0\}$, i.e., the set of subjects where the beneficial treatment also reduces risk. Then according to Theorem 1 in Wang et al. (2018), for any $\tau \in (\tau_{\text{min}},\tau_{\max})$, the optimal $g^{*}$ can be chosen as

g∗(h)={sign(δY(h)),ifh∈ℳ1,ifh∈{δY(h)∕δR(h)>λ∗,δY(h)>0}∩ℳc−1,ifh∈{δY(h)∕δR(h)<λ∗,δY(h)>0}∩ℳc−1,ifh∈{δY(h)∕δR(h)>λ∗,δY(h)<0}∩ℳc1,ifh∈{δY(h)∕δR(h)<λ∗,δY(h)<0}∩ℳc,}

where $\lambda^{*}$ satisfies $E[RI\left(Ag^{*}(H)>0\right)∕p(A|H)]=\tau$. Our surrogate problem to be solved is (4), which is

(12)
$$\underset{f\in ℱ}{\text{min}}E\left[\frac{Y\phi (Af(H))}{p(A|H)}\right],\;\text{subject to}\;E\left[\frac{R\psi (Af(H),\eta )}{p(A|H)}\right]\leq \tau .$$

We let $f^{*}$ denote the solution. Our following theorem (the same version for Theorem 2 for T=1) gives an explicit expression for $f^{*}$ so that the solution for the surrogate problem has the same sign as $g^{*}$.

**Theorem 8** For any fixed $\tau_{\text{min}}<\tau <\tau_{\max}$, suppose that $P(\delta_{Y}(H)\delta_{R}(H)=0)=0$ and random variable $\delta_{Y}(H)∕\delta_{R}(H)$ has distribution function with a continuous density function in the support of H. Then for any η∈(0,1], $f^{*}(h)$ can be taken as

(13)
f∗(h)={sign(δY(h)),ifh∈ℳ1,ifh∈{δY(h)∕δR(h)>λ∗,δY(h)>0}∩ℳc−η,ifh∈{δY(h)∕δR(h)<λ∗,δY(h)>0}∩ℳc−1,ifh∈{δY(h)∕δR(h)>λ∗,δY(h)<0}∩ℳcη,ifh∈{δY(h)∕δR(h)<λ∗,δY(h)<0}∩ℳc,}

where $\lambda^{*}$ is the same one in the definition of $g^{*}$

By comparing the expressions for $g^{*}$ and $f^{*}$, we immediately conclude that they have the same signs so solving (12) leads to a Fisher consistent solution to the original problem in (11). The proof consists of several steps. For any decision function f, we say that f is feasible meaning that f satisfies the risk constraint in the surrogate problem (12), and for any two feasible functions, $f_{1}$ and $f_{2}$, “$f_{1}$ is non-inferior to $f_{2}$” means that the objective function in (12) is less than or equal to the one for $f_{2}$, and “$f_{1}$ is superior to $f_{2}$” if the objective function is strictly less than.

From now on, we assume η∈(0,1] and $\tau \in (\tau_{\text{min}},\tau_{\max})$. By the definitions of ${𝒱}_{\phi }$ and ${ℛ}_{\psi }$, we note

$$\;E\left[\frac{Y\phi (Af(H))}{p(A|H)}\right]=-E[{𝒱}_{\phi }(f,H)],\;E\left[\frac{R\psi (Af(H),\eta )}{p(A|H)}\right]=E[{ℛ}_{\psi }(f,\eta ,H)].$$

Proof of Theorem 8:

Step 1. We show that the value for the optimal solution, $f^{*}$, can be restricted within [−1, 1]. That is, the following lemma holds.

**Lemma 9** For any feasible decisi function f(h), define $\tilde{f}(h)=\text{min}(\max(f(h),-1),1)$ as the truncated f at −1 and 1. Then $\tilde{f}$ is non-inferior to f.

**Proof** Note that ψ(h,η)=ψ(1,η) for any h>1 and ψ(h,η)=ψ(−1,η) for any h<−1. Thus, it follows from η≤1 that $E[{ℛ}_{\psi }(\tilde{f},\eta ,H)]=E[{ℛ}_{\psi }(f,\eta ,H)]\leq \tau$, so $\tilde{f}$ is feasible. Moreover, it is easy to see that if f(h)>1, then $\tilde{f}(h)=1$ so

E[∣Yϕ(Af(H))p(A∣X)∣H=h]=E[Y∣A=−1,H=h](1+f(h))≥2E[Y∣A=−1,H=h]=E[∣Yϕ(Af~(H))p(A∣X)∣H=h].

Similarly, if f(h)<−1,

E[∣Yϕ(Af(H))p(A∣X)∣H=h]=E[Y∣A=1,H=h](1−f(h))≥2E[Y∣A=−1,H=h]=E[∣Yϕ(Af~(H))p(A∣X)∣H=h].

Since $f(h)=\tilde{f}(h)$ when ∣f(h)∣≤1, we conclude

$$E\left[\frac{Y\phi (Af(H))}{p(A|X)}\right]\geq E\left[\frac{Y\phi (A\tilde{f}(H))}{p(A|X)}\right].$$

Thus, Lemma 9 holds. ■

Step 2. We characterize the expression of $f^{*}(h)$ for h∈ℳ, which is the region where the beneficial treatment also reduces the risk.

**Lemma 10** For any feasible function f with ∣f∣≤1, we define

$$\tilde{f}(h)=f(h)I(h\in {ℳ}^{c})+sign(\delta_{Y}(h))I(h\in ℳ).$$

Then $\tilde{f}$ is non-inferior to f.

**Proof** For any h∈ℳ with $\delta_{Y}(h)>0$ and $\delta_{R}(h)<0$, ${ℛ}_{\psi }(s,\eta ,h)$ is minimized when s∈[η,1], while ${𝒱}_{\phi }(s,h)$ is maximized at s=1. Since $\tilde{f}(h)=1$ for any h∈ℳ, we have ${ℛ}_{\psi }(\tilde{f}(h),\eta ,h)\leq {ℛ}_{\psi }(f(h),\eta ,h)$ and ${𝒱}_{\phi }(\tilde{f}(h),h)\geq {𝒱}_{\phi }(f(h),h)$. The same inequalities hold for h with $\delta_{Y}(h)<0$ and $\delta_{R}(h)>0$. In other words, they hold for any h∈ℳ.

Since $\tilde{f}(h)=f(h)$ for $h\in {ℳ}^{c}$,

$$E[{ℛ}_{\psi }(f,\eta ,H)]-E[{ℛ}_{\psi }(\tilde{f},\eta ,H)]=E[({ℛ}_{\psi }(f,\eta ,H)-{ℛ}_{\psi }(\tilde{f},\eta ,H))I(H\in ℳ)]\geq 0,$$

and similarly, $E[{𝒱}_{\phi }(f,H)]-E[{𝒱}_{\phi }(\tilde{f},H)]\leq 0$. We conclude that $\tilde{f}$ is non-inferior to f. ■

Step 3. From steps 1 and 2, we can restrict f to satisfy ∣f∣≤1 and $f(h)=\text{sign}(\delta_{Y}(h))$ for h∈ℳ. Furthermore, since $\tau_{\max}$ is the risk under decision rule $\text{sign}(\delta_{Y}(h)),\tau <\tau_{\max}$ implies that

$$P(f(H)\neq \text{sign}(\delta_{Y}(H)),H\in {ℳ}^{c})>0.$$

In this step, we wish to show that the optimal solution should attain the risk bound, i.e., $E[{ℛ}_{\psi }(f,\eta ,H)]=\tau$. Otherwise, assume for some feasible solution f such that $E[{ℛ}_{\psi }(f,\eta ,H)]=\tau_{0}<\tau$. Consider two sets

$$\;{𝒟}^{+}=\{h\in ℋ\;:f(h)<1,\delta_{Y}(h)>0\}\cap {ℳ}^{c}\;{𝒟}^{-}=\{h\in ℋ\;:f(h)>-1,\delta_{Y}(h)<0\}\cap {ℳ}^{c},$$

then $P({𝒟}^{+})+P({𝒟}^{-})>0$. Without loss of generality, we assume that $P({𝒟}^{+})>0$. We construct

f~(h)={f(h),ifh∉𝒟+min(f(h)+η(τ−τ0)MP(𝒟+),1),ifh∈𝒟+,}

where M is the bound for R.

For $h\in {𝒟}^{+}$, ${𝒱}_{\phi }(\tilde{f}(h),h)>{𝒱}_{\phi }(f(h),h)$ since $1\geq \tilde{f}(h)\;>\;f(h)$ and ${𝒱}_{\phi }(s,h)$ is an strictly increasing function of s∈[−1,1] due to $\delta_{Y}(h)>0$. We immediately conclude $E[{𝒱}_{\phi }(\tilde{f},H)]>E[{𝒱}_{\phi }(f,H)]$. On the other hand, ${ℛ}_{\psi }(s,\eta ,h)$ is a piecewise linear function of s with absolute value of slopes no larger than

$$\frac{\max(E[R|H=h,A=1],E[R|H=h,A=-1])}{\eta }\leq \frac{M}{\eta }.$$

Hence, it follows that

$$E[{ℛ}_{\psi }(\tilde{f},\eta ,H)]=E[{ℛ}_{\psi }(\tilde{f},\eta ,H)]-E[{ℛ}_{\psi }(f,\eta ,H)]+E[{ℛ}_{\psi }(f,\eta ,H)]\;\;\leq E[({ℛ}_{\psi }(\tilde{f},\eta ,H)-{ℛ}_{\psi }(f,\eta ,H))I(H\in {𝒟}^{+})]+\tau_{0}\;\;\leq \frac{M}{\eta }\frac{\eta (\tau -\tau_{0})}{MP({𝒟}^{+})}P({𝒟}^{+})+\tau_{0}=\tau .$$

As a result, $\tilde{f}$ is superior to f with a strictly larger objective function, a contradiction. In other words, the expected risk for the optimal solution should attain the bound.

With steps 1-3, we can restrict within the class

$$𝒲=\{f\;:|f|\leq 1,f(h)=\text{sign}(\delta_{Y}(h))\;\text{for}\;h\in ℳ,E[{ℛ}_{\psi }(f,\eta ,H)]=\tau \}$$

to find the optimal decision function.

Step 4. We derive the expression of the optimal function for f by considering solving a Lagrange multiplier for the problem (12):

(14)
$$\max_{f\in 𝒲}-E\left[\frac{Y\phi (Af(H))}{p(A|H)}\right]-\nu \left(E\left[\frac{R\psi (Af(H))}{p(A|H)}\right]-\tau \right),$$

where ν is a constant to be determined by the constraint τ in 𝒲. We maximize the above function by maximizing the conditional mean of the term in the expectation given H=h for every h, which is given by

$$G(f)\equiv {𝒱}_{\phi }(f,h)-\nu R_{\psi }(f,\eta ,h).$$

Note that G(f) is now a function w.r.t. the value of f given fixed h. Since f∈[−1,1] and f in 𝒲 is already given for h∈ℳ, it suffices to examine that for $h\in {ℳ}^{c}$. In addition, G(f) is a piecewise linear function for f∈[−1,−η], (−η,0], (0,η] and (η,1]. Thus, the maximizer can only be achieved at points −1, −η, 0, η and 1. Note that R is assumed to be positive, $G^{\prime }(0)=-\nu ∕\eta (E[R|H=h,A=1]+E[R|H=h,A=-1])<0$ if ν>0, or > 0 if ν<0. For ν=0, G(0)=−E[Y∣H=h,A=1]−E[Y∣H=h,A=−1]=(G(1)+G(−1))∕2. Thus, the maximum for G(f) can always be attained at f which is not zero. In other words, we only need to compare the values at f∈{−1,−η,η,1}.

Simple calculation gives

G(−1)=−2E[Y∣H=h,A=1]−νE[R∣H=h,A=−1],G(−η)=−(1+η)E[Y∣H=h,A=1]−(1−η)E[Y∣H=h,A=−1]−νE[R∣H=h,A=−1],G(η)=−(1−η)E[Y∣H=h,A=1]−(1+η)E[Y∣H=h,A=−1]−νE[R∣H=h,A=1],

and

G(1)=−2E[Y∣H=h,A=−1]−νE[R∣H=h,A=1].

When $\delta_{Y}(h)>0$ so $\delta_{R}(h)$ is also positive, it is straightforward to check G(1)>G(η) and G(−η)>G(−1). Note $G(1)-G(-\eta )=(1+\eta )\delta_{Y}(h)-\lambda \delta_{R}(h)$ so we immediately conclude that the optimal value for f should be 1, if $\delta_{Y}(h)>\lambda$, where λ=ν∕(1+η), and it is −η otherwise. When $\delta_{Y}(h)\leq 0$, we use the same arguments to obtain that the optimal value for f should be −1 if $\delta_{Y}(h)>\lambda$, and it is η otherwise. Therefore, the optimal function maximizing the Lagrange multiplier for any fixed ν (equivalently, λ) has the same expression as (13).

Next, we show that there is some positive $\lambda^{*}=\nu^{*}∕(1+\eta )$ such that

$$E[RI\left(Ag^{*}(H)>0\right)∕p(A|H)]=E[RI\left(Af^{*}(H)>0\right)∕p(A|H)]=E[{ℛ}_{\psi }(f^{*},\eta ,H)]=\tau .$$

The first equality follows from the fact that $\text{sign}(g^{*})=\text{sign}(f^{*})$, and the second equality follows from that $R_{\psi }(s,\eta ,h)$ is constant for any s∈[−1,−η] and s∈[η,1]. To prove the existence of $\lambda^{*}$, we notice

(15)
$$\Gamma (\lambda )\equiv E[RI\left(Af^{*}(H)>0\right)∕p(A|H)]\;\;=E[E[R|H,A=1]I(H\in \{\delta_{Y}(h)>0\}\cap ℳ)]\;\;+E[E[R|H,A=-1]I(H\in \delta_{Y}(h)<0\}\cap ℳ)]\;\;+E[E[R|H,A=1]I(H\in \{\delta_{Y}(h)∕\delta_{R}(h)>\lambda ,\delta_{Y}(h)>0\}\;\cap \;{ℳ}^{c})]\;\;+E[E[R|H,A=-1]I(H\in \{\delta_{Y}(h)∕\delta_{R}(h)<\lambda ,\delta_{Y}(h)>0\}\;\cap \;{ℳ}^{c})]\;\;+E[E[R|H,A=-1]I(H\in \{\delta_{Y}(h)∕\delta_{R}(h)>\lambda ,\delta_{Y}(h)<0\}\;\cap \;{ℳ}^{c})]\;\;+E[E[R|H,A=1]I(H\in \{\delta_{Y}(h)∕\delta_{R}(h)<\lambda ,\delta_{Y}(h)<0\}\;\cap \;{ℳ}^{c})]$$

is a continuous function of λ since $\delta_{Y}(H)∕\delta_{R}(H)$ has a continuous density function. Furthermore, $\Gamma (\infty )=\tau_{\text{min}}$, $\Gamma (0)=\tau_{\max}$. Thus, there exists some $\lambda^{*}>0$ such that $\Gamma (\lambda^{*})=\tau$. Finally, for any f, based on steps 1-3, we have

$$-E\left[\frac{Y\phi (Af(H))}{p(A|H)}\right]\leq \max_{f\in 𝒲}\left\{-E\left[\frac{Y\phi (Af(H))}{p(A|H)}\right]\right\}.$$

On the other hand, for any f∈𝒲, we have E[Rψ(Af(H))∕p(A∣H)]=τ and

$$\;-E\left[\frac{Y\phi (Af(H))}{p(A|H)}\right]-\nu^{*}\left(E\left[\frac{R\psi (Af(H))}{p(A|H)}\right]-\tau \right)\;\leq -E\left[\frac{Y\phi (Af^{*}(H))}{p(A|H)}\right]-\nu^{*}\left(E\left[\frac{R\psi (Af^{*}(H))}{p(A|H)}\right]-\tau \right).$$

The inequality above holds since $f^{*}$ maximizes the Lagrange function (14) under multiplier $\nu^{*}$ for any f∈𝒲. Therefore, for any f we have

$$E\left[\frac{Y\phi (Af(H))}{p(A|H)}\right]\geq E\left[\frac{Y\phi (Af^{*}(H))}{p(A|H)}\right].$$

In other words, $f^{*}$ given by (13) is the optimal solution to the problem (12). We thus complete the proof of Theorem 8.

#### A.1.3 Proof of Theorem 2 for T≥2

Start from stage T. For any given $f_{1},\ldots ,f_{T-1}$, we consider $f_{T}$ maximizing

$$E\left[\frac{\left(\sum_{t=1}^{T}Y_{t})I(A_{T}f_{T}(H_{T})\;>\;0\right)}{p(A_{T}|H_{T})}\frac{\prod_{t=1}^{T-1}I(A_{t}f_{t}(H_{t})\;>\;0)}{\prod_{t=1}^{T-1}p(A_{t}|H_{t})}\right]$$

subject to constraint

$$E\left[\frac{R_{T}I(A_{T}f_{T}(H_{T})\;>\;0)}{p(A_{T}|H_{T})}\frac{\prod_{t=1}^{T-1}I(A_{t}f_{t}(H_{t})\;>\;0)}{\prod_{t=1}^{T-1}p(A_{t}|H_{t})}\right]\leq \tau_{T}.$$

Based on Theorem 1 in Wang et al. (2018), the optimal solution can be chosen as

g~T∗(h)={sign(δY~(h)),ifh∈ℳ~1,ifh∈{δY~(h)∕δR~(h)>λ~,δY~(h)>0}∩ℳ~c−1,ifh∈{δY~(h)∕δR~(h)<λ~,δY~(h)>0}∩ℳ~c−1,ifh∈{δY~(h)∕δR~(h)>λ~,δY~(h)<0}∩ℳ~c1,ifh∈{δY~(h)∕δR~(h)<λ~,δY~(h)<0}∩ℳ~c,}

where

$$\delta_{\tilde{Y}}(h)=(E[Q_{T}|H_{T}=h,A_{T}=1]-E[Q_{T}|H_{T}=h,A_{T}=-1])\frac{\prod_{t=1}^{T-1}I(A_{t}f_{t}(H_{t})\;>\;0)}{\prod_{t=1}^{T-1}p(A_{t}|H_{t})},\;\delta_{\tilde{R}}(h)=(E[R_{T}|H_{T}=h,A_{T}=1]-E[R_{T}|H_{T}=h,A_{T}=-1])\frac{\prod_{t=1}^{T-1}I(A_{t}f_{t}(H_{t})\;>\;0)}{\prod_{t=1}^{T-1}p(A_{t}|H_{t})},$$

$\tilde{ℳ}=\{h\;:\delta_{\tilde{Y}}(h)\delta_{\tilde{R}}(h)<0\}$, and and $\tilde{\lambda }$ satisfies

$$E\left[\frac{R_{T}I(A_{T}{\tilde{g}}_{T}^{*}(H_{T})\;>\;0)}{p(A_{T}|H_{T})}\frac{\prod_{t=1}^{T-1}I(A_{t}f_{t}(H_{t})\;>\;0)}{\prod_{t=1}^{T-1}p(A_{t}|H_{t})}\right]=\tau_{T}.$$

Note that for h in the support of $H_{t}$ where $A_{t}f_{t}(H_{t})\leq 0$ for any t=1,…,T−1, ${\tilde{g}}_{T}^{*}(h)$ can be any arbitrary value since it does not affect the value and risk expectations. On the other hand, recall that $g_{T}^{*}(h)$ is the function maximizing

$$E\left[\frac{\left(\sum_{t=1}^{T}Y_{T})I(A_{T}f_{T}(H_{T})\;>\;0\right)}{p(A_{T}|H_{T})}\right]$$

subject to constraint

$$E\left[\frac{R_{T}I(A_{T}f_{T}(H_{T})\;>\;0)}{p(A_{T}|H_{T})}\right]\leq \tau_{T}.$$

Based on Theorem 1 in Wang et al. (2018), $g_{T}^{*}$ is given as

gT∗(h)={sign(δQT(h)),ifh∈ℳ1,ifh∈{δQT(h)∕δRT(h)>λ∗,δQT(h)>0}∩ℳc−1,ifh∈{δQT(h)∕δRT(h)<λ∗,δQT(h)>0}∩ℳc−1,ifh∈{δQT(h)∕δRT(h)>λ∗,δQT(h)<0}∩ℳc1,ifh∈{δQT(h)∕δRT(h)<λ∗,δQT(h)<0}∩ℳc,}

where $ℳ=\{h\;:\delta_{Q_{T}}(h)\delta_{R_{T}}(h)<0\}$, and $\lambda^{*}$ satisfies

$$E\left[\frac{R_{T}I(A_{T}{\tilde{g}}_{T}^{*}(H_{T})\;>\;0)}{p(A_{T}|H_{T})}\frac{\prod_{t=1}^{T-1}I(A_{t}f_{t}(H_{t})\;>\;0)}{\prod_{t=1}^{T-1}p(A_{t}|H_{t})}\right]=\tau_{T}.$$

From the above two expressions, it is clear that on the set when $A_{t}f_{t}(H_{t})\;>\;0$ for all t=1,…,T−1, ${\tilde{g}}_{T}^{*}(h)$ takes the same form as the solution as $g_{T}^{*}(h)$. Furthermore, due to the conclusion in Section A.1.1, we have

$$E\left[\frac{R_{T}I(A_{T}f_{T}(H_{T})\;>\;0)}{p(A_{T}|H_{T})}\frac{\prod_{t=1}^{T-1}I(A_{t}f_{t}(H_{t})\;>\;0)}{\prod_{t=1}^{T-1}p(A_{t}|H_{t})}\right]=E\left[\frac{R_{T}I(A_{T}f_{T}(H_{T})\;>\;0)}{p(A_{T}|H_{T})}\right].$$

Thus, we conclude that $\tilde{\lambda }$ can be chosen to be the same as $\lambda^{*}$ so ${\tilde{g}}_{T}^{*}(h)$ can be chosen to be exactly the same as $g_{T}^{*}(h)$. In other words,

$$𝒱(f_{1},\ldots ,f_{T-1},g_{T}^{*})\geq 𝒱(f_{1},\ldots ,f_{T})$$

and $g_{T}^{*}$ satisfies

$$E\left[\frac{R_{T}I(A_{T}g_{T}^{*}(H_{T})\;>\;0)}{p(A_{T}|H_{T})}\frac{\prod_{t=1}^{T-1}I(A_{t}f_{t}(H_{t})\;>\;0)}{\prod_{t=1}^{T-1}p(A_{t}|H_{t})}\right]=\tau_{T}.$$

By Theorem 8, both $g_{T}^{*}$ and $f_{T}^{*}$ have the same signs. Therefore,

$$𝒱(f_{1},\ldots ,f_{T-1},f_{T}^{*})\geq 𝒱(f_{1},\ldots ,f_{T})$$

and $f_{T}^{*}$ satisfies

$$E\left[\frac{R_{T}I(A_{T}f_{T}^{*}(H_{T})\;>\;0)}{p(A_{T}|H_{T})}\right]=\tau_{T}.$$

Once $f_{T}^{*}$ is determined, we consider the T−1 stage. Now the original problem (2) becomes

$$\max\;𝒱(f_{1},\ldots ,f_{T-1},f_{T}^{*})=E\left[\frac{\left(\sum_{t=1}^{T}Y_{t})I(A_{T}f_{T}^{*}(H_{T})\;>\;0\right)}{p(A_{T}|H_{T})}\frac{\prod_{t=1}^{T-1}I(A_{t}f_{t}(H_{t})>0)}{\prod_{t=1}^{T-1}p(A_{t}|H_{t})}\right]\;\;\text{subject to}\;\;\;\;\;\;\;\;\;E\left[\frac{R_{t}I(A_{t}f_{t}(H_{t})>0)}{p(A_{t}|H_{t})}\right]\leq \tau_{t},\;\;\;\;\;\;\;\;\;\;t=1,\ldots ,T-1.$$

We repeat the same arguments as for stage T as before, to conclude

$$𝒱(f_{1},\ldots ,f_{T-1}^{*},f_{T}^{*})\geq 𝒱(f_{1},\ldots ,f_{T-1},f_{T})$$

and $f_{T-1}^{*}$ satisfies

$$E\left[\frac{R_{T-1}I(A_{T-1}f_{T-1}^{*}(H_{T-1})>0)}{p(A_{T-1}|H_{T-1})}\right]=\tau_{T-1}.$$

We continue this proof till t=1 so conclude that ($f_{1}^{*},\ldots ,f_{T}^{*}$) maximizes the multistage value and satisfies the constraints over all the stages. The above arguments also show that $f_{t}^{*}$ has the same sign as $g_{t}^{*}$. Theorem 2 thus holds.

### Appendix A.2. Proof of Theorem 5

Instead, we prove a more general version of Theorem 5.

**Theorem 11** In addition to the conditions in Theorem 2, suppose that Assumption 5 holds and $H_{t}$ takes value from a compact subset of $R^{d_{t}}$ for t=1,…,T. Let ($\tau_{1},\ldots ,\tau_{T}$) and ($\delta_{0,1},\ldots ,\delta_{0,T}$) denote the constraints and corresponding constants in Assumption 5. Let $\delta_{t}>0$, $1\leq x_{t}$, $0<\theta_{1,t}$, $0<\theta_{2,t}$, $0<\nu_{1,t}<2$, $0<\nu_{2,t}<2$ for t=1,…,T. Give positive parameter $\lambda_{n,t}\to 0$ and $\sigma_{n,t}\to \infty$ and let

ξn,t(1)=c(2Mc1Mc1λn,t+σn,tdt)+(λn,t(Mc1λn,t+σn,tdt))n−1∕2(σn,t(1−ν1,t∕2)(1+θ1,t)dt∕2+22xt+2xt∕n),

$\xi_{n,t}^{\left(2\right)}=c(\lambda_{n,t}\sigma_{n,t}^{d_{t}}+\sigma_{n,t}^{-\alpha_{t}d_{t}})$ and $\xi_{n,t}=\xi_{n,t}^{\left(1\right)}+\xi_{n,t}^{\left(2\right)}$. In addition, let

$$\epsilon_{n,t}^{\prime }=\delta_{t}+C_{1,t}\sigma_{n,t}^{-\alpha_{t}d_{t}}\eta_{n,t}^{-1}+C_{3,t}n^{-1∕2}\sigma_{n,t}^{(1-\nu_{2,t}∕2)(1+\theta_{2,t})d_{t}∕2}{\left(\frac{M}{c_{1}\lambda_{n,t}}+\sigma_{n,t}^{d_{t}}\right)}^{\nu_{2,t}∕4}\eta_{n,t}^{-\nu_{2,t}∕2}$$

and

$$h_{t}(n,x_{t})=2\;\text{exp}\left(-\frac{2n\delta_{0,t}^{2}c_{1}^{2}}{M^{2}}\right)+2\;\text{exp}\left(-\frac{n\delta_{t}^{2}c_{1}^{2}}{2M^{2}}\right)+\text{exp}(-x_{t}).$$

Then for any n≥1 and ($\lambda_{n,t},\sigma_{n,t},\eta_{n,t}$) such that

$$\;C_{1,t}\sigma_{n,t}^{-\alpha_{t}d_{t}}\eta_{n,t}^{-1}\leq \delta_{0,t},\;C_{2,t}\sigma_{n,t}^{(1-\nu_{1,t}∕2)(1+\theta_{1,t})d_{t}}\leq 1,$$

$\epsilon_{n,t}^{\prime }<2\delta_{0,t}$, and $x_{t}\geq 1$, with probability at least $1-\sum_{t=1}^{T}h_{t}(n,x_{t})$, we have

(16)
$$|𝒱({\hat{f}}_{1},\ldots ,{\hat{f}}_{T})-𝒱(g_{1}^{*},\ldots ,g_{T}^{*})|\leq \sum_{t=1}^{T}{\left(c_{1}∕5\right)}^{1-t}(\xi_{n,t}+(T-t+1)M\eta_{n,t}+2c\epsilon_{n,t}^{\prime }).$$

Moreover, with probability at least $1-h_{t}(n,x_{t})$ the risk induced by ${\hat{f}}_{t}$ satisfies

(17)
$$E\left[\frac{R_{t}I(A_{t}{\hat{f}}_{t}(H_{t})>0)}{p(A_{t}|H_{t})}\right]\leq \tau_{t}+\delta_{t}+C_{3,t}\sigma_{n,t}^{(1-\nu_{2,t}∕2)(1+\theta_{2,t})d_{t}∕2}{\left(\frac{M}{c_{1}\lambda_{n,t}}+\sigma_{n,t}^{d_{t}}\right)}^{\nu_{2,t}∕4}\eta_{n,t}^{-\nu_{2,t}∕2}.$$

Here, c in front of $\xi_{n,t}^{\left(1\right)}$ is a positive constant only depends on ($\nu_{1,t}$, $\theta_{1,t}$, $d_{t}$), c in front of $\xi_{n,t}^{\left(2\right)}$ is a positive constant only depends on ($\alpha_{t}$, $d_{t}$, $K_{t}$, M) and c of $\epsilon_{n,t}^{\prime }$ is a positive constant only depends on ($\tau_{t}$, $\delta_{0,t}$). $C_{1,t}$ is a positive constant depend on ($\alpha_{t}$, $d_{t}$, $K_{t}$, M), $C_{2,t}$ is a positive constant depends on ($\nu_{1,t}$, $\theta_{1,t}$, $d_{t}$), $C_{3,t}$ a positive constant depends on ($\nu_{2,t}$, $\theta_{2,t}$, $d_{t}$, $c_{1}$, M).

Theorem 5 can be obtained from Theorem 11 by setting $\theta_{t}=\theta_{1,t}=\theta_{2,t}$, $\nu_{t}=\nu_{1,t}=\nu_{2,t}$, $x_{t}=\sigma_{n,t}^{(1-\nu_{t}∕2)(1+\theta_{t})d_{t}}$, and $C_{i}={\text{sup}}_{t}C_{i,t}$ for i=1, 2, 3. We first prove Theorem 11 by for T=1 and then extend the result to T≥2.

#### A.2.1 Proof of Theorem 11 for T=1

Since T=1, we omit the subscript for the stage in this subsection, so all the notations are the same as in Section A.1.2. Since τ is necessary for the proof, we use $f_{\tau }^{*}$ to refer to $f^{*}$ that solves (12) corresponding to τ and shifting parameter $\eta_{n}$.

##### A.2.1.1 Excessive risk

In this section, we prove some preliminary lemmas. Lemma 12 shows that the regret from the optimal decision function solving the original problem (11) is bounded by the regret from the one solving the surrogate problem (12), plus an additional biased term of order $O(\eta_{n})$. Lemma 13 shows that the optimal value using the surrogate loss is Lipschitz continuous with respect to τ.

**Lemma 12** Under the condition of Theorem 8, for any f:ℋ→R and any $\eta_{n}\in (0,1]$, we have

$$𝒱(f_{\tau }^{*})-𝒱(f)\leq E[{𝒱}_{\phi }(f_{\tau }^{*},H)]-E[{𝒱}_{\phi }(f,H)]+M\eta_{n}.$$

**Proof** Theorem 8 shows that $f_{\tau }^{*}$ must have expression (13) almost surely. Let $\tilde{𝒱}(f,h)=I(f(h)>0)E[Y|H=h,A=1]+I(f(h)\leq 0)E[Y|H=h,A=-1]$. For any $h\in \{\delta_{Y}(h)>0\}$, we consider the following 6 scenarios:

1. When h∈ℳ, $f_{\tau }^{*}(h)=1$ and f(h)>0, we have $\tilde{𝒱}(f_{\tau }^{*},h)-\tilde{𝒱}(f,h)=0$ and
  𝒱ϕ(fτ∗,h)−𝒱ϕ(f,h)={(1−f(h))δY(h),f(h)≤1(f(h)−1)mY(h,−1),f(h)>1,}
  which implies ${𝒱}_{\phi }(f_{\tau }^{*},h)-{𝒱}_{\phi }(f,h)\geq \tilde{𝒱}(f_{\tau }^{*},h)-\tilde{𝒱}(f,h)$.
2. When h∈ℳ, $f_{\tau }^{*}(h)=1$ and f(h)≤0, we have $\tilde{𝒱}(f_{\tau }^{*},h)-\tilde{𝒱}(f,h)=\delta_{Y}(h)$ and
  𝒱ϕ(fτ∗,h)−𝒱ϕ(f,h)={(1−f(h))δY(h),f(h)≥−12δY(h)+(−f(h)−1)mY(h,1),f(h)<−1,}
  which implies ${𝒱}_{\phi }(f_{\tau }^{*},h)-{𝒱}_{\phi }(f,h)\geq \tilde{𝒱}(f_{\tau }^{*},h)-\tilde{𝒱}(f,h)$.
3. When $h\in {ℳ}^{c}$, $f_{\tau }^{*}(h)=1$ and f(h)>0, we have $\tilde{𝒱}(f_{\tau }^{*},h)-\tilde{𝒱}(f,h)=0$ and
  𝒱ϕ(fτ∗,h)−𝒱ϕ(f,h)={(1−f(h))δY(h),f(h)≤1(f(h)−1)mY(h,−1),f(h)>1,}
  in which case ${𝒱}_{\phi }(f_{\tau }^{*},h)-{𝒱}_{\phi }(f,h)\geq \tilde{𝒱}(f_{\tau }^{*},h)-\tilde{𝒱}(f,h)$.
4. When $h\in {ℳ}^{c}$, $f_{\tau }^{*}(h)=1$ and f(h)≤0, we have $\tilde{𝒱}(f_{\tau }^{*},h)-\tilde{𝒱}(f,h)=\delta_{Y}(h)$ and
  𝒱ϕ(fτ∗,h)−𝒱ϕ(f,h)={(1−f(h))δY(h),f(h)≥−12δY(h)+(−f(h)−1)mY(h,1),f(h)<−1,}
  in which case ${𝒱}_{\phi }(f_{\tau }^{*},h)-{𝒱}_{\phi }(f,h)\geq \tilde{𝒱}(f_{\tau }^{*},h)=\tilde{𝒱}(f,h)$.
5. When $h\in {ℳ}^{c}$, $f_{\tau }^{*}(h)=-\eta_{n}$ and f(h)>0, we have $\tilde{𝒱}(f_{\tau }^{*},h)-\tilde{𝒱}(f,h)=-\delta_{Y}(h)$ and
  𝒱ϕ(fτ∗,h)−𝒱ϕ(f,h)={−f(h)δY(h)−ηnδY(h),f(h)≤−1−δY(h)−ηnδY(h)+(f(h)−1)mY(h,−1),f(h)>−1.}
  Thus, ${𝒱}_{\phi }(f_{\tau }^{*},h)-{𝒱}_{\phi }(f,h)\geq -\delta_{Y}(h)-\eta_{n}\delta_{Y}(h)=\tilde{𝒱}(f_{\tau }^{*},h)-\tilde{𝒱}(f,h)-\eta_{n}\delta_{Y}(h)$.
6. When $h\in {ℳ}^{c},f_{\tau }^{*}(h)=-\eta_{n}$ and f(h)≤0, we have $\tilde{𝒱}(f_{\tau }^{*},h)-\tilde{𝒱}(f,h)=0$ and
  𝒱ϕ(fτ∗,h)−𝒱ϕ(f,h)={−f(h)δY(h)−ηnδY(h),f(h)≥−1(f(h)−1)mY(h,1)+(1−ηn)δY(h),f(h)<−1.}
  Thus, ${𝒱}_{\phi }(f_{\tau }^{*},h)-{𝒱}_{\phi }(f,h)\geq -\eta_{n}\delta_{Y}(h)=\tilde{𝒱}(f_{\tau }^{*},h)-\tilde{𝒱}(f,h)-\eta_{n}\delta_{Y}(h)$.

Hence, by combining all these cases, we conclude that

$$\tilde{𝒱}(f_{\tau }^{*},h)-\tilde{𝒱}(f,h)\leq {𝒱}_{\phi }(f_{\tau }^{*},h)-{𝒱}_{\phi }(f,h)+Mn\eta_{n}$$

for any $\eta_{n}\in (0,1]$ and any decision function f. The same argument holds for any h such that $\delta_{Y}(h)<0$. Consequently, since $𝒱(f)=E[\tilde{𝒱}(f,H)]$, we have

$$𝒱(f_{\tau }^{*})-𝒱(f)\leq E[{𝒱}_{\phi }(f_{\tau }^{*},H)]-E[{𝒱}_{\phi }(f,H)]+M\eta_{n}.$$

■

**Lemma 13** For any δ>0 and τ such that $\left[\tau -2\delta ,\tau +2\delta ]\subseteq (\tau_{\min},\tau_{\max}\right)$, $E[{𝒱}_{\phi }(f_{\tau }^{*},H)]$, as a function of τ, is Lipschitz continuous at τ.

**Proof** Let $\tau_{1}=\tau$ and $\tau_{2}$ be any number in [τ−2δ, τ+2δ]. Without loss of generality, we assume $\tau_{2}<\tau_{1}$. We also let $f_{1}^{*}$ and $f_{2}^{*}$ be the optimal decision functions solving (12) for $\tau_{1}$ and $\tau_{2}$, respectively, and their corresponding $\lambda^{*}$’s values are denoted as $\lambda_{1}$ and $\lambda_{2}$. According to (13), it is easy to verify that

$$\;E[{𝒱}_{\phi }(f_{1}^{*},H)]-E[{𝒱}_{\phi }(f_{2}^{*},H)]\;=E\left[\left(1+\eta_{n})\delta_{Y}(H)I\left(\lambda_{1}\leq \frac{\delta_{Y}(H)}{\delta_{R}(H)}\leq \lambda_{2}\right)I(\delta_{Y}(H)>0)I(H\in {ℳ}^{c}\right)\right]\;\;-E\left[\left(1+\eta_{n})\delta_{Y}(H)I\left(\lambda_{1}\leq \frac{\delta_{Y}(H)}{\delta_{R}(H)}\leq \lambda_{2}\right)I(\delta_{Y}(H)<0)I(H\in {ℳ}^{c}\right)\right]\;=(1+\eta_{n})E\left[|\delta_{Y}(H)|I\left(\lambda_{1}\leq \frac{\delta_{Y}(H)}{\delta_{R}(H)}\leq \lambda_{2}\right)I(H\in {ℳ}^{c})\right].$$

On the other hand

$$\tau_{1}-\tau_{2}=E[{ℛ}_{\psi }(f_{1}^{*},\eta_{n},H)I(H\in {ℳ}^{c})]-E[{ℛ}_{\psi }(f_{2}^{*},\eta_{n},H)I(H\in {ℳ}^{c}]\;\;=E\left[\delta_{R}(H)I\left(\lambda_{1}\leq \frac{\delta_{Y}(H)}{\delta_{R}(H)}\leq \lambda_{2}\right)I(\delta_{Y}(H)>0)I(H\in {ℳ}^{c})\right]\;\;-E\left[\delta_{R}(H)I\left(\lambda_{1}\leq \frac{\delta_{Y}(H)}{\delta_{R}(H)}\leq \lambda_{2}\right)I(\delta_{Y}(H)<0)I(H\in {ℳ}^{c})\right]\;\;=E\left[|\delta_{R}(H)|I\left(\lambda_{1}\leq \frac{\delta_{Y}(H)}{\delta_{R}(H)}\leq \lambda_{2}\right)I(H\in {ℳ}^{c})\right].$$

The above two equations imply that

$$\;E[{𝒱}_{\phi }(f_{1}^{*},H)]-E[{𝒱}_{\phi }(f_{2}^{*},H)]\;=(1+\eta_{n})E\left[\frac{|\delta_{Y}(H)|}{|\delta_{R}(H)|}|\delta_{R}(H)|I\left(\lambda_{1}\leq \frac{\delta_{Y}(H)}{\delta_{R}(H)}\leq \lambda_{2}\right)I(H\in {ℳ}^{c})\right]\;\leq 2\lambda_{2}E\left[|\delta_{R}(H)|I\left(\lambda_{1}\leq \frac{\delta_{Y}(H)}{\delta_{R}(H)}\leq \lambda_{2}\right)I(H\in {ℳ}^{c})\right]\;\leq 2\lambda_{2}(\tau_{1}-\tau_{2}).$$

The lemma holds since $\lambda_{2}$ is no larger than $\lambda^{*}$-value corresponding to τ−2δ. ■

##### A.2.1.2 Approximation error in RKHS

In this section, we prove a series of lemmas to quantify the approximation error of $\hat{f}$, where $\hat{f}$ denotes the solution of the single-stage empirical problem

(18)
$$\;\underset{f\in 𝒢}{\text{arg}\;\text{min}}\frac{1}{n}\sum_{i=1}^{n}Y_{i}\frac{\phi (A_{i}f(H_{i}))}{p(A_{i}|H_{i})}+\lambda_{n}{\left\|f\right\|}_{𝒢}^{2}\;\text{subject to}\;\frac{1}{n}\sum_{i=1}^{n}R_{i}\frac{\psi (A_{i}f(H_{i}),\eta_{n})}{p(A_{i}|H_{i})}\leq \tau ,$$

resulted from restricting $\hat{f}$ to be a function in Gaussian RKHS 𝒢.

The section is organized as follows: Lemma 14 provides an approximation of $f_{\tau }^{*}$ using functions in Gaussian RKHS; in Lemma 16, we quantify the difference of risk under shifted ramp loss between $f_{\tau }^{*}$ and its approximation in 𝒢; in Lemma 17, we show that ${\left\|\hat{f}\right\|}_{𝒢}$ is bounded with high probability; in Lemma 18, we show that ${𝒜}_{n}(\tau )$ defined later changes continuously w.r.t. τ and the approximation error is given Lemma 20.

For convenience, we define

$$\;L_{\phi }(f)=Y\frac{\phi (Af(H))}{p(A|H)},\;\;P_{n}[L_{\phi }(f)]=\frac{1}{n}\sum_{i=1}^{n}Y_{i}\frac{\phi (A_{i}f(H_{i}))}{p(A_{i}|H_{i})},\;R_{\psi }(f,\eta_{n})=R\frac{\psi (Af(H),\eta_{n})}{p(A|H)},\;\;P_{n}[R_{\psi }(f,\eta_{n})]=\frac{1}{n}\sum_{i=1}^{n}R_{i}\frac{\psi (A_{i}f(H_{i}),\eta_{n})}{p(A_{i}|H_{i})},$$

where $P_{n}$ denotes the empirical distribution. Recall $𝒢=𝒢(\sigma_{n})$ denote the Gaussian Reproducing Kernel Hilbert Space (RKHS) with bandwidth $\sigma_{n}^{-1}$, we define

𝒜(τ)={f∈𝒢∣E[Rψ(f,ηn)]≤τ}},𝒜n(τ)={f∈𝒢∣Pn[Rψ(f,ηn)]≤τ}},

where ${𝒜}_{n}(\tau )$ is equivalent to the definition of the feasible region of the empirical problem with T=1. We also define $\bar{ℋ}=3ℋ$,

δ¯Y(h)={δY(h),if∣h∣≤1δY(h∕∣h∣),if∣h∣>1,}δ¯R(h)={δR(h),if∣h∣≤1δR(h∕∣h∣),if∣h∣>1.}

Following the notation in Assumption 5 and omitting the index t when T=1, we define

$${\bar{H}}_{a,b,\tau }=\left\{h\in \bar{ℋ}\;:a{\bar{\delta }}_{Y}(h)>0,b({\bar{\delta }}_{Y}(h)-\lambda^{*}{\bar{\delta }}_{R}(h))>0\right\}$$

so $\Delta_{\tau }(h)=\sum_{a,b\in \{-1,1\}}\text{dist}(h,\bar{ℋ}∕{\bar{H}}_{a,b,\tau })I(h\in {\bar{H}}_{a,b,\tau })$, where a, b∈{−1,1} and $\lambda^{*}$ is the multiplier associated witn $f_{\tau }^{*}$ so function Δ depends on τ, and

f¯τ(h)={1,ifh∈H¯1,1ηn,ifh∈H¯−1,1−1,ifh∈H¯−1,−1−ηn,ifh∈H¯1,−10,otherwise.}

Thus, ${\bar{f}}_{\tau }$ can be viewed as an extension of $f_{\tau }^{*}$ from of ℋ to $\bar{ℋ}$. Our first lemma is to determine the pointwise approximation error of $f_{\tau }^{*}$ using the RKHS. Note that we assumed ℋ is a compact subset of 𝒢, without loss of generality, from now on we assume that $ℋ\subseteq {ℬ}_{𝒢}$ where ${ℬ}_{𝒢}$ denotes the unit ball in 𝒢. We use d to denote the dimension of ℋ.

**Lemma 14** Let ${\overset{ˇ}{f}}_{\tau }={\left(\sigma_{n}^{2}∕\pi \right)}^{d∕4}{\bar{f}}_{\tau }$ and define linear operator

$$V_{\sigma }\overset{ˇ}{f}(x)=\frac{{\left(2\sigma \right)}^{d∕2}}{\pi^{d∕4}}\int_{R^{d}}e^{-2\sigma^{2}{\left\|x-y\right\|}_{2}^{2}}\overset{ˇ}{f}(y)dy.$$

Then, we have

(19)
$${\left\|V_{\sigma_{n}}{\overset{ˇ}{f}}_{\tau }\right\|}_{𝒢}^{2}\leq c\sigma_{n}^{d},$$

and

(20)
$$|V_{\sigma_{n}}{\overset{ˇ}{f}}_{\tau }(h)-f_{\tau }^{*}(h)|\leq 8e^{-\sigma_{n}^{2}\Delta_{\tau }{\left(h\right)}^{2}∕2d}.$$

holds for all h∈ℋ, where c is a constant depending on dimension d.

**Remark 15** Note that $V_{\sigma }{\overset{ˇ}{f}}_{\tau }$ is an approximation of $f_{\tau }^{*}$ in 𝒢. Thus, Lemma 14 quantifies the distance between the true optimal decision function and its approximation at each point h.

**Proof** Since $ℋ\subset {ℬ}_{𝒢}$ and ${\overset{ˇ}{f}}_{\tau }={\left(\sigma_{n}^{2}∕\pi \right)}^{d∕4}{\bar{f}}_{\tau }$, we can easily obtain that the $L_{2}$ norm of ${\overset{ˇ}{f}}_{\tau }$ satisfies

$${\left\|{\overset{ˇ}{f}}_{\tau }\right\|}_{2}^{2}\leq \text{Vol}{\left(d\right)}^{2}{\left(\frac{81}{\pi }\right)}^{d∕2}\sigma_{n}^{d}=c\sigma_{n}^{d},$$

where Vol(d) is the volume of ${ℬ}_{𝒢}$ (see equation (25) from Steinwart and Scovel (2007)) so c is a positive constant depends only on d. Moreover, it has been shown in Steinwart et al. (2006) that $V_{\sigma }\;:L^{2}(R^{d})\to 𝒢(\sigma )$ is an isometric isomorphism and the inequality above implies

$${\left\|V_{\sigma_{n}}{\overset{ˇ}{f}}_{\tau }\right\|}_{𝒢}^{2}={\left\|{\overset{ˇ}{f}}_{\tau }\right\|}_{2}^{2}\leq c\sigma_{n}^{d}.$$

We now start proving (20). By the construction of ${\bar{f}}_{\tau }$, it is straightforward to see that ${\bar{f}}_{\tau }(h)=f_{\tau }^{*}(h)$ for all h∈ℋ. Note for any $h\in H_{1,1}$ we have

$$V_{\sigma_{n}}{\overset{ˇ}{f}}_{\tau }(h)={\left(\frac{2\sigma_{n}^{2}}{\pi }\right)}^{d∕2}\int_{R^{d}}e^{-2\sigma_{n}^{2}{\left\|h-y\right\|}^{2}}{\bar{f}}_{\tau }(y)dy\;\;={\left(\frac{2\sigma_{n}^{2}}{\pi }\right)}^{d∕2}\left[\int_{B(h,\Delta_{\tau }(h))}e^{-2\sigma_{n}^{2}{\left\|h-y\right\|}^{2}}{\bar{f}}_{\tau }(y)dy+\int_{R^{d}∕B(h,\Delta_{\tau }(h))}e^{-2\sigma_{n}^{2}{\left\|h-y\right\|}^{2}}{\bar{f}}_{\tau }(y)dy\right],$$

where B(h,r) is the ball of radius r centering at h under Euclidean norm. By Lemma 4.1 in Steinwart and Scovel (2007), the construction of ${\bar{f}}_{\tau }$ guarantees that $B(h,\Delta_{\tau }(h))\subseteq {\bar{H}}_{1,1}$ for all $h\in H_{1,1}$. It then follows that for any $h\in H_{1,1}$

$$|V_{\sigma_{n}}{\overset{ˇ}{f}}_{\tau }-f_{\tau }^{*}(h)|=|V_{\sigma_{n}}{\overset{ˇ}{f}}_{\tau }(h)-{\bar{f}}_{\tau }(h)|\;\;=|V_{\sigma_{n}}{\overset{ˇ}{f}}_{\tau }(h)-{\left(\frac{2\sigma_{n}^{2}}{\pi }\right)}^{d∕2}\int_{R^{d}}e^{-2\sigma_{n}^{2}{\left\|h-y\right\|}^{2}}dy|\;\;=|{\left(\frac{2\sigma_{n}^{2}}{\pi }\right)}^{d∕2}\int_{R^{d}∕B(h,\Delta_{\tau }(h))}e^{-2\sigma_{n}^{2}{\left\|h-y\right\|}^{2}}[{\bar{f}}_{\tau }(y)-1]dy|\;\;\leq 2P(|U|\geq \Delta_{\tau }(h)),$$

where the last step uses the fact that ${|{\bar{f}}_{\tau }-1|}_{\infty }\leq 2$, and U follows the spherical Gaussian distribution on $R^{d}$ with parameter $\sigma_{n}$. Following inequality (3.5) from Ledoux and Talagrand (1991), we have

$$P(|U|\geq \Delta_{\tau }(h))\leq 4e^{-\sigma_{n}^{2}\Delta_{\tau }^{2}(h)∕2d}.$$

Similarly, we can obtain the same bound for $h\in {\bar{H}}_{-1,1},{\bar{H}}_{1,-1}$ and $H_{-1,-1}$. As a conclusion, we have

$$|V_{\sigma_{n}}{\overset{ˇ}{f}}_{\tau }(h)-f_{\tau }^{*}(h)|\;\leq 8e^{-\sigma_{n}^{2}\Delta_{\tau }^{2}(h)∕2d}$$

for any h∈ℋ. ■

In the next lemma, we show that under Assumption 5, the difference of the risk under shifted ramp loss between $f_{\tau^{\prime }}^{*}$, and its approximation $V_{\sigma_{n}}{\overset{ˇ}{f}}_{\tau^{\prime }}$ is uniformly bounded by $O(\sigma_{n}^{-\alpha d}\eta_{n}^{-1})$ for any $\tau^{\prime }\in [\tau -2\delta_{0},\tau +2\delta_{0}]$; moreover when n is sufficiently large, $V_{\sigma_{n}}{\overset{ˇ}{f}}_{\tau -2\delta_{0}}$ will belong to the empirical feasible region ${𝒜}_{n}(\tau )$ with high probability when $c\sigma_{n}^{-\alpha d}\eta_{n}^{-1}\leq \delta_{0}$.

**Lemma 16** For any $\tau^{\prime }\in [\tau -2\delta_{0},\tau +2\delta_{0}]$,

(21)
$$|E[R_{\psi }(V_{\sigma_{n}}{\overset{ˇ}{f}}_{\tau^{\prime }},\eta_{n})]-E[R_{\psi }(f_{\tau^{\prime }}^{*},\eta_{n})]|\;\leq c\sigma_{n}^{-\alpha d}\eta_{n}^{-1},$$

where c is a constant depending on (α, d, K, M). Moreover, for any $\sigma_{n}$ and $\eta_{n}$ such that $c\sigma_{n}^{-\alpha d}\eta_{n}^{-1}\leq \delta_{0}$, with probability $1-2\;\text{exp}(\frac{-2n\delta_{0}^{2}c_{1}^{2}}{M^{2}})$, we have $V_{\sigma_{n}}{\overset{ˇ}{f}}_{\tau -2\delta_{0}}\in {𝒜}_{n}(\tau )$.

**Proof** First note that for any measurable function $f_{1}$, $f_{2}\;:ℋ\to R$, we always have

E[Rψ(f1,ηn)]−E[Rψ(f2,ηn)]=E[E[R∣H,A=1][ψ(f1(H),ηn)−ψ(f2(H),ηn)]][+E[R∣H,A=−1][ψ(−f1(H),ηn)−ψ(−f2(H),ηn)]]≤2Mηn−1E[∣f1(H)−f2(H)∣].

Using result (20) in Lemma 14 and Assumption 5, we can obtain

$$|E[R_{\psi }(V_{\sigma_{n}}{\overset{ˇ}{f}}_{\tau^{\prime }},\eta_{n})]-E[R_{\psi }(f_{\tau^{\prime }}^{*},\eta_{n})]|\;\leq \eta_{n}^{-1}16ME[e^{-\sigma_{n}^{2}\Delta_{\tau^{\prime }}{\left(H\right)}^{2}∕2d}]\;\;\leq 16MK{\left(2d\right)}^{\alpha d∕2}\sigma_{n}^{-\alpha d}\eta_{n}^{-1}\;\;=c\sigma_{n}^{-\alpha d}\eta_{n}^{-1}.$$

To prove the remaining part of the lemma, we now let $\tau^{\prime }=\tau -2\delta_{0}$. We note that $R_{\psi }(V_{\sigma_{n}}{\overset{ˇ}{f}}_{\tau^{\prime }},\eta_{n})$ is bounded by $M∕c_{1}$. Based on Hoeffding’s inequality, we can obtain

(22)
$$P\left[|P_{n}[R_{\psi }(V_{\sigma_{n}}{\overset{ˇ}{f}}_{\tau^{\prime }},\eta_{n})]-E[R_{\psi }(V_{\sigma_{n}}{\overset{ˇ}{f}}_{\tau^{\prime }},\eta_{n})]|\geq \delta_{0}\right]\leq 2\;\text{exp}\left(\frac{-2n\delta_{0}^{2}c_{1}^{2}}{M^{2}}\right).$$

According to (21) and under the choice of ($\sigma_{n}$, $\eta_{n}$), we have

(23)
$$\;|E[R_{\psi }(V_{\sigma_{n}}{\overset{ˇ}{f}}_{\tau^{\prime }},\eta_{n})]-(\tau -2\delta_{0})|\;=|E[R_{\psi }(V_{\sigma_{n}}{\overset{ˇ}{f}}_{\tau^{\prime }},\eta_{n})]-E[R_{\psi }(f_{\tau }^{*},\eta_{n})]|\;\leq c\sigma_{n}^{-\alpha d}\eta_{n}^{-1}\leq \delta_{0}.$$

Combining (22) and (23), we obtain

$$P\left[P_{n}[R_{\psi }(V_{\sigma_{n}}{\overset{ˇ}{f}}_{\tau^{\prime }},\eta_{n})]\geq \tau \right]\leq 2\;\text{exp}\left(\frac{-2n\delta_{0}^{2}c_{1}^{2}}{M^{2}}\right),$$

which implies that $V_{\sigma_{n}}{\overset{ˇ}{f}}_{\tau^{\prime }}=V_{\sigma_{n}}{\overset{ˇ}{f}}_{\tau -2\delta_{0}}\in {𝒜}_{n}(\tau )$ with probability at least $1-2\;\text{exp}(\frac{-2n\delta_{0}^{2}c_{1}^{2}}{M^{2}})$. ■

In Lemma 17, we show that ${\left\|\hat{f}\right\|}_{𝒢}$ is bounded with high probability.

**Lemma 17** ${\hat{f}}_{\tau }$ satisfies

(24)
$$P\left({\left\|{\hat{f}}_{\tau }\right\|}_{𝒢}^{2}\leq c\left(\frac{M}{c_{1}\lambda_{1}}+\sigma_{n}^{d}\right)\right)\geq 1-2\;\text{exp}\left(\frac{-2n\delta_{0}^{2}c_{1}^{2}}{M^{2}}\right),$$

for any choice of $c\sigma_{n}^{-\alpha d}\eta_{n}^{-1}\leq \delta_{0}$. Here, the constant c in front of $\sigma_{n}^{d}$ only depends on dimension d and the constant in front of $\sigma_{n}^{-\alpha d}\eta_{n}^{-1}$ is equal to the constant of the same term in Lemma 16.

**Proof** From the last claim of Lemma 16, we have $V_{\sigma_{n}}{\overset{ˇ}{f}}_{\tau -2\delta_{0}}\in {𝒜}_{n}(\tau )$ holds with probability at least $1-2\;\text{exp}(\frac{-2n\delta_{0}^{2}c_{1}^{2}}{M^{2}})$. Using and (19) of Lemma 14, under the choice of ($\sigma_{n}$, $\eta_{n}$) we have

$$\lambda_{n}{\left\|\hat{f}\right\|}_{𝒢}^{2}\leq P_{n}[L_{\phi }(\hat{f})]+\lambda_{n}{\left\|\hat{f}\right\|}_{𝒢}^{2}\leq P_{n}[L_{\phi }(V_{\sigma_{n}}{\overset{ˇ}{f}}_{\tau -2\delta_{0}})]+\lambda_{n}{\left\|V_{\sigma_{n}}{\overset{ˇ}{f}}_{\tau -2\delta_{0}}\right\|}_{𝒢}^{2}\leq c\left(\frac{M}{c_{1}}+\lambda_{1}\sigma_{n}^{d}\right),$$

which gives (24). ■

Lemma 17 implies that, instead of 𝒜(τ) and ${𝒜}_{n}(\tau )$, we can concentrate on the sets given by

𝒜(τ,𝒞n)={f∈𝒢∣‖f‖𝒢≤𝒞n,E[Rψ(f,ηn)]≤τ}},𝒜n(τ,𝒞n)={f∈𝒢∣‖f‖𝒢≤𝒞n,Pn[Rψ(f,ηn)]≤τ}},

where ${𝒞}_{n}=c\sqrt{\frac{M}{c_{1}\lambda_{n}}+\sigma_{n}^{d}}$. This is because $\hat{f}$ belongs to them with a high probability.

We further study the relationships among $𝒜(\tau ,{𝒞}_{n})$ and ${𝒜}_{n}(\tau ,{𝒞}_{n})$. The proof will use a general covering number property for Gaussian RKHS from Steinwart and Scovel (2007), which is stated as Proposition 21 in Section A.2.1.4.

**Lemma 18** For any δ>0 with probability at least $1-\text{exp}(-\frac{n\delta^{2}c_{1}^{2}}{2M^{2}})$, we have

(25)
$$𝒜(\tau -\epsilon_{n},{𝒞}_{n})\subset {𝒜}_{n}(\tau ,{𝒞}_{n})\subset 𝒜(\tau +\epsilon_{n},{𝒞}_{n}),$$

where

$$\epsilon_{n}=c\sigma_{n}^{(1-\nu_{2}∕2)(1+\theta_{2})d∕2}{\left(\frac{M}{c_{1}\lambda_{n}}+\sigma_{n}^{d}\right)}^{\nu_{2}∕4}\eta_{n}^{-\nu_{2}∕2}+\delta$$

for $0<\nu_{2}\leq 2$ and $\theta_{2}>0$. Moreover, let

$$\epsilon_{n}^{\prime }=\epsilon_{n}+c\sigma_{n}^{-\alpha d}\eta_{n}^{-1},$$

then for any $\lambda_{n}$ and $\sigma_{n}$ such that $\epsilon_{n}^{\prime }\leq 2\delta_{0}$, we have $V_{\sigma_{n}}{\overset{ˇ}{f}}_{\tau -\epsilon_{n}^{\prime }}\in 𝒜(\tau -\epsilon_{n},{𝒞}_{n})$ and

$$|E[L_{\phi }(V_{\sigma_{n}}{\overset{ˇ}{f}}_{\tau -\epsilon_{n}^{\prime }})]-E[L_{\phi }(f_{\tau -\epsilon_{n}^{\prime }}^{*})]|\leq c\sigma_{n}^{-\alpha d}.$$

Here, the constants in front of $\sigma_{n}^{-ad}$ and $\sigma_{n}^{-ad}\eta_{n}^{-1}$ are equal to the constants in Lemma 17. c in front of $\epsilon_{n}$ is a constant only dependent on (M, $c_{1}$, $\nu_{2}$, $\theta_{2}$, d).

**Proof** To prove (25), it is sufficient to show that with probability $1-\text{exp}(-\frac{n\delta^{2}c_{1}^{2}}{2M^{2}})$ we have

(26)
$$\underset{f\in R_{\psi ,\eta_{n}}\circ {ℬ}_{𝒢}({𝒞}_{n})}{\text{sup}}|P_{n}[f]-E[f]|\leq \epsilon_{n},$$

where $R_{\psi ,\eta_{n}}\;\circ \;{ℬ}_{𝒢}({𝒞}_{n})=\{R_{\psi }(f,\eta_{n})|f\in {ℬ}_{𝒢}({𝒞}_{n})\}$ and ${ℬ}_{𝒢}({𝒞}_{n})$ denotes the closed ball in 𝒢 with radius ${𝒞}_{n}$. By Theorem 4.10 from Wainwright (2019), we have that

$$\underset{f\in R_{\psi ,\eta_{n}}\circ {ℬ}_{𝒢}({𝒞}_{n})}{\text{sup}}|P_{n}[f]-E[f]|\leq 2{\text{Rad}}_{n}(R_{\psi ,\eta_{n}}\;\circ \;{ℬ}_{𝒢}({𝒞}_{n}))+\delta$$

holds with probability $1-\text{exp}(-\frac{n\delta^{2}c_{1}^{2}}{2M^{2}})$, where ${\text{Rad}}_{n}(ℱ)$ is the Rademacher complexity of some functional set ℱ defined as

$${\text{Rad}}_{n}(ℱ)=E_{X}\left[E_{\epsilon }\left[\underset{f\in ℱ}{\text{sup}}|\frac{1}{n}\sum_{i=1}^{n}\epsilon_{i}f(X_{i})|\right]\right],\;\;\epsilon_{i}∼i.i.d.\;P(\epsilon_{i}=\pm 1)=0.5.$$

Following the proof in Example 5.24 from Wainwright (2019), by Dudley’s entropy integral the Rademacher complexity of $R_{\psi ,\eta_{n}}\;\circ \;{ℬ}_{𝒢}({𝒞}_{n})$ is upper bound by

(27)
$${\text{Rad}}_{n}(R_{\psi ,\eta_{n}}\;\circ \;{ℬ}_{𝒢}({𝒞}_{n}))\leq E\left[\frac{24}{\sqrt{n}}\int_{0}^{2\frac{M}{c_{1}}}\sqrt{\log\;𝒩(R_{\psi ,\eta_{n}}\;\circ \;{ℬ}_{𝒢}({𝒞}_{n}),\epsilon ,L_{2}(P_{n}))}d\epsilon \right]\;\;\overset{\left(i\right)}{\leq }E\left[\frac{24}{\sqrt{n}}\int_{0}^{2\frac{M}{c_{1}}}\sqrt{\log\;𝒩\left({ℬ}_{𝒢},\frac{\eta_{n}c_{1}}{M{𝒞}_{n}}\epsilon ,L_{2}(P_{n})\right)}d\epsilon \right]\;\;\overset{\left(ii\right)}{\leq }c\sigma_{n}^{(1-\nu_{2}∕2)(1+\theta_{2})d∕2}{\left(\frac{M}{c_{1}\lambda_{n}}+c_{d}^{2}\sigma_{n}^{d}\right)}^{\nu_{2}∕4}\eta_{n}^{-\nu_{2}∕2},$$

where to obtain (i) we have used the fact that $R_{\psi ,\eta_{n}}$ is a Lipschitz function of f with Lipschitz constant $\frac{M}{c_{1}\eta_{n}}$, and in (ii) we used the covering number property of ${ℬ}_{𝒢}$ stated in Proposition 21.

For the second part of the lemma, $V_{\sigma_{n}}{\overset{ˇ}{f}}_{\tau -\epsilon_{n}^{\prime }}\in 𝒜(\tau -\epsilon_{n},{𝒞}_{n})$ is a direct conclusion of (21) from Lemma 16 since

$$\;E[R_{\psi }(V_{\sigma_{n}}{\overset{ˇ}{f}}_{\tau -\epsilon_{n}^{\prime }},\eta_{n})]\;\leq |E[R_{\psi }(V_{\sigma_{n}}{\overset{ˇ}{f}}_{\tau -\epsilon_{n}^{\prime }},\eta_{n})]-E[R_{\psi }(f_{\tau -\epsilon_{n}^{\prime }}^{*},\eta_{n})]|+|E[R_{\psi }(f_{\tau -\epsilon_{n}^{\prime }}^{*},\eta_{n})]|\;\leq \tau -\epsilon_{n}^{\prime }+c\sigma_{n}^{-\alpha d}\eta_{n}^{-1}=\tau -\epsilon_{n}.$$

Note that for any $f_{1}$, $f_{2}\;:ℋ\to [-1,1]$ we always have

$$\;E[L_{\phi }(f_{1})]-E[L_{\phi }(f_{2})]\;=E\left[E[Y|H,A=1][\phi (f_{1}(H))-\phi (f_{2}(H))]+E[Y|H,A=-1][\phi (-f_{1}(H))-\phi (-f_{2}(H))]\right]\;\leq E[|\delta_{Y}(H)∥f_{1}(H)-f_{2}(H)|].$$

Hence, using (20) in Lemma 14 and Assumption 5 we have

$$|E[L_{\phi }(V_{\sigma_{n}}{\overset{ˇ}{f}}_{\tau -\epsilon_{n}^{\prime }})]-E[L_{\phi }(f_{\tau -\epsilon_{n}^{\prime }}^{*})]|\leq \;\;8E[|\delta_{Y}(H)|e^{-\sigma_{n}^{2}\Delta_{\tau -\epsilon_{n}^{\prime }}^{2}\left(H\right)∕2d}]\leq 8MK{\left(2d\right)}^{\alpha d∕2}\sigma_{n}^{-\alpha d}=c\sigma_{n}^{-\alpha d}.$$

This completes the proof for the lemma. ■

As a corollary of Lemma 18, we can establish the risk error bound (17) stated in Theorem 11 for T=1. We state this result as Corollary 19 below

**Corollary 19** Suppose ($\sigma_{n}$, $\eta_{n}$) satisfy the requirement in Lemma 17, then for any $0<\nu_{2}\leq 2,\theta_{2}>0$ and δ>0 with probability at least $1-2\;\text{exp}(\frac{-2n\delta_{0}^{2}c_{1}^{2}}{M^{2}})-2\;\text{exp}(-\frac{n\delta^{2}c_{1}^{2}}{2M^{2}})$ we have

$$E\left[\frac{RI(A\hat{f}(H_{t})\;>\;0)}{p(A|H)}\right]\leq \tau +\delta +cn^{-1∕2}\sigma_{n}^{(1-\nu_{2}∕2)(1+\theta_{2})d∕2}{\left(\frac{M}{c_{1}\lambda_{n}}+\sigma_{n}^{d_{t}}\right)}^{\nu_{2}∕4}\eta_{n}^{-\nu 2∕2}.$$

Here, c is a constant only depends on (M, $c_{1}$, $\nu_{2}$, $\theta_{2}$, d).

**Proof** Lemma 17 implies that $\hat{f}$ is bounded by ${𝒞}_{n}$ with probability at least $1-2\;\text{exp}(\frac{-2n\delta_{0}^{2}c_{1}^{2}}{M^{2}})$ Moreover, the concentration inequality (26) of Lemma 18 implies that

$$E[R_{\psi }(f,\eta_{n})]-P_{n}[R_{\psi }(f,\eta_{n})]\leq \delta +cn^{-1∕2}\sigma_{n}^{(1-\nu_{2}∕2)(1+\theta_{2})d∕2}{\left(\frac{M}{c_{1}\lambda_{n}}+\sigma_{n}^{d_{t}}\right)}^{\nu_{2}∕4}\eta_{n}^{-\nu 2∕2}$$

holds with probability at least $1-2\;\text{exp}(-\frac{n\delta^{2}c_{1}^{2}}{2M^{2}})$ for any δ>0 and $f\in {ℬ}_{𝒢}({𝒞}_{n})$. The result holds since $P_{n}[R_{\psi }(\hat{f},\eta_{n})]\leq \tau$ the choice of $\hat{F}$ and note that

$$E\left[\frac{RI(A\hat{f}(H)\;>\;0)}{p(A|H)}\right]\leq E[R_{\psi }(\hat{f},\eta_{n})].$$

■

Lemma 18 indicates that

$$V_{\sigma_{n}}{\overset{ˇ}{f}}_{\tau -\epsilon_{n}^{\prime }}\in 𝒜(\tau -\epsilon_{n},{𝒞}_{n})\subseteq {𝒜}_{n}(\tau ,{𝒞}_{n})$$

with high probability. In Lemma 20, we will show that $V_{\sigma_{n}}{\overset{ˇ}{f}}_{\tau -\epsilon_{n}^{\prime }}$ can be used to quantify the approximation error caused by RKHS.

**Lemma 20** Under the condition of Lemma 18, we have

$$\underset{f\in 𝒜(\tau -\epsilon_{n},{𝒞}_{n})}{\text{inf}}(E[L_{\phi }(f)]+\lambda_{n}{\left\|f\right\|}_{𝒢}^{2}-E[L_{\phi }(f_{\tau -\epsilon_{n}^{\prime }}^{*})])\leq \xi_{n}^{\left(2\right)}.$$

**Proof** Let ${\overset{ˇ}{f}}_{\tau -\epsilon_{n}^{\prime }}={\left(\sigma_{n}^{2}∕\pi \right)}^{d∕4}{\bar{f}}_{\tau -\epsilon_{n}^{\prime }}$, then from Lemma 18 we have

$$|E[L_{\phi }(V_{\sigma_{n}}{\overset{ˇ}{f}}_{\tau -\epsilon_{n}^{\prime }})]-E[L_{\phi }(f_{\tau -\epsilon_{n}^{\prime }}^{*})]|\leq c\sigma_{n}^{-\alpha d},$$

and $V_{\sigma_{n}}{\overset{ˇ}{f}}_{\tau -\epsilon_{n}^{\prime }}\in 𝒜(\tau -\epsilon_{n},{𝒞}_{n})$. Moreover, (19) from Lemma 14 gives that ${\left\|V_{\sigma_{n}}{\overset{ˇ}{f}}_{\tau -\epsilon_{n}^{\prime }}\right\|}_{𝒢}^{2}\leq c\sigma_{n}^{d}$. Hence, we have

$$\;\underset{f\in 𝒜(\tau -\epsilon_{n},{𝒞}_{n})}{\text{inf}}\left[E[L_{\phi }(f)]+\lambda_{n}{\left\|f\right\|}_{𝒢}^{2}-E[L_{\phi }(f_{\tau -\epsilon_{n}^{\prime }}^{*})]\right]\;=\underset{f\in 𝒜(\tau -\epsilon_{n},{𝒞}_{n})}{\text{inf}}\left[E[L_{\phi }(f)]+\lambda_{n}{\left\|f\right\|}_{𝒢}^{2}-E[L_{\phi }(V_{\sigma_{n}}{\overset{ˇ}{f}}_{\tau -\epsilon_{n}^{\prime }})]-\lambda_{n}{\left\|V_{\sigma_{n}}{\overset{ˇ}{f}}_{\tau -\epsilon_{n}^{\prime }}\right\|}_{𝒢}^{2}\right]+\lambda_{n}{\left\|V_{\sigma_{n}}{\overset{ˇ}{f}}_{\tau -\epsilon_{n}^{\prime }}\right\|}_{𝒢}^{2}\;\;+E[L_{\phi }(V_{\sigma_{n}}{\overset{ˇ}{f}}_{\tau -\epsilon_{n}^{\prime }})]-E[L_{\phi }(f_{\tau -\epsilon_{n}^{\prime }}^{*})]\;\leq c(\lambda_{n}\sigma_{n}^{d}+\sigma_{n}^{-\alpha d})\equiv \xi_{n}^{\left(2\right)}.$$

■

##### A.2.1.3 Completing the proof to Theorem 11 for T=1

We first establish the error bound for the regret (16). Since the Fisher consistency of Theorem 8 indicates $𝒱(g^{*})=𝒱(f_{\tau }^{*})$ and using the excessive risk inequality in Lemma 12 we have

$$𝒱(f_{\tau }^{*})-𝒱(\hat{f})\leq E[{𝒱}_{\phi }(f_{\tau }^{*},H)]-E[{𝒱}_{\phi }(\hat{f},H)]+M\eta_{n},$$

the proof is completed if we can show

(28)
$$E[{𝒱}_{\phi }(f_{\tau }^{*},H)]-E[{𝒱}_{\phi }(\hat{f},H)]=E[L_{\phi }(\hat{f})]-E[L_{\phi }(f_{\tau }^{*})]\leq \xi_{n}+2\lambda_{0}\epsilon_{n}^{\prime }=\xi_{n}+c\epsilon_{n}^{\prime }$$

holds with probability at least 1−h(n,x), where $\lambda_{0}$ denotes the $\lambda^{*}$-value for ($\tau -2\delta_{0}$) which is a constant only depends on (τ, $\delta_{0}$).

According to Lemma 17, we have shown that ${\left\|\hat{f}\right\|}_{𝒢}$ is bounded by ${𝒞}_{n}=c\sqrt{\frac{M}{c_{1}\lambda_{n}}+\sigma_{n}^{d}}$ with probability at least $1-2\;\text{exp}(\frac{-2n\delta_{0}^{2}c_{1}^{2}}{M^{2}})$. Hence, similar to proof of Corollary 19, we can restrict to set ${ℬ}_{𝒢}({𝒞}_{n})$, and replace 𝒜(τ) and ${𝒜}_{n}(\tau )$ by $𝒜(\tau ,{𝒞}_{n})$ and ${𝒜}_{n}(\tau ,{𝒞}_{n})$ correspondingly with high probability.

To prove (28), we note that the left-hand side of the inequality can be composed as

(29)
$$\;E[L_{\phi }(\hat{f})]-E[L_{\phi }(f_{\tau }^{*})]\;\leq E[L_{\phi }(\hat{f})]+\lambda_{n}{\left\|\hat{f}\right\|}_{𝒢}^{2}-E[L_{\phi }(f_{\tau }^{*})]\;\leq E[L_{\phi }(\hat{f})]+\lambda_{n}{\left\|\hat{f}\right\|}_{𝒢}^{2}-\underset{f\in {𝒜}_{n}(\tau ,{𝒞}_{n})}{\text{inf}}(E[L_{\phi }(f)]+\lambda_{n}{\left\|f\right\|}_{𝒢}^{2})\;\;+\underset{f\in {𝒜}_{n}(\tau ,{𝒞}_{n})}{\text{inf}}(E[L_{\phi }(f)]+\lambda_{n}{\left\|f\right\|}_{𝒢}^{2})-E[L_{\phi }(f_{\tau }^{*})]\;\leq \underset{\left(I\right)}{\underset{︸}{E[L_{\phi }(\hat{f})]+\lambda_{n}{\left\|\hat{f}\right\|}_{𝒢}^{2}-\underset{f\in {𝒜}_{n}(\tau ,{𝒞}_{n})}{\text{inf}}(E[L_{\phi }(f)]+\lambda_{n}{\left\|f\right\|}_{𝒢}^{2})}}\;\;+\underset{\left(II\right)}{\underset{︸}{\underset{f\in {𝒜}_{n}(\tau ,{𝒞}_{n})}{\text{inf}}(E[L_{\phi }(f)]+\lambda_{n}{\left\|f\right\|}_{𝒢}^{2})-\underset{f\in 𝒜(\tau -\epsilon_{n},{𝒞}_{n})}{\text{inf}}(E[L_{\phi }(f)]+\lambda_{n}{\left\|f\right\|}_{𝒢}^{2})}}\;\;+\underset{\left(III\right)}{\underset{︸}{\underset{f\in 𝒜(\tau -\epsilon_{n},{𝒞}_{n})}{\text{inf}}(E[L_{\phi }(f)]+\lambda_{n}{\left\|f\right\|}_{𝒢}^{2})-E[{ℒ}_{\phi }(f_{\tau -\epsilon_{n}^{\prime }}^{*})]}}+\underset{\left(IV\right)}{\underset{︸}{E[L_{\phi }(f_{\tau -\epsilon_{n}^{\prime }}^{*})]-E[L_{\phi }(f_{\tau }^{*})]}}.$$

Using the inclusion result from Lemma 18, we have

$$𝒜(\tau -\epsilon_{n},{𝒞}_{n})\subseteq {𝒜}_{n}(\tau ,{𝒞}_{n})\subseteq 𝒜(\tau +\epsilon_{n},{𝒞}_{n})$$

holds with probability no more than 2 exp ($-\frac{n\delta_{0}^{2}c_{1}^{2}}{2M^{2}}$), and $𝒜(\tau -\epsilon_{n},{𝒞}_{n})\subseteq {𝒜}_{n}(\tau ,{𝒞}_{n})$ implies (II)<0. Using the approximation error bound obtained in Lemma 20, (III) is bounded by $\xi_{n}^{\left(2\right)}$. For term (IV), using the Lipschitz continuity property of the value function obtained in Lemma 13 we have

$$|E[L_{\phi }(f_{\tau -\epsilon_{n}^{\prime }}^{*})]-E[L_{\phi }(f_{\tau }^{*})]|\leq 2\lambda_{0}\epsilon_{n}^{\prime }.$$

In addition, ${𝒜}_{n}(\tau ,{𝒞}_{n})\subseteq 𝒜(\tau +\epsilon_{n},{𝒞}_{n})$ and Lemma 13 implies that

$$\;E[L_{\phi }(\hat{f})]-E[L_{\phi }(f_{\tau }^{*})]\;\geq E[L_{\phi }(\hat{f})]-E[L_{\phi }(f_{\tau +\epsilon_{n}^{\prime }}^{*})]+E[L_{\phi }(f_{\tau +\epsilon_{n}^{\prime }}^{*})]-E[L_{\phi }(f_{\tau }^{*})]\;\geq E[L_{\phi }(f_{\tau +\epsilon_{n}^{\prime }}^{*})]-E[L_{\phi }(f_{\tau }^{*})]=O(\epsilon_{n}^{\prime }).$$

which provides a lower bound for the difference of the surrogate reward between $\hat{f}$ and $f_{\tau }^{*}$.

Hence, it remains to derive an upper bound for (I). Let

$$𝒲=\{L_{\phi }(f_{1})+\lambda_{n}{\left\|f_{1}\right\|}_{𝒢}^{2}-L_{\phi }(f_{2})-\lambda_{n}{\left\|f_{2}\right\|}_{𝒢}^{2}\;:f_{1}\in {ℬ}_{𝒢}({𝒞}_{n}),f_{2}\in {ℬ}_{𝒢}({𝒞}_{n})\},$$

and note that ${𝒜}_{n}(\tau ,{𝒞}_{n})\subset {ℬ}_{𝒢}({𝒞}_{n})$, then following a similar argument as the proof of Theorem 5.3 of Steinwart and Scovel (2007), it is sufficient to control the probability of

$$P(\exists w\in 𝒲\;\text{with}\;P_{n}(w)\leq 0\;\text{and}\;E(w)\geq \xi_{n}^{1}).$$

To achieve this, we aim at applying Proposition 22 to 𝒲, which requiring that for any w∈𝒲, one has ${\left\|w\right\|}_{\infty }\leq B$ for some constant B>0 and the ϵ-covering number of $𝒩(B^{-1}𝒲,\epsilon ,L_{2}(P_{n}))$ satisfies that

$$\underset{P_{n}}{\text{sup}}\log\;𝒩(B^{-1}𝒲,\epsilon ,L_{2}(P_{n}))\leq l\epsilon^{-p}$$

for some l>0 and p>0.

To verify the first condition, we first note that by the definition of 𝒲, it is sufficient to verify the condition for

$${𝒲}^{\prime }=\{L_{\phi }(f)+\lambda_{n}{\left\|f\right\|}_{𝒢}^{2}\;:f\in {ℬ}_{𝒢}\}({𝒞}_{n}),$$

which will then yield the expected conditions for 𝒲 up to a constant. For any $f\in {ℬ}_{𝒢}({𝒞}_{n})$, we first note that

$${\left\|L_{\phi }(f)+\lambda_{n}{\left\|f\right\|}_{𝒢}^{2}\right\|}_{\infty }\leq \frac{M}{c_{1}}{\left\|f\right\|}_{\infty }+\lambda_{n}{\left\|\tilde{f}\right\|}_{𝒢}^{2}\;\;\leq \frac{cM}{c_{1}}\sqrt{\frac{M}{c_{1}\lambda_{n}}+\sigma_{n}^{d}}+c^{2}\lambda_{n}\left(\frac{M}{c_{1}\lambda_{n}}+\sigma_{n}^{d}\right)=B,$$

since ${\left\|f\right\|}_{\infty }\leq {\left\|f\right\|}_{𝒢}$ for the Gaussian RKHS, which indicates that ${\left\|w\right\|}_{\infty }\leq B$ for any $w\in {𝒲}^{\prime }$. Furthermore, by the sub-additivity of the entropy we have

$$\log\;𝒩(B^{-1}{𝒲}^{\prime },2\epsilon ,L_{2}(P_{n}))\leq \underset{\left(V\right)}{\underset{︸}{\log\;𝒩(B^{-1}\{L_{\phi }(f)\;:f\in {ℬ}_{𝒢}({𝒞}_{n})\},\epsilon ,L_{2}(P_{n}))}}\;\;+\underset{\left(VI\right)}{\underset{︸}{\log\;𝒩(B^{-1}\{\lambda_{n}{\left\|f\right\|}_{𝒢}^{2}\;:f\in {ℬ}_{𝒢}({𝒞}_{n})\},\epsilon ,L_{2}(P_{n}))}}.$$

For (V) we have

(V)≤log𝒩(ℬ𝒢(𝒞n),c1BϵM,L2(Pn))=log𝒩(ℬ𝒢,c1BϵM(cMc1λn+σnd)−1,L2(Pn)))≤log𝒩(ℬ𝒢,cϵ,L2(Pn)),

since $L_{\phi }$ is a 1-Lipschitz function of f and $\frac{c_{1}B}{M}{\left(c\sqrt{\frac{M}{c_{1}\lambda_{n}}+\sigma_{n}^{d}}\right)}^{-1}$ converges to a finite positive constant for sufficient small $\lambda_{n}$ and large $\sigma_{n}$. Moreover, for (VI) we have that for ϵ>0,

$$(VI)\leq \log\left(c\sqrt{\frac{M}{c_{1}\lambda_{n}}+\sigma_{n}^{d}}/(B\epsilon )\right)=\log\left(c\sqrt{\frac{M}{c_{1}\lambda_{n}}+\sigma_{n}^{d}}/B\right)-\log\;\epsilon \leq c(-\log\;\epsilon ).$$

Combining the upper bound of (V) and (VI) and using the covering number property for $\log\;𝒩({ℬ}_{𝒢},\epsilon ,L_{2}(P_{n}))$ given in the Proposition 21, we have

$$\underset{P_{n}}{\text{sup}}\log\;𝒩(B^{-1}𝒲,\epsilon ,L_{2}(P_{n}))\leq \underset{P_{n}}{\text{sup}}\log\;𝒩({ℬ}_{𝒢},c\epsilon ,L_{2}(P_{n}))-\log\;\epsilon \;\;\leq c\sigma_{n}^{(1-\nu_{1}∕2)(1+\theta_{1})d}\epsilon^{-\nu_{1}},$$

for any $0\;<\;\nu_{1}\;<\;2$, $\theta_{1}\;>\;0$, and some positive constant c which only depends on ($\nu_{1}$, $\theta_{1}$, d, M, $c_{1}$). Therefore, let $B=\frac{2cM}{c_{1}}\sqrt{\frac{M}{c_{1}\lambda_{n}}+\sigma_{n}^{d}}+2c^{2}\lambda_{n}\left(\frac{M}{c_{1}\lambda_{n}}+\sigma_{n}^{d}\right)$, $l=c\sigma_{n}^{(1-\nu_{1}∕2)(1+\theta_{1})d}$ and $p=\nu_{1}$, the Proposition 22 implies that (I) is lower bounded by

$$\;P^{*}([E[L_{\phi }(\hat{f})]+\lambda_{n}{\left\|\hat{f}\right\|}_{𝒢}^{2}-\underset{f\in {𝒜}_{n}(\tau ,{𝒞}_{n})}{\text{inf}}(E[L_{\phi }(f)]+\lambda_{n}{\left\|f\right\|}_{𝒢}^{2})]\geq \xi_{n}^{\left(1\right)})\;\leq P(\exists w\in 𝒲\;\text{with}\;P_{n}(w)\leq 0\;\text{and}\;E(w)\geq \xi_{n}^{1}).\;\leq e^{-x},$$

where

$$\xi_{n}^{\left(1\right)}=c\left(\frac{2M}{c_{1}}\sqrt{\frac{M}{c_{1}\lambda_{n}}+\sigma_{n}^{d}}+2\lambda_{n}\left(\frac{M}{c_{1}\lambda_{1}}+\sigma_{n}^{d}\right)\right)n^{-1∕2}(\sigma_{n}^{(1-\nu_{1}∕2)(1+\theta_{1})d∕2}+2\sqrt{2x}+2x∕\sqrt{n})$$

for some positive c only depends on ($\nu_{1}$, $\theta_{1}$, d), which completes the proof of (16). The risk inequality (17) is guaranteed by Corollary 19 and this completes the proof for T=1.

##### A.2.1.4 Statement of Propositions

In this section, we give the statements of all propositions used for establishing Theorem 11. The first proposition states that the ϵ-covering number of ${ℬ}_{𝒢}$ under $L_{2}(P_{n})$ is uniformly with polynomial order in terms of $\sigma_{n}$ and ϵ. This result was first established as Theorem 2.1 in Steinwart et al. (2006).

**Proposition 21** (Steinwart and Scovel (2007, Theorem 2.1)) For any ϵ>0, we have

$$\underset{P_{n}}{\text{sup}}\log\;𝒩({ℬ}_{𝒢},\epsilon ,L_{2}(P_{n}))\leq c\sigma_{n}^{(1-\nu ∕2)(1+\theta )d}\epsilon^{-\nu }$$

for any 0<ν≤2 and θ>0. Here, ${ℬ}_{𝒢}$ is the closed unit ball in 𝒢 w.r.t. ${\left\|\cdot \right\|}_{𝒢}$ and $𝒩(\cdot ,\epsilon ,L_{2}(P_{n}))$ is the covering number of ϵ-ball w.r.t. empirical $L_{2}(P_{n})$ norm

$${\left\|f\right\|}_{L_{2}(P_{n})}={\left(\frac{1}{n}\sum_{i=1}^{n}f{\left(X_{i}\right)}^{2}\right)}^{1∕2}.$$

c is a constant only depends on (ν, θ, d).

Proposition 22 quantifies the stochastic error of $\hat{f}$, which is a modification of Theorem 5.1 of Steinwart and Scovel (2007). Two preliminary propositions used to establish Proposition 22 are stated as Proposition 23 and Proposition 24 at the end of this section.

**Proposition 22** Let P be a probability measure on 𝒵 and 𝒲 be a set of bounded measurable functions from 𝒵 to R. Suppose that 𝒲 is separable w.r.t. ${\left\|\cdot \right\|}_{\infty }$ and ${\left\|w\right\|}_{\infty }\leq B<\infty$ for all w∈𝒲. Let 𝒮={E(w)−w:w∈𝒲}. Then for all n≥1, h≥1 and

$$\zeta_{n}=3E[\underset{s\in 𝒮}{\text{sup}}\;P_{n}(s)]+2\sqrt{2}B\sqrt{\frac{h}{n}}+2B\frac{h}{n},$$

we have

$$P^{*}(\exists w\in 𝒲\;with\;P_{n}(w)\leq 0\;and\;E(w)\geq \zeta_{n})\leq e^{-h}.$$

Moreover, when

$$\underset{P_{n}}{\text{sup}}\log\;𝒩(B^{-1}𝒲,\epsilon ,L_{2}(P_{n}))\leq l\epsilon^{-p}$$

we have

$$E[\underset{s\in 𝒮}{\text{sup}}\;P_{n}(s)]\geq cB{\left(\frac{l}{n}\right)}^{1∕2}.$$

Here, c is a positive constant that only depends on p.

**Proof** By the assumption of 𝒲 and the definition of 𝒮, it is obvious that E(s)=0, ${\left\|s\right\|}_{\infty }\leq 2B$ and $E(s^{2})\leq 4B^{2}$ for all s∈𝒮. Moreover, it is also easy to verify that 𝒮 is separable w.r.t. ${\left\|\cdot \right\|}_{\infty }$ given 𝒲 is separable w.r.t. ${\left\|\cdot \right\|}_{\infty }$. Note that

$$P^{*}(\exists w\in 𝒲\;\text{with}\;P_{n}(w)\leq 0\;\text{and}\;E(w)\geq \zeta_{n})\;\;\leq P^{*}(\exists w\in 𝒲\;\text{with}\;E(w)-P_{n}(w)\geq \zeta_{n})\;\;\leq P^{n}(\underset{s\in 𝒮}{\text{sup}}\;P_{n}(s)\geq \zeta_{n}).$$

Using Theorem 5.3 from Steinwart and Scovel (2007), which is stated as Proposition 24, with b=2B and $\iota =4B^{2}$, we have

$$P^{n}(\underset{s\in 𝒮}{\text{sup}}\;P_{n}(s)\geq \zeta_{n})=P^{n}\left(\underset{s\in 𝒮}{\text{sup}}\;P_{n}(s)\geq 3E[\underset{s\in 𝒮}{\text{sup}}\;P_{n}(s)]+2\sqrt{2}B\sqrt{\frac{h}{n}}+2B\frac{h}{n}\right)\leq e^{-h},$$

which completes the first part of the proposition. To prove the second part of the proposition, we note that by the definition of 𝒮, we have

$$E[\underset{s\in 𝒮}{\text{sup}}\;P_{n}(s)]\leq E\left[\underset{w\in 𝒲,E(w^{2})\leq B^{2}}{\text{sup}}|E(w)-P_{n}(w)|\right]=\omega_{n}(𝒲,B^{2}),$$

where $\omega_{n}(𝒲,\xi )$ is the modulus of the continuity of 𝒲. Define the local Rademacher complexity of 𝒲 to be

$${\text{Rad}}_{n}(𝒲,\xi )=E_{Z}\left[E_{\epsilon }\left[\underset{w\in 𝒲,E(w^{2})\leq \xi }{\text{sup}}|\frac{1}{n}\sum_{i=1}^{n}\epsilon_{i}f(Z_{i})|\right]\right],$$

where $\left\{\epsilon_{i}\right\}$ are n i.i.d. Rademacher random variables. According to van der Vaart and Wellner (1996), we have

$$\omega_{n}(𝒲,\xi )\leq 2{\text{Rad}}_{n}(𝒲,\xi ).$$

Using the property that for ∀r>0

$${\text{Rad}}_{n}(r𝒲,\xi )=r{\text{Rad}}_{n}(𝒲,r^{-2}\xi )$$

and applying Proposition 5.5 of Steinwart and Scovel (2007), which is stated as Proposition 23, under the condition on the covering number of 𝒲, we have

$$E[\underset{s\in 𝒮}{\text{sup}}\;P_{n}(s)]\leq \omega_{n}(𝒲,B^{2})\leq 2{\text{Rad}}_{n}(𝒲,B^{2})\leq 2B{\text{Rad}}_{n}(B^{-1}𝒲,1)\leq 2cB{\left(\frac{l}{n}\right)}^{1∕2},$$

which completes the second part of the proposition. ■

**Proposition 23** (Steinwart and Scovel (2007, Proposition 5.5)) Let 𝒲 be a class of measurable functions from 𝒵 to [−1, 1] which is separable w.r.t. ${\left\|\cdot \right\|}_{\infty }$ and let P be a probability measure on 𝒵. Assume that there are constants q>0 and 0<p<2 with

$$\underset{P_{n}}{\text{sup}}\log\;N(𝒲,\epsilon ,L_{2}(P_{n}))\leq q\epsilon^{-p}$$

for all ϵ>0. Then there exists a constant c depending only on p such that for all n≥1 and all ϵ>0 we have

$$Rad_{n}(𝒲,\epsilon )\leq c\;\max\left\{\epsilon^{1∕2-p∕4}{\left(\frac{q}{n}\right)}^{1∕2},{\left(\frac{q}{n}\right)}^{2∕(2+p)}\right\}.$$

**Proposition 24** (Steinwart and Scovel (2007, Theorem 5.3)) Let P be a probability measure on 𝒵 and 𝒲 be a set of bounded measurable functions from 𝒵 to R which is separable w.r.t. ${\left\|\cdot \right\|}_{\infty }$ and satisfies E(w)=0 for all w∈𝒲. Furthermore, let b>0 and ι≥0 be constants with ${\left\|w\right\|}_{\infty }\leq b$ and $E(w^{2})\leq \iota$ for all w∈𝒲. Then for all x≥1 and all n≥1 we have

$$P^{n}\left(\underset{w\in 𝒲}{\text{sup}}\;P_{n}(w)>3E[\underset{w\in 𝒲}{\text{sup}}\;P_{n}(w)]+\sqrt{\frac{2x\iota }{n}}+\frac{bx}{n}\right)\leq e^{-x}.$$

#### A.2.2 Proof of Theorem 11 for T≥2

We first prove (16) is Theorem 11. To this end, we define

$$L_{\phi ,t}(f_{t};f_{t+1},\ldots ,f_{T})=E\left[\frac{\left(Y_{t}+U_{t+1}(H_{t+1};f_{t+1},\ldots ,f_{T})\right)}{p(A_{t}|H_{t})}\phi (A_{t}f_{t}(H_{t}))\right],$$

and

(30)
$${\tilde{V}}_{t}=\underset{f_{t}\in {𝒜}_{t}(\tau_{t})}{\text{sup}}{𝒱}_{t}(f_{t},{\hat{f}}_{t+1},\ldots ,{\hat{f}}_{T}),$$

where

$${𝒱}_{t}(g_{t},\ldots ,g_{T})=E\left[\frac{\left(\sum_{j=t}^{T}Y_{j})\prod_{j=t}^{T}I(A_{j}g_{j}(H_{j})>0\right)}{\prod_{j=t}^{T}p(A_{j}|H_{j})}\right].$$

Note that the Fisher consistency in Theorem 2 indicates that ${𝒱}_{t}(g_{t}^{*},\ldots ,g_{T}^{*})={𝒱}_{t}(f_{t}^{*},\ldots ,f_{T}^{*})$, and it is sufficient to derive an upper bound for ${𝒱}_{t}(f_{t}^{*},\ldots ,f_{T}^{*})-{𝒱}_{t}({\hat{f}}_{t},\ldots ,{\hat{f}}_{T})$.

First, note that by repeating the same argument as for T=1, we can show ${\left\|{\hat{f}}_{t}\right\|}_{{𝒢}_{t}}$ is bounded by ${𝒞}_{n,t}=c\sqrt{\frac{(T-1+t)M}{c_{1}\lambda_{n,t}}+\sigma_{n,t}^{d_{t}}}$ with probability at least $1-2\;\text{exp}\left(-\frac{2n\delta_{0,t}^{2}c_{1}^{2}}{{\left(T-t+1\right)}^{2}M^{2}}\right)$ for any t=1,..,T. Hence, we can replace ${𝒜}_{t}(\tau_{t})$ in (30) by ${𝒜}_{t}(\tau_{t},{𝒞}_{n,t})$ with high probability and obtain

$$\;{𝒱}_{t}(f_{t}^{*},\ldots ,f_{T}^{*})-{𝒱}_{t}({\hat{f}}_{t},\ldots ,{\hat{f}}_{T})\;={𝒱}_{t}(f_{t}^{*},\ldots ,f_{T}^{*})-{\tilde{V}}_{t}+{\tilde{V}}_{t}-{𝒱}_{t}({\hat{f}}_{t},\ldots ,{\hat{f}}_{T})\;\leq \underset{\left(I\right)}{\underset{︸}{{𝒱}_{t}(f_{t}^{*},\ldots ,f_{T}^{*})-{\tilde{V}}_{t}}}+\underset{\left(II\right)}{\underset{︸}{L_{\phi ,t}({\hat{f}}_{t};{\hat{f}}_{t+1},\ldots ,{\hat{f}}_{T})-\underset{f_{t}\in {𝒜}_{t}(\tau_{t},{𝒞}_{n,t})}{\text{inf}}L_{\phi ,t}(f_{t};{\hat{f}}_{t+1},\ldots ,{\hat{f}}_{T})}}+(T-t+1)M\eta_{n,t},$$

where

𝒜t(τt,𝒞n,t)={f∈𝒢t∣‖f‖𝒢t≤𝒞n,t,E[Rtψ(Atf(Ht),ηn,t)p(At∣Ht)]≤τt∣},

and to obtain the last inequality we have used the fact that ${|Q_{t}|}_{\infty }\leq (T-t+1)M$ and the excessive risk inequality of Lemma 12 to replace the difference under 0-1 loss in terms of ${𝒱}_{t}$ by the difference under hinge loss in terms of $L_{\phi ,t}$.

For (I), we have

$$(I)\leq {𝒱}_{t}(f_{t}^{*},\ldots ,f_{T}^{*})-\underset{f_{t}\in {𝒜}_{t}(\tau_{t},{𝒞}_{n,t})}{\text{sup}}[{𝒱}_{t}(f_{t},{\hat{f}}_{t+1},\ldots ,{\hat{f}}_{T})-{𝒱}_{t}(f_{t},f_{t+1}^{*},\ldots ,f_{T}^{*})+{𝒱}_{t}(f_{t},f_{t+1}^{*},\ldots ,f_{T}^{*})]\;\;\leq {𝒱}_{t}(f_{t}^{*},\ldots ,f_{T}^{*})-\underset{f_{t}\in {𝒜}_{t}(\tau_{t},{𝒞}_{n,t})}{\text{sup}}{𝒱}_{t}(f_{t},f_{t+1}^{*},\ldots ,f_{T}^{*})+c_{1}^{-1}|{𝒱}_{t+1}(f_{t+1}^{*},\ldots ,f_{T}^{*})-{𝒱}_{t+1}({\hat{f}}_{t+1},\ldots ,{\hat{f}}_{T})|\;\;=c_{1}^{-1}|{𝒱}_{t+1}(f_{t+1}^{*},\ldots ,f_{T}^{*})-{𝒱}_{t+1}({\hat{f}}_{t+1},\ldots ,{\hat{f}}_{T})|+(T-t+1)M\eta_{n,t},\;\;+\underset{\left(III\right)}{\underset{︸}{\underset{f_{t}\in {𝒜}_{t}(\tau_{t},{𝒞}_{n,t})}{\text{inf}}L_{\phi ,t}(f_{t};f_{t+1}^{*},\ldots ,f_{T}^{*})-L_{\phi ,t}(f_{t}^{*};f_{t+1}^{*},\ldots ,f_{T}^{*})}}$$

where again we have used the fact that ${|Q_{t}|}_{\infty }\leq (T-t+1)M$ and the excessive risk inequality of Lemma 12. To bound the last term in (I), recall that $f_{t,\tau }^{*}$ denotes the solution of (4) at stage t with risk constraint $\tau_{t}$ replaced by τ. Then the second part of Lemma 18 indicates that $V_{\sigma_{n,t}}{\overset{ˇ}{f}}_{t,\tau_{t}-\epsilon_{n,t}^{\prime }}\in {𝒜}_{t}(\tau_{t}-\epsilon_{n,t},{𝒞}_{n,t})\subseteq {𝒜}_{t}(\tau_{t},{𝒞}_{n,t})$, where ${\overset{ˇ}{f}}_{t,\tau_{t}-\epsilon_{n,t}^{\prime }}=(\sigma_{n,t}^{2}∕\pi ){\bar{f}}_{t,\tau_{t}-\epsilon_{n,t}^{\prime }}$, and

$$|L_{\phi ,t}(V_{\sigma_{n,t}}{\overset{ˇ}{f}}_{t,\tau_{t}-\epsilon_{n,t}^{\prime }};f_{t+1}^{*},\ldots ,f_{T}^{*})-L_{\phi ,t}(f_{t,\tau_{t}-\epsilon_{n,t}^{\prime }}^{*};f_{t+1}^{*},\ldots ,f_{T}^{*})|\leq c\sigma_{n,t}^{-\alpha_{t}d_{t}}.$$

Therefore, we have

$$\left(III)\leq L_{\phi ,t}(V_{\sigma_{n,t}}{\overset{ˇ}{f}}_{t,\tau_{t}-\epsilon_{n,t}^{\prime }};f_{t+1}^{*},\ldots ,f_{T}^{*})-L_{\phi ,t}(f_{t}^{*};f_{t+1}^{*},\ldots ,f_{T}^{*})\;\;\leq |L_{\phi ,t}(V_{\sigma_{n,t}}{\overset{ˇ}{f}}_{t,\tau_{t}-\epsilon_{n,t}^{\prime }};f_{t+1}^{*},\ldots ,f_{T}^{*})-L_{\phi ,t}(f_{t,\tau_{t}-\epsilon_{n,t}^{\prime }}^{*};f_{t+1}^{*},\ldots ,f_{T}^{*})|\;\;+L_{\phi ,t}(f_{t,\tau_{t}-\epsilon_{n,t}^{\prime }}^{*};f_{t+1}^{*},\ldots ,f_{T}^{*})-L_{\phi ,t}(f_{t}^{*};f_{t+1}^{*},\ldots ,f_{T}^{*})\;\;\leq c(\sigma_{n,t}^{-\alpha_{t}d_{t}}+\epsilon_{n,t}^{\prime })\leq O(\epsilon_{n,t}^{\prime }\right)$$

where to obtain the last inequality we used the Lipschitz continuity of the value function in Lemma 14 and by definition $f_{t}^{*}=f_{t,\tau_{t}}^{*}$.

For (II), we have

(31)
(II)≤Lϕ,t(f^t;f^t+1,…,f^T)+λn,t‖f^t‖𝒢t2−infft∈𝒜t(τt,𝒞n,t)Lϕ,t(ft;f^t+1,…,f^T)=[Lϕ,t(f^t;f^t+1,…,f^T)+λn,t‖f^t‖𝒢t2]−infft∈𝒜t,n(τt,𝒞n,t)[(Lϕ,t(ft;f^t+1,…,f^T)+λn,t‖ft‖𝒢t2)]+[infft∈𝒜t,n(τt,𝒞n,t)(Lϕ,t(ft;f^t+1,…,f^T)+λn,t‖ft‖𝒢t2)]−[infft∈𝒜t(τt,𝒞n,t)Lϕ,t(ft;f^t+1,…,f^T)],

where

𝒜t,n(τ,𝒞n,t)={f∈𝒢t∣‖f‖𝒢t≤𝒞n,t,1n∑i=1nRitψ(Aitf(Hit),ηn,t)p(Ait∣Hit)≤τt∣}.

The first term on the right-hand side of the inequality (31) can be bounded by

$$\;L_{\phi ,t}({\hat{f}}_{t};{\hat{f}}_{t+1},\ldots ,{\hat{f}}_{T})+\lambda_{n,t}{\left\|{\hat{f}}_{t}\right\|}_{{𝒢}_{t}}^{2}-\underset{f_{t}\in {𝒜}_{t,n}(\tau_{t},{𝒞}_{n,t})}{\text{inf}}\left(L_{\phi ,t}(f_{t};{\hat{f}}_{t+1},\ldots ,{\hat{f}}_{T})+\lambda_{n,t}{\left\|f_{t}\right\|}_{{𝒢}_{t}}^{2}\right)\;\leq 2c_{1}^{-1}|{𝒱}_{t+1}(f_{t+1}^{*},\ldots ,f_{T}^{*})-{𝒱}_{t+1}({\hat{f}}_{t+1},\ldots ,{\hat{f}}_{T})|\;\;+\underset{\left(IV\right)}{\underset{︸}{\left[L_{\phi ,t}({\hat{f}}_{t};f_{t+1}^{*},\ldots ,f_{T}^{*})+\lambda_{n,t}{\left\|{\hat{f}}_{t}\right\|}_{{𝒢}_{t}}^{2}-\underset{f_{t}\in {𝒜}_{t,n}(\tau_{t},{𝒞}_{n,t})}{\text{inf}}\left(L_{\phi ,t}(f_{t};f_{t+1}^{*},\ldots ,f_{T}^{*})+\lambda_{n,t}{\left\|f_{t}\right\|}_{{𝒢}_{t}}^{2}\right)\right]}},$$

and (IV) is equal to stochastic error term (I) in (29) for the proof of T=1 with Y being replaced by $Q_{t}$. Note that $|Q_{t}|\leq (T-t+1)M$ and consequently (IV) can be bounded using exactly the same argument as for term (I) of (29), which turns out to have order $O(\xi_{n,t}^{\left(1\right)})$ with probability at least $1-\text{exp}(-x_{t})$. For the second term of (31), we have

$$\;\underset{f_{t}\in {𝒜}_{t,n}(\tau_{t},{𝒞}_{n,t})}{\text{inf}}\left(L_{\phi ,t}(f_{t};{\hat{f}}_{t+1},\ldots ,{\hat{f}}_{T})+\lambda_{n,t}{\left\|f_{t}\right\|}_{{𝒢}_{t}}^{2}\right)-\underset{f_{t}\in {𝒜}_{t}(\tau_{t},{𝒞}_{n,t})}{\text{inf}}L_{\phi ,t}(f_{t};{\hat{f}}_{t+1},\ldots ,{\hat{f}}_{T})\;\leq 2c_{1}^{-1}|{𝒱}_{t+1}(f_{t+1}^{*},\ldots ,f_{T}^{*})-{𝒱}_{t+1}({\hat{f}}_{t+1},\ldots ,{\hat{f}}_{T})|\;\;+\underset{\left(V\right)}{\underset{︸}{\left[\underset{f_{t}\in {𝒜}_{t,n}(\tau_{t},{𝒞}_{n,t})}{\text{inf}}\left(L_{\phi ,t}(f_{t};f_{t+1}^{*},\ldots ,f_{T}^{*})+\lambda_{n,t}{\left\|f_{t}\right\|}_{{𝒢}_{t}}^{2}\right)-\underset{f_{t}\in {𝒜}_{t}(\tau_{t},{𝒞}_{n,t})}{\text{inf}}L_{\phi ,t}(f_{t};f_{t+1,\tau_{t+1}}^{*},\ldots ,f_{T}^{*})\right]}},$$

Note that term (V) is similar to the sum of the remaining terms in (29) also with Y being replaced by $Q_{t}$. Hence, following the same argument as for T=1, (V) can be similarly decomposed to terms (II) − (IV) in (29) and bounded separately, which turns out to have order $O(\xi_{n,t}^{\left(2\right)})+O(\epsilon_{n,t}^{\prime })$ in total with probability at least $1-2\;\text{exp}\left(-\frac{n\delta_{t}^{2}c_{1}^{2}}{2{\left(T-t+1\right)}^{2}M^{2}}\right)$. Combing these results, we conclude that with probability at least $1-h_{n}(t,x_{t})$,

(32)
$${𝒱}_{t}(f_{t}^{*},\ldots ,f_{T}^{*})-{𝒱}_{t}({\hat{f}}_{t},\ldots ,{\hat{f}}_{T})\leq 5c_{1}^{-1}|{𝒱}_{t+1}(f_{t+1}^{*},\ldots ,f_{T}^{*})-{𝒱}_{t+1}({\hat{f}}_{t+1},\ldots ,{\hat{f}}_{T})|\;\;+c\eta_{n,t}^{-1}+\underset{\left(III\right)}{\underset{︸}{c(\epsilon_{n,t}^{\prime })}}+\underset{\left(IV)+(V\right)}{\underset{︸}{c(\xi_{n,t}^{\left(1\right)}+\xi_{n,t}^{\left(2\right)}+\epsilon_{n,t}^{\prime })}}\;\;\leq 5c_{1}^{-1}|{𝒱}_{t+1}(f_{t+1}^{*},\ldots ,f_{T}^{*})-{𝒱}_{t+1}({\hat{f}}_{t+1},\ldots ,{\hat{f}}_{T})|\;\;+c(\xi_{n,t}+\epsilon_{n,t}^{\prime }+\eta_{n,t}^{-1})$$

for some constant c.

On the other hand, according to Lemma 18, similar to the prove when T=1 we can show that ${\hat{f}}_{t}\in {𝒜}_{t,n}(\tau_{t},{𝒞}_{n,t})\subseteq {𝒜}_{t}(\tau_{t}+\epsilon_{n,t}^{\prime },{𝒞}_{n,t})$ with probability at least $1-2\;\text{exp}\left(\frac{n\delta_{t}^{2}c_{1}^{2}}{2{\left(T-t+1\right)}^{2}M^{2}}\right)$. Therefore, we have

(33)
$$\;{𝒱}_{t}(f_{t}^{*},\ldots ,f_{T}^{*})-{𝒱}_{t}({\hat{f}}_{t},\ldots ,{\hat{f}}_{T})\;\geq {𝒱}_{t}(f_{t}^{*},\ldots ,f_{T}^{*})-\underset{f_{t}\in {𝒜}_{t}(\tau_{t}+\epsilon_{n,t}^{\prime },{𝒞}_{n,t})}{\text{sup}}{𝒱}_{t}(f_{t},{\hat{f}}_{t+1},\ldots ,{\hat{f}}_{T})\;\geq {𝒱}_{t}(f_{t}^{*},\ldots ,f_{T}^{*})-{𝒱}_{t}(f_{t,\tau_{t}+\epsilon_{n,t}^{\prime }}^{*},f_{t+1}^{*},\ldots ,f_{T}^{*})\;\;-c_{1}^{-1}|{𝒱}_{t}(f_{t+1}^{*},\ldots ,f_{T}^{*})-{𝒱}_{t}({\hat{f}}_{t+1},\ldots ,{\hat{f}}_{T})|\;\geq c\epsilon_{n,t}^{\prime }-c_{1}^{-1}|{𝒱}_{t}(f_{t+1}^{*},\ldots ,f_{T}^{*})-{𝒱}_{t}({\hat{f}}_{t+1},\ldots ,{\hat{f}}_{T})|.$$

Finally, by combining (32) and (33), we obtain that with probability at least $1-h_{n}(t,x_{t})$,

$$|{𝒱}_{t}(f_{t}^{*},\ldots ,f_{T}^{*})-{𝒱}_{t}({\hat{f}}_{t},\ldots ,{\hat{f}}_{T})|\leq 5c_{1}^{-1}|{𝒱}_{t+1}(f_{t+1}^{*},\ldots ,f_{T}^{*})-{𝒱}_{t+1}({\hat{f}}_{t+1},\ldots ,{\hat{f}}_{T})|\;\;+c(\xi_{n,t}+\epsilon_{n,t}^{\prime }+\eta_{n,t}^{-1}).$$

Hence, (16) in Theorem 11 follows by induction starting from t=T to 1. The error bound of risk (17) can be established by repeating the same proof as Corollary 19 for each stage. This completes the proof of Theorem 11.

## Appendix B: DC Algorithm for Solving Single-Stage BR-DTRs

In this section, we describe the DC algorithm for solving BR-DTRs at stage t. The algorithm was originally proposed in Wang et al. (2018) for T=1. Given estimated rules (${\hat{f}}_{t+1},\ldots ,{\hat{f}}_{T}$), one can calculate $\left\{{\hat{Y}}_{it}\right\}$ and $\left\{{\hat{A}}_{it}\right\}$ from (6). We aim to solve the optimization problem

$$\underset{\beta \in R^{d},\beta_{0}\in R}{\text{min}}\;\;C_{n,t}\sum_{i=1}^{n}\frac{{\hat{Y}}_{it}}{p(A_{it}|H_{it})}\phi ({\hat{A}}_{it}(K_{i,t}\beta +\beta_{0}))+\frac{1}{2}\beta^{T}K_{t}\beta \;\text{subject to}\;\;\sum_{i=1}^{n}\frac{R_{it}}{p(A_{it}|H_{it})}\psi (A_{it}(K_{i,t}\beta +\beta_{0}),\eta )\leq n\tau_{t},$$

where $C_{n,t}={\left(2n\lambda_{n,t}\right)}^{-1}$. Here, $K_{t}$ is the n-by-n kernel matrix of stage t defined by $K_{ij}=K_{t}(H_{it},H_{jt})$ where $K_{t}(\cdot ,\cdot )$ is the inner product equipped by RKHS ${𝒢}_{t}$ and $K_{i,t}$ is the i-th row of $K_{t}$.

Note that the shifted ramp loss can be decomposed as $\psi (x,\eta )=\eta^{-1}{\left(x+\eta \right)}_{+}-\eta^{-1}{\left(x\right)}_{+}$. By applying the DC algorithm, given $\beta^{\left(s\right)}$ and $\beta_{0}^{\left(s\right)}$, we update ($\beta^{\left(s+1\right)},\beta_{0}^{\left(s+1\right)}$) by solving the optimization problem

$$\underset{\beta \in R^{d_{t}},\beta_{0}\in R}{\text{min}}\;\;C_{n,t}\sum_{i=1}^{n}\frac{{\hat{Y}}_{it}}{p(A_{it}|H_{it})}\phi ({\hat{A}}_{it}(K_{i,t}\beta +\beta_{0}))+\frac{1}{2}\beta^{T}K_{t}\beta \;\text{subject to}\;\;\sum_{i=1}^{n}\frac{R_{it}}{p(A_{it}|H_{it})}\left[{\left\{A_{it}(K_{i,t}\beta +\beta_{0})+\eta \right\}}_{+}-C_{it}^{\left(s\right)}A_{it}(K_{i,t}\beta +\beta_{0})\right]\leq n\eta \tau_{t},$$

where $C_{it}^{\left(s\right)}=I(A_{it}(K_{i,t}\beta^{\left(s\right)}+\beta_{0}^{\left(s\right)})>0)$. Similar to standard SVM, we introduce slacking variables $\xi_{i}\geq 1-{\hat{A}}_{it}(K_{i,t}\beta +\beta_{0})$, $\xi_{i}\geq 0$ to replace $\phi ({\hat{A}}_{it}(K_{i,t}\beta +\beta_{0}))$ in the objective function. Moreover, we introduce additional slacking variables $\zeta_{i}\geq A_{it}(K_{i,t}\beta +\beta_{0})+\eta$, $\zeta_{i}\geq 0$ to replace ${\left\{A_{it}(K_{i,t}\beta +\beta_{0})+\eta \right\}}_{+}$ in the risk constraint. After plugging the slacking variables, the optimization problem becomes

(34)
$$\underset{\beta \in R^{d_{t}},\beta_{0}\in R}{\text{min}}\;\;C_{n,t}\sum_{i=1}^{n}\frac{{\hat{Y}}_{it}}{p(A_{it}|H_{it})}\xi_{i}+C_{n,t}\sum_{i=1}^{n}\frac{\zeta_{i}}{n}+\frac{1}{2}\beta^{T}K_{t}\beta \;\text{subject to}\;\;\sum_{i=1}^{n}\frac{R_{it}}{p(A_{it}|H_{it})}\left[\zeta_{i}-C_{it}^{\left(s\right)}A_{it}(K_{i,t}\beta +\beta_{0})\right]\leq n\eta \tau_{t},\;\;1-{\hat{A}}_{it}(K_{i,t}\beta +\beta_{0})\leq \xi_{i},\;\;0\leq \xi_{i},\;\;A_{it}(K_{i,t}\beta +\beta_{0})+\eta \leq \zeta_{i},\;\;0\leq \zeta_{i},\;\;\text{for}\;i=1,\ldots ,n.$$

The additional term $C_{n,t}\sum_{i=1}^{n}\frac{\zeta_{i}}{n}$ in the objective function is to guarantee that the slacking variable $\zeta_{i}$ is equal to ${\left\{A_{it}(K_{i,t}\beta +\beta_{0}+\eta \right\}}_{+}$. For fixed tuning parameter $C_{n,t}$, this optimization problem will be equivalent to the original problem as the additional term will eventually vanish when the sample size n increases.

The Lagrange function of (34) is given by

$$L=C_{n,t}\left(\sum_{i=1}^{n}\frac{{\hat{Y}}_{it}}{p(A_{it}|H_{it})}\xi_{i}+\sum_{i=1}^{n}\frac{\zeta_{i}}{n}\right)+\frac{1}{2}\beta^{T}K_{t}\beta \;\;-\pi \left[n\eta \tau_{t}-\sum_{i=1}^{n}\frac{R_{it}}{p(A_{it}|H_{it})}\left(\zeta_{i}-\sum_{i=1}^{n}C_{it}^{\left(s\right)}A_{it}(K_{i,t}\beta +\beta_{0})\right)\right]\;\;-\sum_{i=1}^{n}\alpha_{i}\left[\xi_{i}-1+{\hat{A}}_{it}(K_{i,t}\beta +\beta_{0})\right]-\sum_{i=1}^{n}\mu_{i}\xi_{i}-\sum_{i=1}^{n}\kappa_{i}\left[\zeta_{i}-\eta -A_{it}(K_{i,t}\beta +\beta_{0})\right]-\sum_{i=1}^{n}\rho_{i}\kappa_{i}.$$

Taking derivatives w.r.t. $\xi_{i}$, $\zeta_{i}$, β and $\beta_{0}$, one can obtain that the optimal Lagrange multipliers $\alpha =(\alpha_{1},\ldots ,\alpha_{n}^{T})$, $\kappa ={\left(\kappa_{1},\ldots ,\kappa_{n}\right)}^{T}$, $\mu =(\mu_{1},\ldots ,\mu_{n})$, $\rho =(\rho_{1},\ldots ,\rho_{n})$ and π satisfy

$$\;C_{n,t}V_{t,Y}-\alpha -\mu =0,\;\;C_{n,t}1∕n+\pi V_{t,R}-\kappa -\rho =0,\;\;\beta -\pi V_{t,R,A,C}^{\left(s\right)}-{\hat{A}}_{t}\alpha +A_{t}\kappa =0,\;\pi 1^{T}V_{t,R,A,C}^{\left(s\right)}+1^{T}{\hat{A}}_{t}\alpha -1^{T}A_{t}\kappa =0,$$

where 1 and 0 denote the n-by-1 vectors with all entries equal to 1 and 0 respectively,

$$V_{t,Y}=\left[\begin{array}{c} \frac{{\hat{Y}}_{1t}}{p(A_{1t}|H_{1t})}\\ \vdots \\ \frac{{\hat{Y}}_{nt}}{p(A_{nt}|H_{nt})} \end{array}\right],\;\;V_{t,R}=\left[\begin{array}{c} \frac{R_{1t}}{p(A_{1t}|H_{1t})}\\ \vdots \\ \frac{R_{nt}}{p(A_{nt}|H_{nt})} \end{array}\right],\;\;V_{t,R,A,C}^{\left(s\right)}=\left[\begin{array}{c} \frac{R_{1t}}{p(A_{1t}|H_{1t})}A_{1t}C_{1t}^{\left(s\right)}\\ \vdots \\ \frac{R_{nt}}{p(A_{nt}|H_{nt})}A_{nt}C_{nt}^{\left(s\right)} \end{array}\right].$$

Here, we abuse the notation and define ${\hat{A}}_{t}=\text{diag}\{({\hat{A}}_{1t},\ldots ,{\hat{A}}_{nt})\}$ and $A_{t}=\text{diag}\{(A_{1t},\ldots ,A_{nt})\}$. Plugging the equations back to L and note that α≥0, κ≥0, μ≥0, ρ≥0 and π≥0, after some algebra one can obtain that the dual problem of (34) w.r.t. $\omega ={\left(\pi ,\alpha^{T},\kappa^{T}\right)}^{T}$ is given by

$$\;\underset{\omega }{\text{min}}\;\frac{1}{2}\omega^{T}(H^{T}K_{t}H)\omega -\omega^{T}l_{\eta ,\tau_{t}}\;\text{subject to}\;\;a\leq W\omega \leq b,\;\;0_{(2n+1)\times 1}\leq \omega \leq u,$$

where

$$H=\left[V_{t,R,A,C}^{\left(s\right)}\;\;{\hat{A}}_{t}\;\;-A_{t}\right],\;\;W=\left[\begin{array}{ccc} V_{t,R} & 0_{n\times n} & -I_{n}\\ 1^{T}V_{t,R,A,C}^{\left(s\right)} & 1^{T}{\hat{A}}_{t} & -1^{T}A_{t} \end{array}\right],$$

$l_{\eta ,\tau_{t}}={\left(-n\eta \tau_{t},1^{T},\eta 1^{T}\right)}^{T}$, $a={\left(-C_{n,t}1^{T}∕n,0\right)}^{T}$, $b={\left(\infty 1^{T},0\right)}^{T}$ and $u={\left(\infty ,C_{n,t}V_{t,Y}^{T},\infty 1^{T}\right)}^{T}$. Note that the optimization w.r.t. ω is a standard quadratic optimization problem, which can be solved efficiently using standard software. Denote the optimal solution of the previous optimization problem by ${\hat{\omega }}^{\left(s\right)}$, we update β by

$$\beta^{\left(s+1\right)}={\hat{\pi }}^{\left(s\right)}V_{t,R,A,C}^{\left(s\right)}+{\hat{A}}_{t}{\hat{\alpha }}^{\left(s\right)}-A_{t}{\hat{\kappa }}^{\left(s\right)}.$$

The new $\beta_{0}^{\left(s+1\right)}$ can be determined via grid search such that the original objective function is maximized among values that satisfy the constraint given $\beta =\beta^{\left(s+1\right)}$. We stop the iteration when the termination condition $\max({|\beta^{\left(s+1\right)}-\beta^{\left(s\right)}|}_{\infty },|\beta_{0}^{\left(s+1\right)}-\beta^{\left(s\right)}|)\leq \epsilon$ is satisfied or reaches the maximum iteration limitation. Let $\hat{\beta }={\left({\hat{\beta }}_{1},\ldots ,{\hat{\beta }}_{n}\right)}^{T}$ and ${\hat{\beta }}_{0}$ denote the final solution returned by DC iteration, then the final estimated decision function at stage t is given by ${\hat{f}}_{t}(\cdot )=\sum_{i=1}^{n}K_{t}(H_{it},\cdot ){\hat{\beta }}_{i}+{\hat{\beta }}_{0}$.

## Appendix C: Additional Simulation Results

### Appendix C.1. Simulation results for setting I with τ=1.5 and setting II with τ=1.3

**Table S.1:** Estimated reward/risk on independent testing data for setting $I-\tau_{1}=\tau_{2}=1.5$, setting $\text{II}-\tau_{1}=\tau_{2}=1.3$ and n=400 under 3 different methods using linear/Gaussian kernel. Results are reported in the same format as Table 1.

Setting	η	Method	Linear Kernel				Gaussian Kernel			
			Reward - II	Risk - II	Cumulative Reward	Risk - I	Reward - II	Risk - II	Cumulative Reward	Risk - I
Setting I	0.02	BR-DTRs	1.557(0.080)	1.502(0.070)	2.443(0.082)	1.475(0.066)	1.551(0.096)	1.497(0.084)	2.436(0.091)	1.475(0.068)
	0.02	Naive	—	—	2.339(0.096)	1.460(0.083)	—	—	2.318(0.115)	1.448(0.097)
	0.04	BR-DTRs	1.568(0.082)	1.510(0.072)	2.429(0.087)	1.467(0.066)	1.546(0.096)	1.493(0.084)	2.425(0.089)	1.463(0.066)
	0.04	Naive	—	—	2.319(0.098)	1.430(0.088)	—	—	2.304(0.113)	1.428(0.096)
	0.06	BR-DTRs	1.563(0.075)	1.507(0.066)	2.420(0.081)	1.455(0.059)	1.545(0.095)	1.492(0.084)	2.414(0.091)	1.450(0.063)
	0.06	Naive	—	—	2.306(0.100)	1.416(0.084)	—	—	2.292(0.108)	1.411(0.093)
	0.08	BR-DTRs	1.546(0.082)	1.493(0.071)	2.387(0.075)	1.439(0.059)	1.541(0.096)	1.489(0.084)	2.393(0.091)	1.438(0.061)
	0.08	Naive	—	—	2.274(0.112)	1.397(0.083)	—	—	2.277(0.110)	1.400(0.089)
	0.1	BR-DTRs	1.544(0.093)	1.489(0.082)	2.371(0.068)	1.421(0.054)	1.539(0.093)	1.486(0.082)	2.375(0.086)	1.424(0.060)
	0.1	Naive	—	—	2.273(0.093)	1.383(0.080)	—	—	2.268(0.104)	1.387(0.083)
		AOWL	1.983(0.010)	2.149(0.044)	3.257(0.018)	2.678(0.096)	1.914(0.030)	2.099(0.083)	3.212(0.036)	2.584(0.218)
Setting II	0.02	BR-DTRs	1.297(0.132)	1.159(0.196)	3.128(0.178)	1.221(0.116)	1.380(0.114)	1.285(0.169)	3.019(0.184)	1.240(0.079)
	0.02	Naive	—	—	1.797(0.000)	0.166(0.000)	—	—	1.797(0.000)	0.166(0.000)
	0.04	BR-DTRs	1.209(0.168)	1.034(0.252)	3.150(0.157)	1.215(0.099)	1.347(0.139)	1.231(0.206)	2.991(0.192)	1.218(0.077)
	0.04	Naive	—	—	1.797(0.000)	0.166(0.000)	—	—	1.797(0.000)	0.166(0.000)
	0.06	BR-DTRs	1.131(0.238)	0.917(0.351)	3.109(0.179)	1.193(0.129)	1.349(0.174)	1.233(0.253)	2.983(0.194)	1.213(0.078)
	0.06	Naive	—	—	1.797(0.000)	0.166(0.000)	—	—	1.797(0.000)	0.166(0.000)
	0.08	BR-DTRs	1.080(0.299)	0.821(0.440)	3.102(0.172)	1.182(0.115)	1.335(0.168)	1.202(0.247)	2.937(0.199)	1.202(0.088)
	0.08	Naive	—	—	1.797(0.000)	0.166(0.000)	—	—	1.797(0.000)	0.166(0.000)
	0.1	BR-DTRs	0.893(0.394)	0.559(0.585)	3.067(0.205)	1.164(0.14)	1.350(0.155)	1.229(0.235)	2.893(0.217)	1.173(0.087)
	0.1	Naive	—	—	1.797(0.000)	0.166(0.000)	—	—	1.797(0.000)	0.166(0.000)
		AOWL	2.440(0.064)	3.017(0.002)	5.188(0.000)	2.839(0.000)	2.424(0.080)	3.018(0.002)	5.188(0.000)	2.839(0.000)

### Appendix C.2. An additional simulation with an unknown and unbalanced study design

In this section, we conduct an additional simulation study under setting II but now assume that the treatment assignment depends on covariates as

$$\log\text{it}\;p(A_{1}|H_{1})=-X_{1}+0.25,\;\;\log\text{it}\;p(A_{2}|H_{2})=-X_{1}+X_{2}-0.25,$$

and treatment probabilities are unknown and will be estimated from data as in observational studies. We conduct the simulation following a similar procedure as described in Section 4 with $\tau_{1}=\tau_{2}=1.5$ except that in the training step, we estimate the unknown treatment assignment probability via the Lasso logistic regression using all observed features as predictors. The results are presented in Figure S.4 and Table S.2 and the conclusions are similar to the findings in Section 4.

### Appendix C.3. A simulated application to personalized promotion in E-commerce

In this section, we simulate a toy personalized promotion problem and apply BR-DTRs to explore the performance on more general constrained decision-making problems. In this simulation, we let T=4 and ${\left\{A_{t}\right\}}_{t=1}^{4}$ denote the promotion action taken at wave t=1,…,4 with $A_{t}\in \{-1,+1\}$ denoting two available promotions. We use ${\left\{(Y_{t},C_{t})\right\}}_{t=1}^{4}$ to denote the instant commercial reward and the promotion cost after taking actions ${\left\{A_{t}\right\}}_{t=1}^{4}$. We assume that five baseline features ($X_{1},\ldots ,X_{5}$) are available for each customer. In this simulation, the instant commercial rewards and the promotion costs are generated according to

$$Y_{1}=1+X_{2}+(1+A_{1})(X_{1}+X_{2})+\epsilon_{Y_{1}},\;R_{1}=1+2(1+A_{1})(2X_{1}+1)+\epsilon_{R_{1}},\;Y_{2}=1.5+X_{2}+(1+A_{2})(Y_{1}∕4+A_{1}∕2+1∕2)+\epsilon_{Y_{2}},\;R_{2}=1+2(1+A_{2})(X_{1}+1)+\epsilon_{R_{2}},\;Y_{3}=1.5+X_{2}+(1+A_{3})(Y_{2}∕4+A_{2}∕2+1∕2)+\epsilon_{Y_{3}},\;R_{3}=1+2(1+A_{3})(X_{1}+1)+\epsilon_{R_{3}},\;Y_{4}=1.5+X_{2}+(1+A_{4})(Y_{3}∕4+A_{3}∕2+1∕2)+\epsilon_{Y_{4}},\;R_{4}=1+2(1+A_{4})(X_{1}+1)+\epsilon_{R_{4}},$$

where ${\left\{\epsilon_{Y_{t}}\right\}}_{t=1}^{4}$ and ${\left\{\epsilon_{R_{t}}\right\}}_{t=1}^{4}$ denote independent random noisy terms with distribution Unif[−0.5, 0.5].

We assume that the promotion has been delivered to n=400 customers in a pilot study with equal probability, i.e., $P(A_{t}|X_{1},\ldots ,X_{5})=0.5$ for t=1,…,4. Our goal is to learn an optimal strategy from observed data

$${\left\{(A_{i1},Y_{i1},R_{i1},\ldots ,A_{i4},Y_{i4},R_{i4},X_{i1},\ldots ,X_{i5})\right\}}_{i=1}^{n}$$

to determine which promotion should be delivered to customers so that the cumulative commercial reward $Y=\sum_{t=1}^{4}Y_{t}$ is maximized at a cost no larger than ${\left\{\tau_{t}\right\}}_{t=1}^{4}$ for each wave according to customers’ baseline features, and the promotion costs and instant rewards from the previous waves of promotions. We note that BR-DTRs can be applied to handle the problem by treating ${\left\{Y_{t}\right\}}_{t=1}^{4}$ and ${\left\{C_{t}\right\}}_{t=1}^{4}$ as the reward and stagewise risks respectively, with $H_{1}=(X_{1},\ldots ,X_{5})$, $H_{2}=(H_{1},A_{1},Y_{1},R_{1})$, $H_{3}=(H_{2},A_{2},Y_{2},R_{2})$, and $H_{4}=(H_{3},A_{3},Y_{3},R_{3})$.

To evaluate the performance of BR-DTRs, we repeat the simulation 100 times and assess the estimated rules on independent testing data with N=5,000. We apply both the linear and the Gaussian kernel and the estimation following the same setting as Section 4 except that ${\left\{C_{n,t}\right\}}_{t=1}^{4}$ are selected from tuning grid $2^{\left\{-2,-1,0,1,2\right\}}$ via two-folds cross-validation. We repeat the analyses with $\tau =\tau_{1}=\cdots =\tau_{4}$ equal to 4, 5 and 6 respectively and fixing $\eta =\eta_{1}=\cdots =\eta_{4}=0.02$. The complete results are displayed in Table S.3. The findings are similar to Section 4, which indicate that BR-DTRs still yields estimated rules with stagewise risks controlled below or close to the prespecified constraints for more general decision-making problems.

## Appendix D: Flowchart of the Study Design of the DURABLE Trial

**Table S.2:** Estimated reward/risk on independent testing data of the additional simulation study with an unknown and unbalanced design under 3 different methods using linear/Gaussian kernel. Results are reported in the same format as Table 1.

η	Method	Linear Kernel				Gaussian Kernel			
		Reward - II	Risk - II	Cumulative Reward	Risk - I	Reward - II	Risk - II	Cumulative Reward	Risk - I
0.02	BR-DTRs	1.515(0.115)	1.428(0.166)	3.276(0.169)	1.360(0.101)	1.563(0.077)	1.519(0.117)	3.057(0.180)	1.386(0.071)
0.02	Naive	—	—	2.973(0.206)	1.372(0.093)	—	—	2.923(0.169)	1.378(0.080)
0.04	BR-DTRs	1.501(0.139)	1.409(0.206)	3.247(0.188)	1.354(0.112)	1.555(0.082)	1.503(0.121)	3.045(0.182)	1.373(0.070)
0.04	Naive	—	—	2.932(0.227)	1.355(0.100)	—	—	2.901(0.172)	1.357(0.080)
0.06	BR-DTRs	1.486(0.166)	1.381(0.246)	3.223(0.176)	1.314(0.129)	1.551(0.082)	1.496(0.123)	3.045(0.179)	1.360(0.068)
0.06	Naive	—	—	2.910(0.232)	1.328(0.103)	—	—	2.886(0.174)	1.334(0.085)
0.08	BR-DTRs	1.504(0.206)	1.407(0.298)	3.220(0.202)	1.317(0.128)	1.550(0.085)	1.493(0.129)	3.035(0.190)	1.351(0.069)
0.08	Naive	—	—	2.918(0.234)	1.321(0.110)	—	—	2.870(0.180)	1.319(0.087)
0.1	BR-DTRs	1.549(0.261)	1.472(0.388)	3.201(0.231)	1.302(0.130)	1.549(0.084)	1.498(0.127)	3.028(0.177)	1.336(0.075)
0.1	Naive	—	—	2.898(0.240)	1.299(0.112)	—	—	2.855(0.181)	1.304(0.088)
	AOWL	2.382(0.030)	2.798(0.001)	5.309(0.000)	2.876(0.000)	2.400(0.012)	2.798(0.001)	5.309(0.000)	2.876(0.000)

**Table S.3:** Simulation results of the additional personalized promotion example. Results are reported in the same format as Table 1.

τ	Kernel	Cumulative Reward	Wave 1 Cost	Wave 2 Cost	Wave 3 Cost	Wave 4 Cost
4	Linear	14.358(2.455)	4.074(0.942)	4.059(0.086)	4.150(0.112)	4.242(0.236)
4	Gaussian	16.896(1.534)	5.074(0.423)	4.059(0.086)	4.163(0.098)	4.294(0.228)
5	Linear	17.780(1.208)	5.204(0.362)	4.059(0.086)	5.508(0.338)	5.495(0.216)
5	Gaussian	21.353(1.001)	6.146(0.287)	5.210(0.433)	5.562(0.221)	5.428(0.263)
6	Linear	21.532(0.870)	6.168(0.316)	6.213(0.215)	6.468(0.222)	6.360(0.205)
6	Gaussian	23.114(0.620)	6.859(0.289)	6.208(0.192)	6.447(0.232)	6.299(0.191)
∞		25.394	8.972	7.034	7.186	7.051

## References

[R1] Achiam Joshua, Held David, Tamar Aviv, and Abbeel Pieter. Constrained Policy Optimization. In Proceedings of the 34th International Conference on Machine Learning, volume 70, pages 22–31. PMLR, 2017.

[R2] Amani Sanae, Alizadeh Mahnoosh, and Thrampoulidis Christos. Linear Stochastic Bandits under Safety Constraints. Advances in Neural Information Processing Systems, 32, 2019.

[R3] Amani Sanae, Thrampoulidis Christos, and Yang Lin. Safe Reinforcement Learning with Linear Function Approximation. In Proceedings of the 38th International Conference on Machine Learning, volume 139, pages 243–253. PMLR, 2021.

[R4] American Diabetes Association. Pharmacologic Approaches to Glycemic Treatment: Standards of Medical Care in Diabetes—2022. Diabetes Care, 44:S111–S124, 2022. 10.2337/dc21-S009 33298420

[R5] Audibert Jean-Yves and Tsybakov Alexandre B.. Fast Learning Rates for Plug-in Classifiers. The Annals of Statistics, 35(2), 2007.

[R6] Barrett Jessica K., Henderson Robin, and Rosthøj Susanne. Doubly Robust Estimation of Optimal Dynamic Treatment Regimes. Statistics in Biosciences, 6(2):244–260, 2014. 25484995 10.1007/s12561-013-9097-6 PMC4245503

[R7] Bellman Richard. Dynamic Programming. Science, 153(3731):34–37, 1966. 17730601 10.1126/science.153.3731.34

[R8] Blatt Doron, Murphy Susan A., and Zhu Ji. Technical Report 04-63. The Methodology Center, Penn. State University, 2004.

[R9] Bohez Steven, Abdolmaleki Abbas, Neunert Michael, Buchli Jonas, Heess Nicolas, and Hadsell Raia. Value Constrained Model-Free Continuous Control. arXiv preprint arXiv:1902.04623, 2019.

[R10] Bura Archana, Hasanzadezonuzy Aria, Kalathil Dileep, Shakkottai Srinivas, and Chamberland Jean-Francois. DOPE: Doubly Optimistic and Pessimistic Exploration for Safe Reinforcement Learning. In Advances in Neural Information Processing Systems, volume 35, pages 1047–1059. Curran Associates, Inc., 2022.

[R11] Butler Emily L., Laber Eric B., Davis Sonia M., and Kosorok Michael R.. Incorporating Patient Preferences into Estimation of Optimal Individualized Treatment Rules. Biometrics, 74(1):18–26, 2018. 28742260 10.1111/biom.12743 PMC5785589

[R12] Chakraborty Bibhas and Moodie Erica E.M.. Statistical Methods for Dynamic Treatment Regimes. Springer, 2013.

[R13] Chakraborty Bibhas and Murphy Susan A.. Dynamic Treatment Regimes. Annual Review of Statistics and Its Application, 1(1):447–464, 2014. 10.1146/annurev-statistics-022513-115553 PMC4231831 25401119

[R14] Chen Fanglin, Liu Xiao, Tang Bo, Xiong Feiyu, Hwang Serim, and Zhuang Guomian. BCRLSP: An Offline Reinforcement Learning Framework for Sequential Targeted Promotion. arXiv preprint arXiv:2207.07790, 2022.

[R15] Chow Yinlam, Ghavamzadeh Mohammad, Janson Lucas, and Pavone Marco. Risk-Constrained Reinforcement Learning with Percentile Risk Criteria. Journal of Machine Learning Research, 18(167):1–51, 2018.

[R16] Cryer Philip E., Davis Stephen N., and Shamoon Harry. Hypoglycemia in Diabetes. Diabetes Care, 26(6):1902–1912, 2003. 12766131 10.2337/diacare.26.6.1902

[R17] Dawson Ree and Lavori Philip W.. Efficient Design and Inference for Multistage Randomized Trials of Individualized Treatment Policies. Biostatistics, 13(1):142–152, 2012. 21765180 10.1093/biostatistics/kxr016 PMC3276275

[R18] Deng Zhun, Sun He, Wu Steven, Zhang Linjun, and Parkes David. Reinforcement Learning with Stepwise Fairness Constraints. In Proceedings of The 26th International Conference on Artificial Intelligence and Statistics, volume 206, pages 10594–10618. PMLR, 2023.

[R19] Ding Dongsheng, Wei Xiaohan, Yang Zhuoran, Wang Zhaoran, and Jovanovic Mihailo. Provably Efficient Safe Exploration via Primal-Dual Policy Optimization. In International Conference on Artificial Intelligence and Statistics, pages 3304–3312. PMLR, 2021.

[R20] Fahrbach Jessie, Jacober Scott, Jiang Honghua, and Martin Sherry. The DURABLE Trial Study Design: Comparing the Safety, Efficacy, and Durability of Insulin Glargine to Insulin Lispro Mix 75/25 Added to Oral Antihyperglycemic Agents in Patients with Type 2 Diabetes. Journal of Diabetes Science and Technology, 2(5):831–838, 2008. 19885269 10.1177/193229680800200514 PMC2769780

[R21] Fei Yingjie, Yang Zhuoran, Chen Yudong, Wang Zhaoran, and Xie Qiaomin. Risk-Sensitive Reinforcement Learning: Near-Optimal Risk-Sample Tradeoff in Regret. Advances in Neural Information Processing Systems, 33:22384–22395, 2020.

[R22] Garcıa Javier and Feráandez Fernando. A Comprehensive Survey on Safe Reinforcement Learning. Journal of Machine Learning Research, 16(1):1437–1480, 2015.

[R23] Geibel Peter and Wysotzki Fritz. Risk-Sensitive Reinforcement Learning Applied to Control under Constraints. Journal of Artificial Intelligence Research, 24:81–108, 2005.

[R24] Ginsburg Geoffrey S. and Phillips Kathryn A.. Precision Medicine: From Science To Value. Health Affairs, 37(5):694–701, 2018. 29733705 10.1377/hlthaff.2017.1624 PMC5989714

[R25] Goldenberg Dmitri, Albert Javier, and Tsype Guy. Optimization Levers for Promotions Personalization Under Limited Budget. In MORS@ RecSys, 2021.

[R26] Hayes Conor F., Rădulescu Roxana, Bargiacchi Eugenio, Källström Johan, Macfarlane Matthew, Reymond Mathieu, Verstraeten Timothy, Zintgraf Luisa M., Dazeley Richard, Heintz Fredrik, Howley Enda, Irissappane Athirai A., Mannion Patrick, Nowé Ann, Ramos Gabriel, Restelli Marcello, Vamplew Peter, and Roijers Diederik M.. A Practical Guide to Multi-Objective Reinforcement Learning and Planning. Autonomous Agents and Multi-Agent Systems, 36(1):26, 2022.

[R27] Henderson Robin, Ansell Phil, and Alshibani Deyadeen. Regret-Regression for Optimal Dynamic Treatment Regimes. Biometrics, 66(4):1192–1201, 2010. 20002404 10.1111/j.1541-0420.2009.01368.x

[R28] Hodson Richard. Precision Medicine. Nature, 537(7619):S49–S49, 2016. 27602738 10.1038/537S49a

[R29] Kalagarla Krishna C., Jain Rahul, and Nuzzo Pierluigi. A Sample-Efficient Algorithm for Episodic Finite-Horizon MDP with Constraints. Proceedings of the AAAI Conference on Artificial Intelligence, 35(9):8030–8037, 2021.

[R30] Khezeli Kia and Bitar Eilyan. Safe Linear Stochastic Bandits. Proceedings of the AAAI Conference on Artificial Intelligence, 34(06):10202–10209, 2020.

[R31] Laber Eric B., Lizotte Daniel J., and Ferguson Bradley. Set-Valued Dynamic Treatment Regimes for Competing Outcomes. Biometrics, 70(1):53–61, 2014. 24400912 10.1111/biom.12132 PMC3954452

[R32] Laber Eric B., Wu Fan, Munera Catherine, Lipkovich Ilya, Colucci Salvatore, and Ripa Steve. Identifying Optimal Dosage Regimes under Safety Constraints: An Application to Long Term Opioid Treatment of Chronic Pain. Statistics in Medicine, 37(9):1407–1418, 2018. 29468702 10.1002/sim.7566 PMC6293986

[R33] Le Hoang, Voloshin Cameron, and Yue Yisong. Batch Policy Learning under Constraints. In Proceedings of the 36th International Conference on Machine Learning, volume 97, pages 3703–3712. PMLR, 2019.

[R34] Ledoux Michel and Talagrand Michel. Probability in Banach Spaces. Springer, 1991.

[R35] Lee Juhee, Thall Peter F., Ji Yuan, and Müller Peter. Bayesian Dose-Finding in Two Treatment Cycles Based on the Joint Utility of Efficacy and Toxicity. Journal of the American Statistical Association, 110(510):711–722, 2015. 26366026 10.1080/01621459.2014.926815 PMC4562700

[R36] Lei Huitan, Nahum-Shani Inbal, Lynch Kevin, Oslin David, and Murphy Susan A.. A “SMART” design for building individualized treatment sequences. Annual review of clinical psychology, 8:21–48, 2012. 10.1146/annurev-clinpsy-032511-143152 PMC3887122 22224838

[R37] Liu Ying, Wang Yuanjia, Kosorok Michael R., Zhao Yingqi, and Zeng Donglin. Augmented Outcome-Weighted Learning for Estimating Optimal Dynamic Treatment Regimens. Statistics in Medicine, 37(26):3776–3788, 2018. 29873099 10.1002/sim.7844 PMC6191367

[R38] Ma Haitong, Guan Yang, Li Shegnbo Eben, Zhang Xiangteng, Zheng Sifa, and Chen Jianyu. Feasible Actor-Critic: Constrained Reinforcement Learning for Ensuring Statewise Safety. arXiv preprint arXiv:2105.10682, 2021.

[R39] Ma Haixu, Zeng Donglin, and Liu Yufeng. Learning Individualized Treatment Rules with Many Treatments: A Supervised Clustering Approach Using Adaptive Fusion. In Advances in Neural Information Processing Systems, volume 35, pages 15956–15969. Curran Associates, Inc., 2022.

[R40] Ma Haixu, Zeng Donglin, and Liu Yufeng. Learning Optimal Group-structured Individualized Treatment Rules with Many Treatments. Journal of Machine Learning Research, 24(102):1–48, 2023. PMC10426767 37588020

[R41] Mahdavi Mehrdad, Jin Rong, and Yang Tianbao. Trading Regret for Efficiency: Online Convex Optimization with Long Term Constraints. The Journal of Machine Learning Research, 13(1):2503–2528, 2012.

[R42] Moodie Erica E. M., Chakraborty Bibhas, and Kramer Michael S.. Q-learning for Estimating Optimal Dynamic Treatment Rules from Observational Data. Canadian Journal of Statistics, 40(4):629–645, 2012. 10.1002/cjs.11162 PMC3551601 23355757

[R43] Moradipari Ahmadreza, Thrampoulidis Christos, and Alizadeh Mahnoosh. Stage-wise Conservative Linear Bandits. In Advances in Neural Information Processing Systems, volume 33, pages 11191–11201. Curran Associates, Inc., 2020.

[R44] Murphy Susan A.. Optimal Dynamic Treatment Regimes. Journal of the Royal Statistical Society: Series B (Statistical Methodology), 65(2):331–355, 2003.

[R45] Murphy Susan A.. An Experimental Design for the Development of Adaptive Treatment Strategies. Statistics in Medicine, 24(10):1455–1481, 2005. 15586395 10.1002/sim.2022

[R46] Paternain Santiago, Calvo-Fullana Miguel, Chamon Luiz F. O., and Ribeiro Alejandro. Safe Policies for Reinforcement Learning via Primal-Dual Methods. IEEE Transactions on Automatic Control, 68(3):1321–1336, 2023.

[R47] Qi Zhengling, Liu Dacheng, Fu Haoda, and Liu Yufeng. Multi-Armed Angle-Based Direct Learning for Estimating Optimal Individualized Treatment Rules With Various Outcomes. Journal of the American Statistical Association, 115(530):678–691, 2020. 34219848 10.1080/01621459.2018.1529597 PMC8248273

[R48] Qian Min and Murphy Susan A.. Performance Guarantees for Individualized Treatment Rules. The Annals of Statistics, 39(2):1180–1210, 2011. 21666835 10.1214/10-AOS864 PMC3110016

[R49] Ray Alex, Achiam Joshua, and Amodei Dario. Benchmarking Safe Exploration in Deep Reinforcement Learning. arXiv preprint arXiv:1910.01708, 7(1):2, 2019.

[R50] Robins James M.. Causal Inference from Complex Longitudinal Data. In Latent Variable Modeling and Applications to Causality, pages 69–117. Springer, 1997.

[R51] Robins James M.. Optimal Structural Nested Models for Optimal Sequential Decisions. In Proceedings of the Second Seattle Symposium in Biostatistics: analysis of correlated data, pages 189–326. Springer, 2004.

[R52] Rosthøj Susanne, Fullwood Catherine, Henderson Robin, and Stewart Syd. Estimation of Optimal Dynamic Anticoagulation Regimes from Observational Data: A Regret-Based Approach. Statistics in Medicine, 25(24):4197–4215, 2006. 16981226 10.1002/sim.2694

[R53] Rubin Donald B.. Bayesian Inference for Causal Effects: The Role of Randomization. The Annals of Statistics, 6(1):34–58, 1978.

[R54] Steinwart Ingo and Scovel Clint. Fast Rates for Support Vector Machines Using Gaussian Kernels. The Annals of Statistics, 35(2):575–607, 2007.

[R55] Steinwart Ingo, Hush Don, and Scovel Clint. An Explicit Description of the Reproducing Kernel Hilbert Spaces of Gaussian RBF Kernels. IEEE Transactions on Information Theory, 52(10):4635–4643, 2006.

[R56] Tao Pham Dinh and An Le Thi Hoai. Convex Analysis Approach to DC Programming: Theory, Algorithms and Applications. Acta mathematica vietnamica, 22(1):289–355, 1997.

[R57] UKPDS Group. Intensive Blood-Glucose Control with Sulphonylureas or Insulin Compared With Conventional Treatment and Risk of Complications in Patients with Type 2 Diabetes (UKPDS 33). The Lancet, 352(9131):837–853, 1998. 9742976

[R58] van der Vaart Aad W. and Wellner Jon A.. Weak Convergence and Empirical Processes. Springer, 1996.

[R59] Van Moffaert Kristof and Nowé Ann. Multi-Objective Reinforcement Learning Using Sets of Pareto Dominating Policies. The Journal of Machine Learning Research, 15(1):3483–3512, 2014.

[R60] Wainwright Martin J.. High-Dimensional Statistics: A Non-Asymptotic Viewpoint. Cambridge University Press, 2019.

[R61] Wang Chao, Shi Xiaowei, Xu Shuai, Wang Zhe, Fan Zhiqiang, Feng Yan, You An, and Chen Yu. A Multi-stage Framework for Online Bonus Allocation Based on Constrained User Intent Detection. In Proceedings of the 29th ACM SIGKDD Conference on Knowledge Discovery and Data Mining, pages 5028–5038. ACM, 2023.

[R62] Wang Yuanjia, Fu Haoda, and Zeng Donglin. Learning Optimal Personalized Treatment Rules in Consideration of Benefit and Risk: With an Application to Treating Type 2 Diabetes Patients With Insulin Therapies. Journal of the American Statistical Association, 113(521):1–13, 2018. 30034060 10.1080/01621459.2017.1303386 PMC6051551

[R63] Wei Honghao, Liu Xin, and Ying Lei. A Provably-Efficient Model-Free Algorithm for Constrained Markov Decision Processes. arXiv preprint arXiv:2106.01577, 2021.

[R64] Wu Yichao, Zhang Hao Helen, and Liu Yufeng. Robust Model-Free Multiclass Probability Estimation. Journal of the American Statistical Association, 105(489):424–436, 2010. 21113386 10.1198/jasa.2010.tm09107 PMC2990887

[R65] Yang Runzhe, Sun Xingyuan, and Narasimhan Karthik. A Generalized Algorithm for Multi-Objective Reinforcement Learning and Policy Adaptation. Advances in Neural Information Processing Systems, 32, 2019.

[R66] Yu Ming, Yang Zhuoran, Kolar Mladen, and Wang Zhaoran. Convergent Policy Optimization for Safe Reinforcement Learning. Advances in Neural Information Processing Systems, 32, 2019.

[R67] Zhang Baqun, Tsiatis Anastasios A., Davidian Marie, Zhang Min, and Laber Eric B.. Estimating Optimal Treatment Regimes from a Classification Perspective: Treatment Regimes and Classification. Stat, 1(1):103–114, 2012. 23645940 10.1002/sta.411 PMC3640350

[R68] Zhao Weiye, He Tairan, Chen Rui, Wei Tianhao, and Liu Changliu. State-wise Safe Reinforcement Learning: A Survey. arXiv preprint arXiv:2302.03122, 2023.

[R69] Zhao Yingqi, Zeng Donglin, Rush A. John, and Kosorok Michael R.. Estimating Individualized Treatment Rules Using Outcome Weighted Learning. Journal of the American Statistical Association, 107(499):1106–1118, 2012. 23630406 10.1080/01621459.2012.695674 PMC3636816

[R70] Zhao Yingqi, Zeng Donglin, Laber Eric B., and Kosorok Michael R.. New Statistical Learning Methods for Estimating Optimal Dynamic Treatment Regimes. Journal of the American Statistical Association, 110(510):583–598, 2015. 26236062 10.1080/01621459.2014.937488 PMC4517946

